# National-, institutional-, and individual-level determinants of dermatologic research excellence: an analysis of Stanford–Elsevier Lists of the top 2% scholars worldwide (2017–2023)

**DOI:** 10.3389/fmed.2026.1728400

**Published:** 2026-04-13

**Authors:** Abanoub Riad, Michal Koščík

**Affiliations:** 1 Masaryk Centre for Global Health (MCGH), Department of Public Health, Faculty of Medicine, Masaryk University, Brno, Czechia; 2 Department of Public Health, Faculty of Medicine, Masaryk University, Brno, Czechia

**Keywords:** bibliometrics, dermatology, global health, research institutions, research personnel

## Abstract

**Background:**

Skin diseases contribute to a massive and often overlooked component of the global disease burden, highlighting the need for a better understanding of the dermatologic research landscape and its key drivers of excellence.

**Aim:**

To explore the national, institutional, and individual-level determinants that shape dermatologic research excellence worldwide.

**Methods:**

We analyzed the publicly available Stanford–Elsevier Lists of the top 2% most-cited scientists (2017–2023), extracting scholars classified in *Dermatology & Venereal Diseases* to identify excellent dermatologic scholars (EDS). EDS records were then linked, based on affiliation data, to country-level indicators (Universal Health Coverage [UHC], Human Development Index [HDI], Gender Inequality Index [GII], national budgets, and disease burden), institutional rankings (Quacquarelli Symonds [QS], Times Higher Education [THE], and Academic Ranking of World Universities [ARWU]), and individual attributes (gender and academic age). Outcomes were EDS counts by country/institution and scholar-level bibliometrics (citations excluding self-citations, modified *H*-index, and composite score).

**Results:**

EDS were overwhelmingly based in high-income countries (97.9% *career-long*; 94.5% *single-year*) with the EURO region contributing ~48% of EDS and exhibiting highest densities (0.585 and 0.482 per 100,000), while low-income settings had ~0.002. The top 20 institutions hosted ~21% of all EDS. Women comprised 22.9% (*career-long*) and 28.6% (*single-year*) of EDS; men had higher median citations and modified *H*-indices. Academic age correlated positively with modified *H*-index (*ρ* = 0.312 *career-long*) and C-score (*ρ* = 0.145 *single-year*), and each additional year predicted higher citations (*β* = 84.1 *career-long*; *β* = 2.6 *single-year*). In adjusted models, higher HDI and UHC aligned with higher citation counts.

**Conclusion:**

Dermatologic research excellence remains concentrated in high-income, predominantly European and Anglophone ecosystems, within a small cadre of elite institutions and among older, male scholars. Policymakers should focus on targeted funding for under-represented regions and institutional reforms to ensure equitable career advancement for women in academic dermatology.

## 1 Introduction

Skin diseases constitute a massive and often overlooked component of the global disease burden, profoundly affecting health and economic productivity worldwide (1). In February 2025, the World Health Organization (WHO) issued a landmark resolution (EB156/24) recognizing skin diseases as a global public health priority and urging Member States to strengthen prevention, surveillance, and management within Universal Health Coverage (UHC) frameworks (2). The resolution called for enhanced investments in dermatologic research and innovation, improved access to essential diagnostics and medicines, and the integration of skin health into primary care systems, particularly in low- and middle-income countries (LMICs) (2). The most recent data from the Global Burden of Disease (GBD) study estimate that viral skin diseases alone accounted for over 4.2 million disability-adjusted life years (DALYs) in 2021 (3). Dermatological conditions, including chronic disorders such as psoriasis, dermatitis, and acne vulgaris, also impose substantial economic costs, with studies in the United States estimating their cumulative burden at approximately USD 75 billion annually (4).

Despite this immense global health and economic impact, scientific knowledge synthesis in dermatology remains profoundly imbalanced (5). Decades of bibliometric analyses demonstrate that the vast majority of dermatologic research, publications, and resources originate from high-income countries (HICs), particularly the United States and Western Europe (6). This geographic asymmetry underscores a critical mismatch between global epidemiological needs and available scientific research capacity, perpetuating a research agenda shaped more by market dynamics than by population health priorities (7, 8).

Beyond these documented disparities in dermatologic evidence generation, the current understanding of dermatology research remains limited by several methodological and conceptual constraints. Most existing analyses have prioritized research quantity, such as publication counts or citation totals, rather than examining research quality or excellence (7). Nevertheless, research excellence increasingly underpins institutional evaluation and funding frameworks; therefore, a clearer understanding of its determinants and global distribution is essential to guide more equitable, evidence-informed support mechanisms and to promote a fairer, more balanced dermatologic research landscape (9, 10).

The *science-wide author databases of standardized citation indicators*, developed by Ioannidis and colleagues, provide a comprehensive framework for assessing research excellence across disciplines (11). These databases, commonly referred to as the Stanford–Elsevier Lists (SEL), employ a composite citation indicator (C-score) that integrates multiple bibliometric dimensions, including total citations, *H*-index, co-authorship-adjusted impact, and author position (11, 12). The C-score adjusts for self-citations and field-specific citation practices, allowing fairer comparisons across diverse research domains. By distinguishing between *career-long* impact, reflecting cumulative scholarly influence, and *single-year* impact, capturing recent contributions, the SEL offers a nuanced and transparent measure of academic excellence (11). Owing to its methodological rigor, reproducibility, and ongoing updates, it currently represents the most reliable and widely recognized source for evaluating individual and institutional research performance worldwide (13).

The present study employs the SEL of the top 2% most-cited scholars worldwide to identify cohorts of excellent dermatologic scholars (EDS) included in successive annual updates between 2017 and 2023. Its overarching aim is to examine the global distribution and determinants of dermatologic research excellence. The primary objectives are (a) to analyze national- and institutional-level determinants of research excellence, operationalized as the number of EDS per country and institution, and (b) to assess individual-level determinants, namely gender and academic age, in relation to their influence on scholarly performance. The secondary objectives are (a) to explore factors associated with female representation among EDS at the national level and (b) to evaluate temporal trends in EDS counts across recent years.

## 2 Materials and methods

### 2.1 Study design

**Figure 1:**
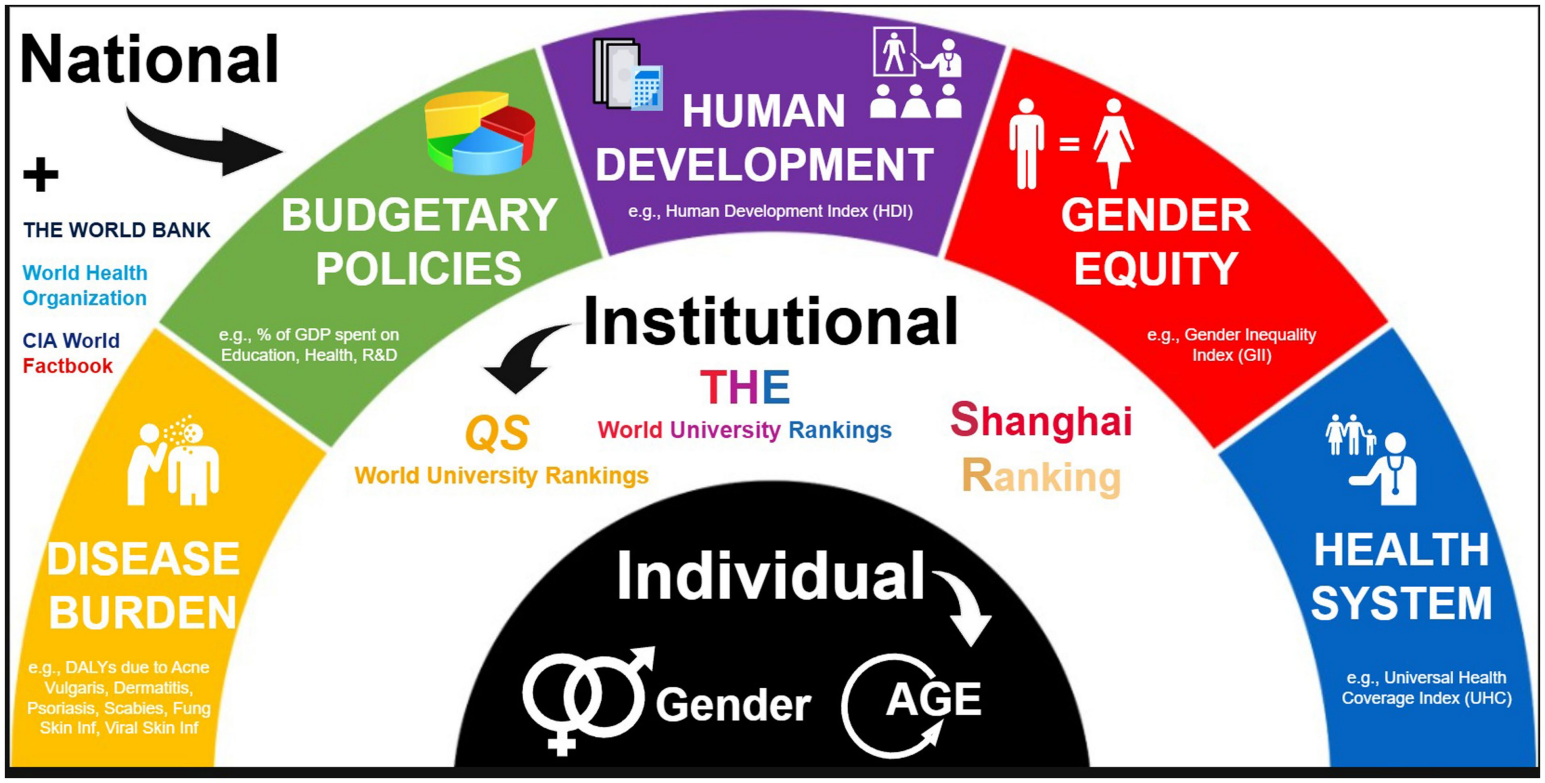
Theoretical framework of multilevel determinants shaping dermatologic research excellence: individual-, institutional-, and national-level predictors.

A bibliometric design was employed, guided by an ecological framework comprising three separate levels of determinants influencing dermatologic research excellence. At the national level, determinants encompassed health system attributes, indicators of gender equity, human development indices, fiscal policies, and the burden of disease. The institutional level included both general and field-specific university rankings. The individual level considered gender and academic age. These levels are illustrated in the conceptual framework presented in Figure 1. The design and reporting were undertaken in line with the BIBLIO guidelines (*Checklist for Bibliometric Reviews of the Biomedical Literature*) (14).

### 2.2 Data sources

The core dataset utilised was the *science-wide author databases of standardized citation indicators*, referred to as the Stanford–Elsevier Lists (SEL) (11, 15). Seven successive releases, spanning the years 2017–2023, were incorporated into the analysis. This period corresponds to the full range of consistently available SEL releases at the time of data extraction (July 2025). Both *career-long* (2017–2023) and *single-year* (2017, 2019–2023) datasets were included, all accessed via the Elsevier Data Repository® (12).

Additional sources were used to assemble national and institutional indicators, as follows:

- Health system variables, including the Universal Health Coverage Index (UHC), provided by the World Health Organization (WHO) data repository (16).
- Gender equity indicators, including the Gender Inequality Index (GII), from the United Nations Development Programme (UNDP) Human Development Reports (17).
- Human development indicators, including the Human Development Index (HDI), from the UNDP Human Development Reports (18).
- Budgetary policy data, including the gross domestic product (GDP) proportions allocated to education, health, and R&D, retrieved from the World Bank Open Data platform (19).
- Disease burden indicators, including DALYs attributed to acne vulgaris, dermatitis and psoriasis extracted from the Global Burden of Disease (GBD) 2021 database (20).
- Official language of each country, as documented in the CIA World Factbook (21).

Institutional performance metrics obtained from QS, the Times Higher Education (THE), and the Academic Ranking of World Universities (ARWU) by ShanghaiRanking (22–24).

### 2.3 Data cleaning and pre-processing

The SEL datasets for *career-long* and *single-year* classifications were first filtered to retain only scholars assigned to *Dermatology & Venereal Diseases* under either subfield 1 or subfield 2. Records outside this discipline were omitted.

Gender determination was conducted with *Genderize.io* (Demografix ApS, Roskilde, Denmark), which infers gender from first names using contextual country information (25). The tool’s predictions are based on more than 900 million associations compiled from social media platforms (26). Within the extracted datasets, 17,212 records were assessed. Gender attribution was unsuccessful for 2427 cases (14.1%), largely attributable to single-letter initials or names too rare to satisfy the 99% certainty threshold.

As a final measure, institutional information was systematically reviewed and harmonized. Discrepancies across native-language versions, acronyms, and English transliterations were corrected, while duplicate entries were standardized and consolidated.

### 2.4 Independent variables

National-level determinants (*n* = 28) encompassed five domains:

1. Health system attributes comprised the universal health coverage index (UHC) and general government expenditure on health (%), reflecting service provision and financial investment.
2. Gender equity was assessed using the gender inequality index (GII), maternal mortality per 100,000 live births, adolescent birth rate (per 1,000 women aged 15–19), education gap among those aged 25 + (male−female), employment gap among those aged 15 + (male−female), and female share of parliamentary seats (%).
3. Human development indicators included the human development index (HDI), life expectancy at birth (years), expected years of schooling, mean years of schooling, and gross national income per capita (USD).
4. Budgetary priorities were represented by % GDP spent on research and development, % GDP spent on health, and % GDP spent on education.
5. Disease burden was measured by DALYs attributed to acne vulgaris, dermatitis, psoriasis, scabies, fungal skin infections, and viral skin infections.

In addition, World Bank level, WHO region, and official language were incorporated as contextual determinants.

Institutional determinants (*n* = 12) were obtained from QS [pharmacy (overall score, academic reputation), general (overall score, academic reputation)], THE [medicine (overall score, research quality), general (overall score, research quality)], and ARWU [medicine (overall score, research impact), general (overall score, per capita performance)].

Individual determinants (*n* = 2) were gender, inferred by the *Genderize.io* tool, and academic age, operationalized as the number of years between the first and most recent Scopus-indexed publication. Academic age was not available in the 2017 *single-year* SEL and was therefore treated as missing for that release.

### 2.5 Dependent variables

The primary dependent measure was the number of excellent dermatologic scholars (EDS) per country and per institution, derived from both the *career-long* and *single-year* SEL.

Four core bibliometric outcomes were employed to indicate research excellence: (a) citation count excluding self-citations; (b) modified *H*-index, defined as an adjusted version of the *H*-index that incorporates the number of co-authors per publication while excluding self-citations; (c) composite score (C-score), a SEL-specific indicator integrating six citation-based metrics, adjusted for field and authorship position, with self-citations excluded; and (d) percentage of self-citations.

To provide further insights related to academic age, additional authorship role-specific indicators were included. These comprised the number and citation count of (a) single-authored publications, (b) single- and first-authored publications, and (c) single-, first-, and last-authored publications.

### 2.6 Statistical analyses

Descriptive statistics were used as the first analytical step. Categorical variables, exemplified by WHO region, and ordinal variables, such as World Bank level, were described as frequencies (*n*) and percentages (*%*). Numerical outcomes, including citation counts, were reported as medians and interquartile ranges (IQR). The Shapiro–Wilk test was used to assess distributional normality, with *p*-values <0.05 interpreted as evidence of non-normal distribution. Univariable analyses were then conducted to explore associations. Chi-squared tests, Fisher’s exact tests, Mann–Whitney *U* tests, and Spearman’s rho correlations were applied as appropriate, with significance set at <0.05.

Regression analyses were subsequently carried out with four aims: (a) simple logistic regression was used to model female gender group membership; (b) linear regression models were fitted to assess the effect of academic age on bibliometric outcomes; (c) linear regression models for core bibliometric outcomes were developed with national-level determinants as predictors, adjusted simultaneously for gender and academic age; and (d) multivariable linear regression models were established for core bibliometric outcomes including both individual-level and thematic national-level determinants simultaneously, to disentangle their independent contributions.

## 3 Results

The dataset comprised 17,212 EDS records, of which 9,193 (53.4%) were drawn from the *career-long* SEL and 8,019 (46.6%) from the *single-year* SEL. Information availability was high, with country affiliation identified for 98.3% of EDS, institutional affiliation for 98.7%, gender for 85.9%, and academic age for 95.9%. The number of EDS documented in the SEL expanded steadily, increasing in the *career-long* SEL from 974 in 2017 to 1,648 in 2023, and in the *single-year* SEL from 698 to 1,660 during the same period.

### 3.1 National-level analyses of dermatologic research excellence

High-income countries accounted for nearly all EDS, representing 97.9% in the *career-long* SEL and 94.5% in the *single-year* SEL. By WHO region, EURO contributed the largest proportion (47.5% and 47.9%), whereas AFRO contributed the smallest (0.18% and 0.32%). In terms of language distribution, English-speaking countries held the greatest share (54.9% and 47.2%), followed by German-speaking countries (16.9% and 17.6%) and Japanese-speaking countries (5.1% and 5.9%) (Table 1). At the country level, the United States contributed 39.5% and 33.7% of EDS, followed by Germany (13.7% and 14.3%), the United Kingdom (11.3% and 8.4%), and Japan (5.2% and 5.9%) (Supplementary Tables S1, S2).

**Table 1:** National-level Analysis: Distribution of Dermatologic Scholars and their Citation Counts in the *Career–Long* and *Single–Year* Stanford-Elsevier Lists (SEL) of Top Scientists Worldwide (2017–2023), Stratified by World Bank Classification (FY 2024) and Official Language (CIA World Factbook)

Career–Long SEL										
	Variable	Outcome	SEL 2017	SEL 2018	SEL 2019	SEL 2020	SEL 2021	SEL 2022	SEL 2023	Total ▼
Scholars *N* (%)	World Bank	High	844 (99.18%)	913 (98.70%)	1173 (98.41%)	1377 (97.73%)	1419 (97.33%)	1500 (97.15%)	1595 (97.49%)	8821 (97.85%)
		Upper-middle	3 (0.35%)	9 (0.97%)	11 (0.92%)	23 (1.63%)	30 (2.06%)	33 (2.14%)	32 (1.96%)	141 (1.56%)
		Lower-middle	4 (0.47%)	3 (0.32%)	8 (0.67%)	8 (0.57%)	8 (0.55%)	11 (0.71%)	9 (0.55%)	51 (0.57%)
		Low	0 (0.00%)	0 (0.00%)	0 (0.00%)	1 (0.07%)	1 (0.07%)	0 (0.00%)	0 (0.00%)	2 (0.02%)
	WHO region	EURO	403 (47.36%)	436 (47.14%)	565 (47.40%)	687 (48.76%)	700 (48.01%)	727 (47.09%)	768 (46.94%)	4286 (47.54%)
		AMRO	384 (45.12%)	416 (44.97%)	511 (42.87%)	581 (41.23%)	605 (41.50%)	642 (41.58%)	687 (41.99%)	3826 (42.44%)
		WPRO	59 (6.93%)	68 (7.35%)	105 (8.81%)	127 (9.01%)	137 (9.40%)	155 (10.04%)	163 (9.96%)	814 (9.03%)
		SEARO	2 (0.24%)	1 (0.11%)	6 (0.50%)	7 (0.50%)	7 (0.48%)	11 (0.71%)	8 (0.49%)	42 (0.47%)
		EMRO	3 (0.35%)	3 (0.32%)	5 (0.42%)	4 (0.28%)	5 (0.34%)	5 (0.32%)	6 (0.37%)	31 (0.34%)
		AFRO	0 (0.00%)	1 (0.11%)	0 (0.00%)	3 (0.21%)	4 (0.27%)	4 (0.26%)	4 (0.24%)	16 (0.18%)
	Official language	English	514 (52.77%)	548 (59.12%)	680 (56.71%)	775 (54.54%)	794 (53.98%)	839 (54.02%)	899 (54.55%)	5049 (54.92%)
		German	155 (15.91%)	169 (18.23%)	205 (17.10%)	241 (16.96%)	247 (16.79%)	262 (16.87%)	274 (16.63%)	1553 (16.89%)
		Japanese	37 (3.80%)	42 (4.53%)	60 (5.00%)	75 (5.28%)	77 (5.23%)	87 (5.60%)	88 (5.34%)	466 (5.07%)
		French	39 (4.00%)	45 (4.85%)	61 (5.09%)	67 (4.71%)	73 (4.96%)	70 (4.51%)	72 (4.37%)	427 (4.64%)
		Other	229 (23.51%)	123 (13.27%)	193 (16.10%)	263 (18.51%)	280 (19.03%)	295 (19.00%)	315 (19.11%)	1698 (18.47%)
Citations Median (IQR)	World Bank	High	6431 (4236–10400)	7067 (4740–11420)	6308 (4057–10854)	6335 (3839–10875)	6549 (3903–11354)	7092 (4227–12170)	7361 (4194–12650)	6729 (4139–11471)
		Upper-middle	2790 (2696–4215)	4129 (2793–6284)	2682 (1655–3958)	4534 (2798–6260)	4144 (2982–6077)	4803 (3338–7124)	4653 (3460–7691)	4372 (2872–6691)
		Lower-middle	2801 (2686–3459)	3343 (3286–4172)	3700 (3082–5061)	4023 (3662–5541)	4032 (3593–5760)	4433 (3822–6157)	4703 (4423–6442)	4095 (3426–5558)
		Low	NA	NA	NA	1506 (1506–1506)	1573 (1573–1573)	NA	NA	1540 (1523–1556)
	WHO region	AMRO	6092 (3883–10126)	6390 (4321–10651)	5835 (3518–10157)	5767 (3224–10108)	5884 (3356–10367)	6372 (3566–11020)	6501 (3658–11210)	6094 (3606–10582)
		EURO	6905 (4543–10576)	7816 (5123–11696)	6708 (4419–11282)	6657 (4229–11330)	6916 (4361–11693)	7480 (4664–13118)	7910 (4798–14024)	7194 (4550–12121)
		WPRO	6550 (4454–10106)	7091 (4975–10016)	6771 (4357–10167)	6267 (3868–9973)	6549 (4076–10361)	7124 (4600–11016)	7360 (4644–11238)	6822 (4347–10704)
		SEARO	3886 (3186–4585)	5000 (5000–5000)	4433 (3447–5095)	4095 (3564–5558)	3957 (3568–5788)	4461 (3822–6636)	4800 (4192–6499)	4582 (3542–6020)
		EMRO	2850 (2801–7279)	3343 (3286–4820)	2360 (1674–3577)	3425 (2606–7146)	3053 (1788–4107)	3481 (1916–4433)	2974 (1784–4449)	3230 (1852–4531)
		AFRO	NA	5381 (5381–5381)	NA	5254 (3380–23843)	5214 (3748–16252)	8343 (6970–21263)	9856 (8401–24757)	6824 (4990–18255)
	Official language	English	5856 (3881–9681)	6320 (4398–10595)	5828 (3617–10111)	5646 (3234–9832)	5916 (3371–10196)	6436 (3656–10979)	6582 (3660–11190)	6069 (3655–10456)
		German	8283 (5842–12496)	8922 (6322–13847)	8585 (5638–13060)	8884 (4920–13509)	8899 (4942–14096)	9954 (5508–15594)	10235 (5746–16312)	9104 (5542–14433)
		Japanese	7983 (4835–12433)	8794 (5076–12518)	7428 (5137–11867)	6422 (4212–10978)	6716 (4706–11317)	7327 (4956–12723)	7462 (5112–13043)	7323 (4909–12372)
		French	8281 (5702–10439)	9182 (6634–11824)	8046 (5817–12492)	8821 (6160–13632)	8263 (5611–13983)	9527 (6606–15570)	9610 (6903–16717)	8821 (6147–13890)
		Other	4889 (3415–8028)	6028 (4604–9666)	5559 (3758–9264)	5583 (3464–8668)	5700 (3389–8198)	6150 (3814–9220)	6327 (4070–10352)	5702 (3717–9190)

Single–Year SEL									
	Variable	Outcome	SEL 2017	SEL 2019	SEL 2020	SEL 2021	SEL 2022	SEL 2023	Total ▼
Scholars *N* (%)	World Bank	High	615 (98.09%)	1137 (96.77%)	1358 (95.43%)	1394 (94.51%)	1465 (94.03%)	1545 (93.35%)	7514 (94.96%)
		Upper-middle	9 (1.44%)	28 (2.38%)	47 (3.30%)	64 (4.34%)	66 (4.24%)	79 (4.77%)	293 (3.70%)
		Lower-middle	3 (0.48%)	10 (0.85%)	18 (1.26%)	17 (1.15%)	27 (1.73%)	31 (1.87%)	106 (1.34%)
		Low	0 (0.00%)	0 (0.00%)	0 (0.00%)	0 (0.00%)	0 (0.00%)	0 (0.00%)	0 (0.00%)
	WHO region	EURO	305 (48.64%)	571 (48.60%)	704 (49.47%)	717 (48.61%)	730 (46.85%)	765 (46.22%)	3792 (47.92%)
		AMRO	254 (40.51%)	453 (38.55%)	525 (36.89%)	547 (37.08%)	586 (37.61%)	607 (36.68%)	2972 (37.56%)
		WPRO	63 (10.05%)	133 (11.32%)	162 (11.38%)	174 (11.80%)	198 (12.71%)	226 (13.66%)	956 (12.08%)
		EMRO	3 (0.48%)	9 (0.77%)	15 (1.05%)	18 (1.22%)	17 (1.09%)	24 (1.45%)	86 (1.09%)
		SEARO	1 (0.16%)	6 (0.51%)	13 (0.91%)	13 (0.88%)	22 (1.41%)	27 (1.63%)	82 (1.04%)
		AFRO	1 (0.16%)	3 (0.26%)	4 (0.28%)	6 (0.41%)	5 (0.32%)	6 (0.36%)	25 (0.32%)
	Official language	English	336 (48.14%)	585 (49.16%)	676 (47.21%)	686 (46.38%)	735 (47.12%)	766 (46.14%)	3784 (47.19%)
		German	127 (18.19%)	221 (18.57%)	262 (18.30%)	265 (17.92%)	268 (17.18%)	271 (16.33%)	1414 (17.63%)
		Japanese	33 (4.73%)	65 (5.46%)	79 (5.52%)	85 (5.75%)	96 (6.15%)	111 (6.69%)	469 (5.85%)
		French	35 (5.01%)	57 (4.79%)	66 (4.61%)	71 (4.80%)	75 (4.81%)	80 (4.82%)	384 (4.79%)
		Other	167 (23.93%)	262 (22.02%)	349 (24.37%)	372 (25.15%)	386 (24.74%)	432 (26.02%)	1968 (24.54%)
Citations Median (IQR)	World Bank	High	667 (406–980)	719 (456–1201)	880 (523–1568)	635 (393–1081)	637 (396–1132)	605 (370–1041)	682 (415–1170)
		Upper-middle	377 (223–868)	517 (318–821)	640 (408–988)	408 (284–630)	456 (297–696)	510 (346–696)	497 (318–805)
		Lower-middle	275 (244–312)	404 (300–565)	468 (299–671)	355 (275–510)	401 (295–523)	344 (242–516)	382 (268–544)
		Low	NA	NA	NA	NA	NA	NA	NA
	WHO region	AMRO	612 (361–974)	650 (400–1133)	789 (464–1502)	558 (334–1020)	592 (346–1022)	570 (339–960)	622 (366–1100)
		EURO	688 (450–1000)	772 (506–1276)	960 (591–1604)	683 (427–1146)	674 (420–1176)	657 (418–1140)	733 (456–1238)
		WPRO	654 (413–870)	694 (469–1073)	828 (542–1277)	603 (402–944)	554 (396–970)	522 (364–911)	622 (412–1013)
		EMRO	275 (244–630)	220 (165–301)	374 (246–563)	360 (208–474)	415 (244–588)	397 (223–646)	374 (214–586)
		SEARO	348 (348–348)	404 (248–466)	467 (277–701)	347 (293–510)	402 (316–494)	281 (230–469)	364 (258–502)
		AFRO	377 (377–377)	1025 (700–1893)	1231 (573–4473)	407 (296–1153)	1041 (510–1664)	836 (408–1626)	640 (371–1796)
	Official language	English	601 (359–973)	642 (394–1121)	766 (457–1440)	556 (332–1021)	573 (340–1056)	548 (331–973)	607 (363–1096)
		German	733 (504–1024)	890 (599–1406)	1099 (702–1814)	756 (494–1251)	736 (486–1263)	768 (477–1312)	823 (520–1386)
		Japanese	694 (456–890)	728 (537–1211)	892 (610–1497)	632 (431–1065)	574 (418–1046)	509 (370–908)	661 (428–1072)
		French	777 (612–1052)	1024 (610–1538)	1201 (774–1923)	761 (540–1297)	830 (562–1358)	738 (553–1348)	865 (583–1422)
		Other	550 (300–912)	642 (406–1036)	833 (527–1313)	590 (375–916)	589 (378–962)	546 (355–849)	625 (390–1011)

**Figure 2:**
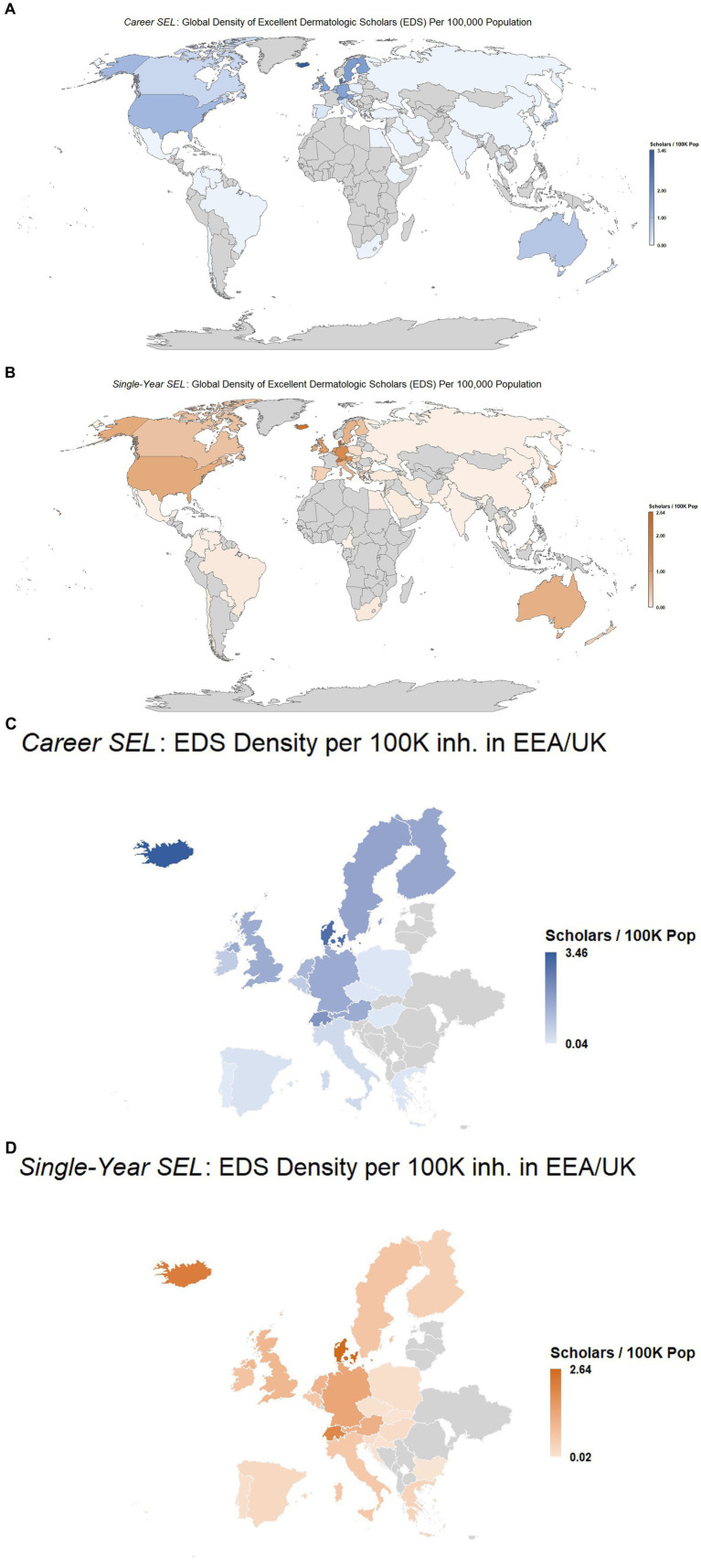
Global density of excellent dermatologic scholars (EDS) in the Stanford–Elsevier Top 2% lists per 100,000 population (2017–2023): **(A)** Career-long global SEL, **(B)** single-year global SEL, **(C)** career-long SEL in EEA/UK, and **(D)** single-year SEL in EEA/UK.

Globally, the density of EDS per 100,000 population was 0.172 in the *career-long* SEL and 0.145 in the *single-year* SEL. High-income countries reported densities of 0.671 and 0.556, compared with 0.002 and not available for low-income countries. Regionally, EURO had the highest densities (0.585 and 0.482), followed by AMRO (0.464 and 0.357) (Table 2). At the country level, Iceland recorded the highest density (3.46 and 2.22), followed by Denmark (3.03 and 2.64) and Switzerland (2.06 and 2.00) (Figure 2).

**Table 2:** National-level analysis: population density of dermatologic scholars and their citation counts in the career-long and single-year Stanford–Elsevier Lists (SEL) of top scientists worldwide (2017–2023), stratified by World Bank classification (FY 2024) and official language (CIA World factbook).

Variable	Outcome	Career-long SEL		Single-year SEL	
		Scholars per 100 K Pop.	Citations per 100 K Pop.	Scholars per 100 K Pop.	Citations per 100 K Pop.
World Bank	High-income	0.671	6,208.592	0.556	567.082
	Upper-middle-income	0.006	41.481	0.013	10.322
	Lower-middle-income	0.003	15.056	0.006	2.967
	Low-income	0.002	2.332	NA	NA
WHO region	AMRO	0.464	3,968.56	0.357	337.766
	EURO	0.585	5,784.849	0.482	530.148
	WPRO	0.048	407.569	0.053	43.789
	SEARO	0.003	14.012	0.017	10.535
	EMRO	0.010	57.07	0.005	2.731
	AFRO	0.008	146.112	0.027	64.682
Official language	English	1.021	8,781.862	0.765	734.27
	German	1.527	17,863.905	1.39	1,659.081
	Japanese	0.376	3,403.109	0.378	335.191
	French	0.623	7,719.813	0.393	566.734
	Other	0.034	255.852	0.039	34.321
Total	*N*/population size	0.172	1,582.064	0.145	145.156

The number of EDS per country showed positive, moderate-to-strong correlations with health system attributes, human development indices, and budgetary policy indicators, while negative associations were observed with the gender inequality index and most of its components. For dermatologic disease burden, all DALYs demonstrated positive correlations, with the exception of DALYs attributed to scabies, which showed a negative association. These relationships were also reflected in the bibliometric outcomes and remained consistent across the *career-long* and *single-year* SEL (Table 3).

**Table 3:** National-level analyses: correlations between health system characteristics, gender equity, human development, budgetary policies, and disease burden, with the number of excellent dermatologic Scholars and their scholarly output metrics in the Stanford–Elsevier Lists (2017–2023).

Domain	Indicator	Scholars *N*	Citations *N*	Modified *H*-index	Composite Score	Self-citation %
Career-long SEL						
Health system	Universal health coverage index (UHC)	0.608**	0.610**	0.188	0.155	0.245
	General government expenditure on health (%)	0.502**	0.496**	0.208	0.209	0.045
Gender equity	Gender inequality index (GII)	−0.604**	−0.610**	−0.284*	−0.280*	−0.178
	Maternal mortality per 100 K live births	−0.467**	−0.469**	−0.352*	−0.268	−0.15
	Adolescent birth rate (*per* 1 K women aged 15–19)	−0.513**	−0.519**	−0.255	−0.211	−0.174
	Education gap 25+ (male−female)	−0.105	−0.178	−0.194	−0.202	−0.064
	Employment gap 15+ (male−female)	−0.561**	−0.597**	−0.203	−0.214	−0.084
	Female share of parliamentary seats (%)	0.353*	0.371**	0.126	0.136	0.019
Human development	Human development index (HDI)	0.641**	0.643**	0.314*	0.313*	0.103
	Life expectancy at birth (years)	0.530**	0.531**	0.202	0.108	0.133
	Expected years of schooling	0.383**	0.387**	0.216	0.172	0.094
	Mean years of schooling	0.573**	0.606**	0.387**	0.439**	0.031
	Gross national income per capita (USD)	0.517**	0.534**	0.249	0.265	0.074
Budgetary policies	% GDP spent on research & development	0.769**	0.784**	0.330*	0.324*	0.191
	% GDP spent on health	0.679**	0.708**	0.316*	0.303*	0.105
	% GDP spent on education	0.323*	0.361**	0.137	0.1	0.143
Disease burden	DALYs attributed to acne vulgaris	0.272	0.274	−0.009	0.06	0.06
	DALYs attributed to dermatitis	0.556**	0.555**	0.078	0.198	0.049
	DALYs attributed to psoriasis	0.444**	0.465**	0.101	0.24	0.047
	DALYs attributed to scabies	−0.463**	−0.497**	−0.2	−0.239	−0.004
	DALYs attributed to fungal skin infections	0.361**	0.407**	0.169	0.026	0.259
	DALYs attributed to viral skin infections	0.505**	0.499**	0.185	0.134	0.066
Single-year SEL						
Health system	Universal health coverage index (UHC)	0.507**	0.449**	0.124	0.172	0.041
	General government expenditure on health (%)	0.442**	0.419**	0.205	0.235	−0.129
Gender equity	Gender inequality index (GII)	−0.395**	−0.341**	−0.07	−0.236	0.057
	Maternal mortality per 100 K live births	−0.285*	−0.241	−0.183	−0.242	0.043
	Adolescent birth rate (*per* 1 K women aged 15–19)	−0.402**	−0.355**	−0.077	−0.11	0.164
	Education gap 25+ (male−female)	−0.008	−0.038	−0.167	−0.406**	0.079
	Employment gap 15+ (male−female)	−0.431**	−0.421**	−0.124	−0.292*	−0.036
	Female share of parliamentary seats (%)	0.263*	0.286*	0.086	0.195	−0.085
Human development	Human development index (HDI)	0.512**	0.459**	0.186	0.221	−0.176
	Life expectancy at birth (years)	0.488**	0.443**	0.147	0.165	−0.163
	Expected years of schooling	0.458**	0.373**	0.133	0.109	−0.119
	Mean years of schooling	0.382**	0.368**	0.228	0.325*	−0.091
	Gross national income per capita (USD)	0.444**	0.409**	0.19	0.238	−0.249
Budgetary policies	% GDP spent on research & development	0.681**	0.644**	0.1	0.087	−0.051
	% GDP spent on health	0.587**	0.562**	0.186	0.251	−0.037
	% GDP spent on education	0.262*	0.254	0.056	0.226	0.014
Disease burden	DALYs attributed to acne vulgaris	0.436**	0.449**	0.128	0.218	−0.031
	DALYs attributed to dermatitis	0.538**	0.530**	0.19	0.345**	0.044
	DALYs attributed to psoriasis	0.449**	0.425**	0.152	0.193	0.04
	DALYs attributed to scabies	−0.495**	−0.476**	−0.228	−0.329*	0.018
	DALYs attributed to fungal skin infections	0.271*	0.283*	−0.025	0.078	0.332*
	DALYs attributed to viral skin infections	0.640**	0.603**	0.281*	0.238	−0.073

**Correlation is significant at the 0.01 level (2-tailed). *Correlation is significant at the 0.05 level (2-tailed).

**Figure 3:**
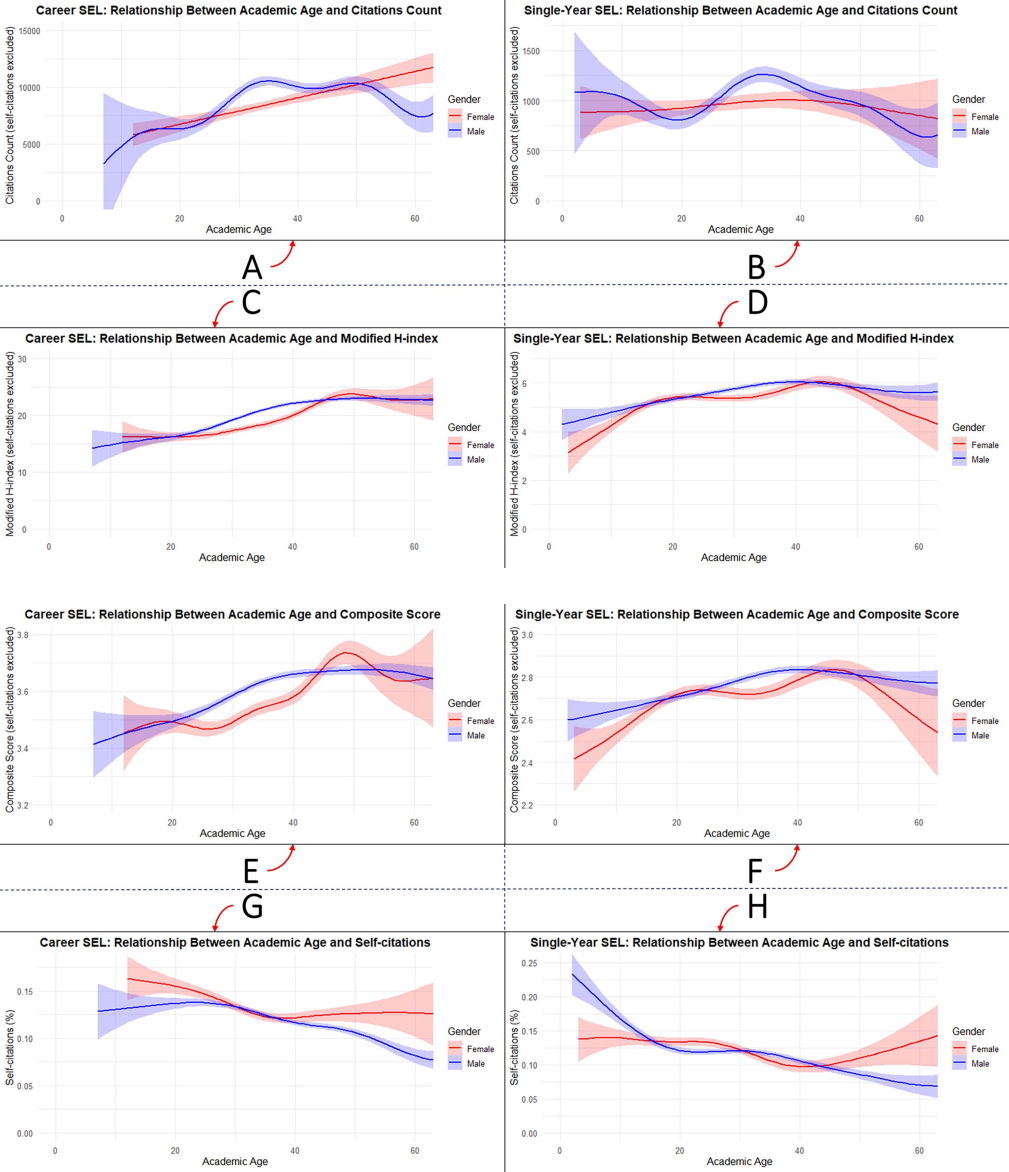
Gender-stratified associations between academic age and scholarly output metrics among excellent dermatologic scholars (EDS) in the Stanford–Elsevier Top 2% Lists (2017–2023): **(A,B)** citation count, **(C,D)** composite score, **(E,F)** modified *h*-index, and **(G,H)** percentage of self-citations for career-long and single-year lists, respectively.

### 3.2 Institutional-level analyses of dermatologic research excellence

The top 20 institutions comprised 21.0% of EDS in the *career-long* SEL and 20.3% in the *single-year* SEL. Alongside US universities, German institutions such as Charité – Berlin University of Medicine, Ludwig Maximilian University of Munich, and the University of Kiel achieved prominent positions in both datasets (Table 4). Correlations between institutional counts and rankings varied by SEL. In the *career-long* SEL, the strongest associations were observed with QS general ranking (0.754) and ARWU medicine ranking (0.736), while in the *single-year* SEL, the highest correlation was with ARWU general ranking (0.715), followed by THE general research quality (0.661) (Table 5).

**Table 4:** Institutional-level analysis: Top 20 institutions hosting dermatologic scholars in the Stanford–Elsevier Lists (SEL) of top scientists worldwide (2017–2023).

Rank	Career-long SEL			Single-year SEL		
	University (acronym)	Country	*N* (%)	University (acronym)	Country	*N* (%)
1	University of California (UC)	USA	313 (3.46%)	University of California (UC)	USA	247 (3.11%)
2	Harvard University (HU)	USA	185 (2.04%)	Harvard University (HU)	USA	150 (1.89%)
3	King’s College London (KCL)	GBR	143 (1.58%)	University of Pennsylvania (UPenn)	USA	115 (1.45%)
4	University of Pennsylvania (UPenn)	USA	107 (1.18%)	Charité – Berlin University of Medicine (Charité)	DEU	110 (1.39%)
5	Mayo Clinic (MC)	USA	98 (1.08%)	Icahn School of Medicine at Mount Sinai (ISMMS)	USA	76 (0.96%)
6	Ludwig Maximilian University of Munich (LMU)	DEU	92 (1.02%)	Northwestern University (NU)	USA	70 (0.88%)
7	Charité – Berlin University of Medicine (Charité)	DEU	82 (0.91%)	University of Michigan (UMich)	USA	73 (0.92%)
8	Yale University (Yale)	USA	82 (0.91%)	Yale University (Yale)	USA	70 (0.88%)
9	New York University (NYU)	USA	81 (0.89%)	Ludwig Maximilian University of Munich (LMU)	DEU	69 (0.87%)
10	Icahn School of Medicine at Mount Sinai (ISMMS)	USA	75 (0.83%)	Mayo Clinic (MC)	USA	67 (0.84%)
11	University of Kiel (CAU)	DEU	75 (0.83%)	University of Kiel (CAU)	DEU	64 (0.81%)
12	University of Cincinnati (UC)	USA	74 (0.82%)	University of Miami (UM)	USA	63 (0.79%)
13	University of Michigan (UMich)	USA	73 (0.81%)	University of Copenhagen (UCPH)	DNK	59 (0.74%)
14	University of Texas (UT)	USA	72 (0.80%)	University of Cincinnati (UC)	USA	57 (0.72%)
15	University of Copenhagen (UCPH)	DNK	66 (0.73%)	Scientific Institute for Research, Hospitalization and Healthcare (IRCCS)	ITA	56 (0.71%)
16	Northwestern University (NU)	USA	63 (0.70%)	King’s College London (KCL)	GBR	55 (0.69%)
17	Karolinska Institute (KI)	SWE	58 (0.64%)	Medical University of Vienna (MedUni Vienna)	AUT	54 (0.68%)
18	National Institutes of Health (NIH)	USA	57 (0.63%)	University of Texas (UT)	USA	54 (0.68%)
19	University of Miami (UM)	USA	55 (0.61%)	University of Toronto (UofT)	CAN	51 (0.64%)
20	University of Iowa (UIowa)	USA	54 (0.60%)	Memorial Sloan Kettering Cancer Center (MSKCC)	USA	49 (0.62%)
Total			1905 (21.04%)			1609 (20.28%)

**Table 5:** Institutional-level analyses: correlations between QS, THE, and ARWU scores and the number of excellent dermatologic scholars hosted by the Top 20 institutions in the Stanford–Elsevier Lists (2017–2023).

Database	Indicator	Career-long SEL: rho	Single-year SEL: rho
Quacquarelli symonds (QS)	Pharmacy (overall score)	0.425	0.300
	Pharmacy (academic reputation)	0.588**	0.493*
	General (overall score)	0.754**	0.564*
	General (academic reputation)	0.627*	0.413
Times higher education (THE)	Medicine (overall score)	0.694**	0.467
	Medicine (research quality)	0.564*	0.564*
	General (overall score)	0.704**	0.563*
	General (research quality)	0.629*	0.661**
Academic ranking of world universities (ARWU)	Medicine (overall score)	0.736**	0.392
	Medicine (research impact)	0.391	0.221
	General (overall score)	0.497	0.715*
	General (*per capita* performance)	0.441	0.363

*Correlation is significant at the 0.05 level (2-tailed). **Correlation is significant at the 0.01 level (2-tailed).

### 3.3 Gender-based analyses of dermatologic research excellence

Females represented 22.9% and 28.6% of EDS in the *career-long* and *single-year* SEL, respectively. The highest female shares were observed in high-income countries (23.2% and 28.7%) and within AMRO (23.9% and 31.5%), exceeding those recorded in other World Bank income groups and WHO regions (Table 6).

**Table 6:** Individual-level analysis: gender and academic age of dermatologic scholars in the Stanford–Elsevier Lists (SEL) of top scientists worldwide (2017–2023).

Variable	Outcome	Female			Male			*p*		
		Scholars: *N* (%)	Citations: median (IQR)	Academic age: median (IQR)	Scholars: *N* (%)	Citations: median (IQR)	Academic Age: median (IQR)	Scholars	Citations	Age
Career-long SEL										
Year	SEL 2017	164 (20.0%)	5853.5 (4089–9181)	33 (27.8–38.2)	658 (80.0%)	6405 (4130.8–10522.5)	35 (29–42)	**0.001**	0.174	**0.006**
	SEL 2018	159 (18.8%)	6608 (4711.5–9709)	34 (28.5–41)	689 (81.2%)	7302 (4761–11632)	37 (30–43)		0.222	**0.002**
	SEL 2019	204 (20.6%)	5705.5 (3578–9362.5)	33 (28–38.2)	784 (79.4%)	6336 (4012.2–11039.5)	37 (30–44)		**0.013**	**<0.001**
	SEL 2020	284 (24.6%)	5825.5 (3453.2–9301.5)	32.5 (27–39)	872 (75.4%)	6448.5 (3767.2–10941.2)	37 (31–43.2)		**0.008**	**<0.001**
	SEL 2021	297 (24.3%)	6176 (3743–9800)	33 (28–39)	923 (75.7%)	6687 (3942.5–11900.5)	38 (31–45)		**0.035**	**<0.001**
	SEL 2022	321 (23.9%)	6804 (4308–11224)	34 (29–41)	1021 (76.1%)	7223 (4217–13016)	38 (32–45)		0.126	**<0.001**
	SEL 2023	357 (24.9%)	7195 (4218–11990)	34 (29–41)	1079 (75.1%)	7471 (4290.5–13629)	38 (32–45)		0.324	**<0.001**
World Bank	High	1743 (23.2%)	6330 (4081.5–10200)	34 (28–40)	5777 (76.8%)	7015 (4232–12128)	37 (31–44)	**0.002**	**<0.001**	**<0.001**
	Upper-middle	23 (17.8%)	5815 (3499.5–7305)	28 (24.5–38)	106 (82.2%)	4095 (2633.2–6582.5)	29 (23–39)		0.125	0.710
	Lower-middle	0 (0.0%)	*NA*	*NA*	35 (100.0%)	3909 (3276–5142.5)	34 (23.5–46)		*NA*	*NA*
	Low	0 (NA%)	*NA*	*NA*	0 (NA%)	*NA*	*NA*		*NA*	*NA*
WHO region	AMRO	841 (23.9%)	5826 (3564–9169)	34 (29–40)	2685 (76.1%)	6247 (3661–11104)	39 (33–46)	**<0.001**	**0.002**	**<0.001**
	EURO	804 (23.6%)	7069 (4718.8–11814.2)	32 (27–39)	2600 (76.4%)	7647 (4798.8–12801.5)	36 (30–42)		**0.02**	**<0.001**
	WPRO	117 (17.0%)	5192 (3546–7512)	35 (29–40)	572 (83.0%)	7127.5 (4608.8–10881.5)	35 (28–43)		**<0.001**	0.283
	SEARO	0 (0.0%)	*NA*	*NA*	28 (100.0%)	4278 (3453.2–5627.2)	41 (31.8–47)		*NA*	*NA*
	EMRO	0 (0.0%)	*NA*	*NA*	29 (100.0%)	3053 (1788–4107)	23 (19–27)		*NA*	*NA*
	AFRO	4 (50.0%)	6824 (5780.5–8148.2)	29.5 (28.8–30.2)	4 (50.0%)	52605 (45962.2–60663)	48 (46.8–49)		**0.03**	**0.029**
Official language	English	1059 (23.9%)	5835 (3591.5–9281.5)	34 (29–40)	3371 (76.1%)	6292 (3761.5–11020)	39 (32–45)	**<0.001**	**<0.001**	**<0.001**
	German	233 (18.3%)	7881 (5262–11597)	29 (25–33)	1041 (81.7%)	9590 (5992–15285)	34 (29–41)		**<0.001**	**<0.001**
	Japanese	49 (11.6%)	5012 (3697–6694)	34 (28–39)	373 (88.4%)	7932 (5302–12997)	36 (29–43)		**<0.001**	**0.06**
	French	90 (27.6%)	12269.5 (6565.8–19253.8)	37 (33–42)	236 (72.4%)	8674 (6027–12964)	37 (32.8–42)		**0.006**	0.745
	*Other*	355 (26.1%)	6176 (4112–9727)	35 (28–40)	1005 (73.9%)	5656 (3601–9264)	37 (31–44)		**0.034**	**<0.001**
Total		1786 (22.9%)	6284.5 (4029.2–10129.8)	34 (28–39.8)	6026 (77.1%)	6860.5 (4129.2–11862.8)	37 (31–44)		**<0.001**	**<0.001**
Single-year SEL										
Year	SEL 2017	142 (22.9%)	571 (371.8–843.2)	NA	477 (77.1%)	669 (400–997)	NA	**0.002**	**0.047**	*NA*
	SEL 2019	255 (25.6%)	664 (444.5–1143)	26 (19–34)	742 (74.4%)	726.5 (445.2–1229.5)	32 (24–39)		0.412	**<0.001**
	SEL 2020	341 (29.2%)	809 (508–1335)	27 (20–34)	828 (70.8%)	897 (517.8–1642.2)	31 (23–39)		**0.042**	**<0.001**
	SEL 2021	386 (30.4%)	588.5 (393.2–960.5)	27 (20–33)	884 (69.6%)	629.5 (374.8–1129)	32 (23–40)		0.287	**<0.001**
	SEL 2022	414 (29.3%)	599.5 (397–1001.2)	27 (21–34)	997 (70.7%)	636 (388–1133)	32 (23–40)		0.421	**<0.001**
	SEL 2023	455 (30.2%)	596 (387.5–966)	27 (20–34)	1052 (69.8%)	591 (357–1044.5)	32 (23–41)		0.856	**<0.001**
World Bank	High	1875 (28.7%)	648 (414–1057)	27 (20–34)	4658 (71.3%)	698 (413.2–1212)	32 (24–40)	**0.001**	**0.005**	**<0.001**
	Upper-middle	90 (34.0%)	515.5 (359–692.2)	20 (15–26)	175 (66.0%)	510 (314–835.5)	23 (17–31)		0.829	**0.031**
	Lower-middle	13 (14.0%)	607 (267–702)	25 (19–28)	80 (86.0%)	384 (274.5–516.5)	21 (18–29)		0.406	0.554
	Low	0 (0.0%)	*NA*	*NA*	0 (0.0%)	*NA*	*NA*		*NA*	*NA*
WHO region	AMRO	863 (31.5%)	603 (359–1008)	26 (19–35)	1881 (68.5%)	645 (371–1169)	34 (24–42)	**<0.001**	**0.023**	**<0.001**
	EURO	924 (29.0%)	708 (453–1161.8)	28 (22–33)	2260 (71.0%)	767.5 (462–1281)	32 (24–38.2)		**0.032**	**<0.001**
	WPRO	157 (19.7%)	564 (416–825)	24 (18–32.5)	640 (80.3%)	610.5 (392.5–1007.2)	29 (22–37)		0.297	**<0.001**
	EMRO	17 (20.7%)	597 (395–702)	16 (10–24)	65 (79.3%)	355 (208–469)	18 (16–24.8)		**0.007**	0.183
	SEARO	5 (7.5%)	277 (267–344)	36 (22–36)	62 (92.5%)	376 (261.8–529.5)	23 (19–31)		0.551	0.474
	AFRO	12 (70.6%)	598 (436.2–1454.8)	22 (18.5–28.5)	5 (29.4%)	10191 (9587–10415)	47 (46–49)		**0.002**	0.070
Official language	English	1059 (31.2%)	599 (370–1012.5)	27 (19–35)	2331 (68.8%)	631 (363.5–1168.5)	34 (25–42)	**<0.001**	0.081	**<0.001**
	German	290 (24.4%)	747 (501.2–1245.8)	26 (22–29)	898 (75.6%)	898 (549–1464)	31 (24–36)		**0.002**	**<0.001**
	Japanese	47 (10.9%)	550 (454–687.5)	27 (20–35)	384 (89.1%)	666.5 (414.5–1105)	30 (23–39)		**0.022**	**0.026**
	French	91 (29.9%)	822 (511.5–1596.5)	34 (27–37)	213 (70.1%)	886 (587–1524)	34 (27–39)		0.734	0.553
	*Other*	506 (30.5%)	644 (419.5–1047.2)	27 (19–34)	1154 (69.5%)	601 (372–967.5)	28 (20–37)		**0.036**	**0.019**
Total		1993 (28.6%)	635 (405–1042)	27 (20–34)	4980 (71.4%)	680 (404–1183.5)	32 (23–40)		**0.014**	**<0.001**

Chi-squared (*χ*^2^) test, Fisher’s exact test, and Mann–Whitney (U) test were used with a significance level *p* ≤ 0.05. Bold font refers to statistically significant associations (*p* < 0.05).

At the country level (restricted to countries with >50 EDS), Israel recorded the highest female proportion in the *career-long* SEL (43.5%), whereas Switzerland had the lowest (7.1%). In the *single-year* SEL, Belgium showed the highest female share (65.6%), while Austria had the lowest (7.8%) (Supplementary Tables S3, S4).

Gender-based comparisons of outcomes indicated higher male values on several metrics. Median citation counts were greater among males in both the *career-long* SEL (6860.5 vs. 6284.5; *p* < 0.001) and the *single-year* SEL (680 vs. 635; *p* = 0.014). Median academic age was also higher in males (37 vs. 34 years; p < 0.001, and 32 vs. 27 years; *p* < 0.001, respectively) (Table 6). Core bibliometric outcomes aligned with these patterns: the modified *H*-index was significantly higher for males in the *career-long* (19.3 vs. 17.7; *p* < 0.001) and *single-year* (5.2 vs. 5.0; *p* < 0.001) SEL (Supplementary Table S5).

### 3.4 Age-based analyses of dermatologic research excellence

The median academic age was 37 years (IQR 30–43) in the *career-long* SEL and 30 years (IQR 22–39) in the *single-year* SEL. Among countries with >50 EDS, Spain reported the longest academic age in the *career-long* dataset [41 years (27–45)], while Brazil recorded the lowest [33 years (24.8–46.2)]. In the *single-year* SEL, the Netherlands showed the highest [36 years (28.2–41)], while China reported the youngest values [18 years (15–21)] (Supplementary Tables S6, S7).

**Figure 4:**
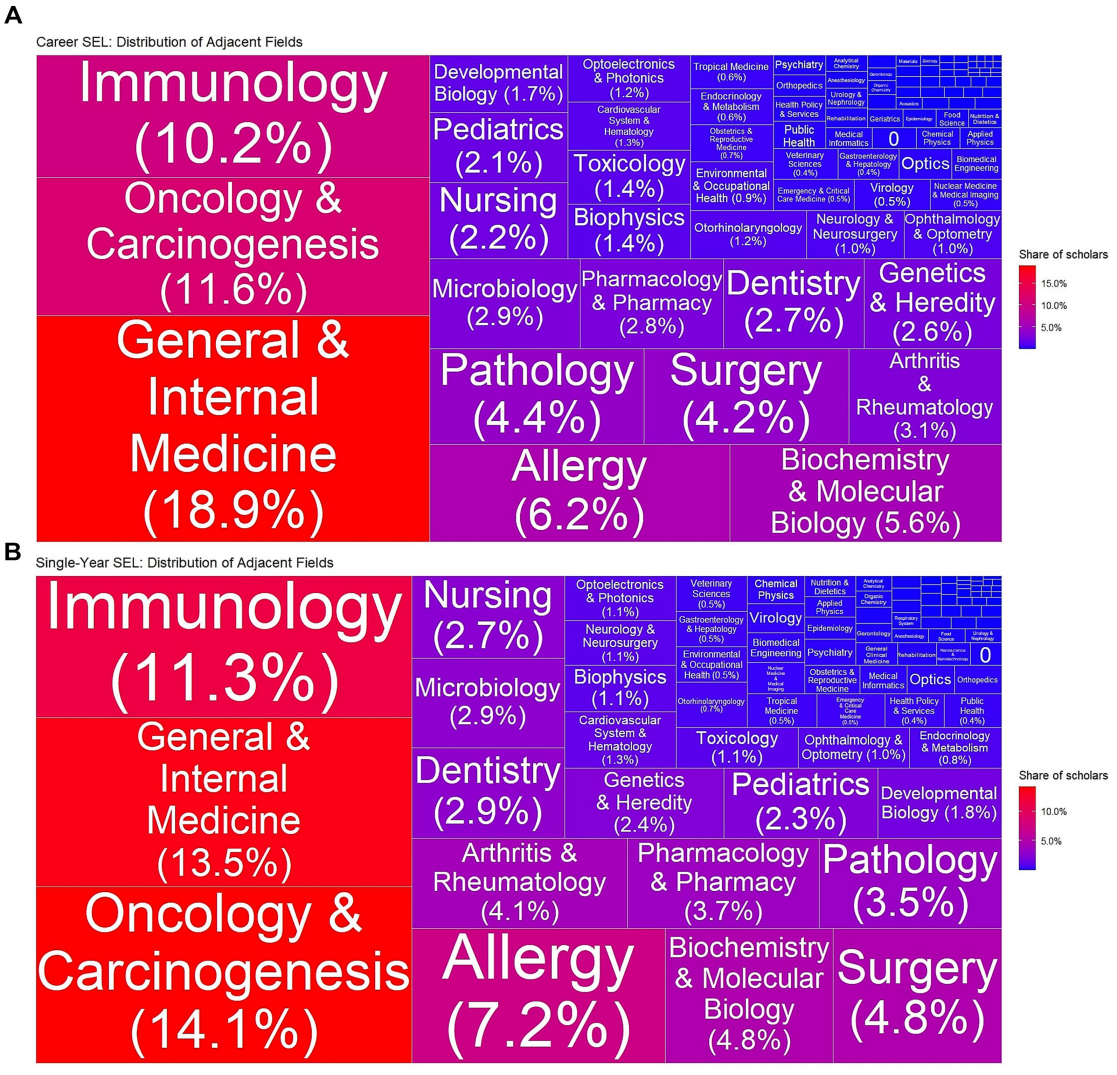
Treemap chart of adjacent sub-fields of excellent dermatologic scholars (EDS) in the Stanford–Elsevier Top 2% lists (2017–2023): **(A)** Career-long and **(B)** single-year lists.

For core bibliometric outcomes, academic age was most strongly associated with the modified *H*-index in the *career-long* SEL (*ρ* = 0.312) and with the composite score in the *single-year* SEL (*ρ* = 0.145). Negative correlations were observed with self-citations (*ρ* = −0.195 and −0.184) (Figure 4).

Stronger correlations with secondary bibliometric outcomes were again observed in the *single-year* SEL compared with the *career-long* SEL, for instance, the number of single-authored papers (*ρ* = 0.546 vs. 0.357) and the number of single-, first-, and last-authored papers (*ρ* = 0.556 vs. 0.312) (Supplementary Table S8).

### 3.5 Disciplinary classifications

The disciplinary classification of *Dermatology & Venereal Diseases* was recorded as the primary subfield in 72.8% of EDS records and as the secondary subfield in 27.2%. The adjacent subfields are shown in Figure 4.

### 3.6 Time-trend analyses of dermatologic research excellence

Between 2017 and 2023, the share of EDS from high-income countries declined slightly (−1.69% and −4.74% in the *career-long* and *single-year* SEL, respectively). Regionally, the share of AMRO decreased (−3.13% and −3.83%), while other WHO regions increased, such as WPRO (+3.03% and +3.61%) (Table 1). Female representation rose from 20.0% to 24.9% in the *career-long* SEL and from 22.9 to 30.2% in the *single-year* SEL (Table 6).

### 3.7 Determinants of female representation among excellent dermatologic scholars

Female group membership was associated with a younger academic age (*career-long* OR = 0.959; s*ingle-year* OR = 0.966). Compared with AMRO, women were less likely to be represented in SEARO (OR = 0.653; 0.535) and WPRO (OR = 0.872; 0.776). Representation was higher in non-English-speaking countries than in English-speaking ones (OR = 1.197; 1.048). At the national level, the human development index was negatively associated with female membership (OR = 0.973; 0.957). In terms of budgetary policies, GDP spent on research and development was positively associated with female membership (OR = 1.379; 1.366), while GDP spent on health was negatively associated (OR = 0.959; 0.966) (Table 7).

**Table 7:** Individual-level analysis: logistic regression models for female gender (group membership) among dermatologic scholars in the Stanford–Elsevier Lists (SEL) of top scientists worldwide (2017–2023).

Group	Predictor	Career-long SEL		Single-year SEL	
		OR (CI 95%)	*p*	OR (CI 95%)	*p*
Individual	Academic age (*per year*)	0.959 (0.953–0.964)	**<0.001**	0.966 (0.961–0.971)	**<0.001**
World Bank	World Bank: low income *vs*. high income	0.000 (0.000–Inf)	0.929	0.404 (0.224–0.727)	**0.003**
	World Bank: lower-middle income *vs*. high income	0.719 (0.457–1.132)	0.155	1.278 (0.985–1.657)	0.065
	World Bank: upper-middle income *vs*. high income	3.193 (0.797–12.793)	0.101	5.231 (1.837–14.895)	**0.002**
WHO region	WHO region: AFRO *vs*. AMRO	0.000 (0.000–Inf)	0.957	0.570 (0.332–0.978)	**0.041**
	WHO region: EMRO *vs*. AMRO	0.987 (0.884–1.103)	0.820	0.891 (0.797–0.996)	**0.042**
	WHO region: EURO *vs*. AMRO	0.000 (0.000–Inf)	0.958	0.176 (0.070–0.438)	**<0.001**
	WHO region: SEARO *vs*. AMRO	0.653 (0.528–0.808)	**<0.001**	0.535 (0.441–0.648)	**<0.001**
	WHO region: WPRO *vs*. AMRO	0.872 (0.783–0.970)	**0.012**	0.776 (0.699–0.861)	**<0.001**
Language	English-speaking: no *vs*. yes	1.197 (1.071–1.338)	**0.001**	1.048 (1.015–1.082)	**0.004**
Health system	Universal health coverage index (UHC)	0.996 (0.981–1.010)	0.546	1.003 (0.991–1.015)	0.674
	General government expenditure on health (%)	1.057 (0.504–2.217)	0.882	1.852 (1.000–3.429)	0.050
Gender equity	Gender inequality index (GII)	1.000 (0.996–1.005)	0.888	1.001 (0.998–1.004)	0.407
	Maternal mortality per 100 K live births	1.006 (0.998–1.014)	0.153	1.013 (1.007–1.020)	**<0.001**
	Adolescent birth rate (*per* 1 K women aged 15–19)	0.968 (0.942–0.995)	**0.022**	0.988 (0.971–1.006)	0.193
	Education gap 25+ (male−female)	0.937 (0.919–0.955)	**<0.001**	0.978 (0.968–0.987)	**<0.001**
	Employment gap 15+ (male−female)	1.016 (1.009–1.024)	**<0.001**	1.023 (1.015–1.030)	**<0.001**
	Female share of parliamentary seats (%)	4.060 (0.552–29.843)	0.169	1.753 (0.496–6.192)	0.384
Human development	Human development index (HDI)	0.973 (0.949–0.999)	**0.039**	0.957 (0.937–0.978)	**<0.001**
	Life expectancy at birth (years)	1.024 (0.974–1.076)	0.355	1.020 (0.983–1.058)	0.288
	Expected years of schooling	1.065 (1.021–1.111)	**0.004**	1.088 (1.046–1.132)	**<0.001**
	Mean years of schooling	1.000 (1.000–1.000)	0.463	1.000 (1.000–1.000)	0.391
	Gross national income per capita (USD)	1.024 (0.955–1.098)	0.501	0.982 (0.928–1.039)	0.534
Budgetary policies	GDP spent on research & development (*per %*)	1.379 (1.282–1.483)	**<0.001**	1.366 (1.273–1.467)	**<0.001**
	GDP spent on education (*per %*)	1.014 (0.997–1.031)	0.114	1.028 (1.012–1.044)	**<0.001**
	GDP spent on health (*per %*)	0.959 (0.953–0.964)	**<0.001**	0.966 (0.961–0.971)	**<0.001**

Bold font refers to statistically significant associations (*p* < 0.05).

### 3.8 Effects of academic age on bibliometric outcomes

Regression findings highlighted the consistent role of academic age in shaping bibliometric outcomes. Each extra year of age was associated with higher citation counts (*career-long β* = 84.1; *single-year β* = 2.6), higher modified *H*-index values (*β* = 0.208; 0.024), and increased composite scores (*β* = 0.005; 0.004). These associations were similar in male and female EDS. The secondary bibliometric outcomes followed the same favourable direction (Figure 3; Table 8).

**Table 8:** Individual-level analyses: linear regression models of scholarly output metrics based on academic age “predictor” of dermatologic scholars in the Stanford–Elsevier Lists (2017–2023).

Scholarly output metric	Overall: *β* (95% CI); *p*	Female: *β* (95% CI); *p*	Male: *β* (95% CI); *p*
Career-long SEL			
Total citations^╪^	84.065 (66.365 to 101.766); **<0.001**	116.001 (74.089 to 157.914); **<0.001**	67.433 (44.595 to 90.271); **<0.001**
Modified *H*-index^╪^	0.208 (0.194 to 0.222); **<0.001**	0.245 (0.213 to 0.278); **<0.001**	0.187 (0.170 to 0.205); **<0.001**
Composite score^╪^	0.005 (0.005 to 0.006); **<0.001**	0.008 (0.006 to 0.009); **<0.001**	0.005 (0.004 to 0.005); **<0.001**
Self-citations (%)	−0.001 (−0.001 to −0.001); **<0.001**	−0.001 (−0.001 to −0.001); **<0.001**	-0.001 (−0.001 to −0.001); **<0.001**
Total papers	4.089 (3.717 to 4.462); **<0.001**	3.679 (2.942 to 4.417); **<0.001**	3.409 (2.940 to 3.878); **<0.001**
Single-authored papers (number)	0.771 (0.713 to 0.830); **<0.001**	0.362 (0.244 to 0.481); **<0.001**	0.739 (0.664 to 0.815); **<0.001**
Single-authored papers (citations)^╪^	7.608 (6.754 to 8.461); **<0.001**	7.202 (5.200 to 9.204); **<0.001**	8.211 (7.126 to 9.295); **<0.001**
Single- and first-authored papers (number)	1.242 (1.130 to 1.354); **<0.001**	0.784 (0.562 to 1.006); **<0.001**	1.146 (1.001 to 1.292); **<0.001**
Single- and first-authored papers (citations)^╪^	−5.340 (−8.367 to −2.312); **<0.001**	−1.423 (−9.793 to 6.947); 0.739	−5.492 (−9.245 to −1.739); **0.004**
Single-, first-, and last-authored papers (number)	3.549 (3.313 to 3.785); **<0.001**	2.288 (1.829 to 2.747); **<0.001**	3.302 (2.996 to 3.608); **<0.001**
Single-, first-, and last-authored papers (citations)^╪^	57.184 (49.748 to 64.620); **<0.001**	51.120 (34.344 to 67.896); **<0.001**	54.752 (45.103 to 64.402); **<0.001**
Single-year SEL			
Total citations^╪^	2.631 (0.001 to 5.260); 0.050	2.658 (−2.552 to 7.868); 0.318	3.588 (0.091 to 7.085); **0.044**
Modified *H*-index^╪^	0.024 (0.020 to 0.028); **<0.001**	0.028 (0.020 to 0.035); **<0.001**	0.023 (0.018 to 0.028); **<0.001**
Composite score^╪^	0.004 (0.003 to 0.005); **<0.001**	0.005 (0.003 to 0.006); **<0.001**	0.004 (0.003 to 0.005); **<0.001**
Self-citations (%)	−0.001 (−0.002 to −0.001); **<0.001**	−0.001 (−0.001 to −0.001); **<0.001**	-0.001 (−0.002 to −0.001); **<0.001**
Total papers	7.262 (6.927 to 7.597); **<0.001**	6.163 (5.622 to 6.705); **<0.001**	6.953 (6.513 to 7.394); **<0.001**
Single-authored papers (number)	0.822 (0.775 to 0.869); **<0.001**	0.554 (0.471 to 0.638); **<0.001**	0.843 (0.780 to 0.906); **<0.001**
Single-authored papers (citations)^╪^	0.464 (0.392 to 0.535); **<0.001**	0.404 (0.277 to 0.532); **<0.001**	0.509 (0.409 to 0.608); **<0.001**
Single- and first-authored papers (number)	1.607 (1.509 to 1.705); **<0.001**	1.421 (1.261 to 1.581); **<0.001**	1.546 (1.412 to 1.679); **<0.001**
Single- and first-authored papers (citations)^╪^	−1.279 (−1.593 to −0.966); **<0.001**	−0.895 (−1.697 to −0.094); **0.029**	−1.196 (−1.575 to −0.817); **<0.001**
Single-, first-, and last-authored papers (number)	4.837 (4.627 to 5.047); **<0.001**	3.834 (3.493 to 4.174); **<0.001**	4.791 (4.507 to 5.074); **<0.001**
Single-, first-, and last-authored papers (citations)^╪^	2.778 (2.018 to 3.538); **<0.001**	3.072 (1.499 to 4.645); **<0.001**	2.977 (1.945 to 4.009); **<0.001**

^╪^Self-citations were excluded. Bold font refers to statistically significant associations (*p* < 0.05).

### 3.9 Regression analyses of dermatologic research excellence

Two regression approaches were pursued to identify determinants of research excellence. The first approach evaluated each national-level determinant separately, with adjustment for gender and academic age. The second approach combined both individual-level variables and grouped national-level indicators (World Bank income category, WHO region, language, GII, HDI, and UHC) into one comprehensive model.

In the first approach, economic status showed clear associations: scholars from lower-middle- (adj. *β* = −5137; −487) and upper-middle-income countries (adj. *β* = −2514; −206) had fewer citations than those from high-income settings. Conversely, non-English-speaking countries (adj. *β* = 1377; 76), stronger UHC performance (adj. *β* = 211; 17), and higher HDI (adj. *β* = 16,845; 1488) were linked to higher citation counts (Table 9).

**Table 9:** Individual-level analysis: linear regression of scholarly outputs of dermatologic scholars in the Stanford–Elsevier Lists (SEL) of top scientists worldwide (2017–2023).

		Career-long SEL							
		Citations count		Modified *H*-index		Composite score (C)		% Self-citations	
Individual-level determinants		*β* (95% CI)	*p*	*β* (95% CI)	*p*	*β* (95% CI)	*p*	*β* (95% CI)	*p*
	Gender (male vs. female)	1191 (726 to 1657)	**<0.001**	2.164 (1.790 to 2.537)	**<0.001**	0.077 (0.062 to 0.093)	**<0.001**	−0.011 (−0.015 to −0.008)	**<0.001**
	Academic age (*per* year)	84 (66 to 102)	**<0.001**	0.208 (0.194 to 0.222)	**<0.001**	0.005 (0.005 to 0.006)	**<0.001**	−0.001 (−0.001 to −0.001)	**<0.001**
National-level determinants		Adj. *β* (95% CI)	*p*	Adj. *β* (95% CI)	*p*	Adj. *β* (95% CI)	*p*	Adj. *β* (95% CI)	*p*
WB	Lower-middle income *vs*. high income	−5137 (−8066 to −2209)	**<0.001**	−1.213 (−3.475 to 1.048)	0.293	−0.078 (−0.172 to 0.017)	0.109	−0.023 (−0.044 to −0.002)	**0.035**
	Upper-middle income *vs*. High income	−2514 (−4051 to −977)	**0.001**	−1.961 (−3.148 to −0.774)	**0.001**	−0.172 (−0.221 to −0.122)	**<0.001**	0.013 (0.002 to 0.024)	**0.022**
WHO region	EURO *vs*. AMRO	1868 (1454 to 2283)	**<0.001**	0.106 (−0.215 to 0.427)	0.518	0.001 (−0.012 to 0.015)	0.878	0.045 (0.042 to 0.048)	**<0.001**
	WPRO *vs*. AMRO	−156 (−872 to 561)	0.670	−0.313 (−0.869 to 0.243)	0.269	−0.047 (−0.070 to −0.024)	**<0.001**	0.042 (0.037 to 0.047)	**<0.001**
	EMRO *vs*. AMRO	−1991 (−5203 to 1221)	0.224	−0.149 (−2.640 to 2.341)	0.906	−0.082 (−0.187 to 0.022)	0.123	−0.003 (−0.025 to 0.019)	0.803
	AFRO *vs*. AMRO	22150 (16084 to 28215)	**<0.001**	19.747 (15.043 to 24.450)	**<0.001**	0.509 (0.311 to 0.707)	**<0.001**	0.036 (−0.006 to 0.077)	0.090
	SEARO *vs*. AMRO	−4067 (−7320 to −815)	**0.014**	−2.115 (−4.638 to 0.407)	0.100	−0.196 (−0.303 to −0.090)	**<0.001**	0.005 (−0.018 to 0.027)	0.676
L	English-speaking: no *vs*. yes	1377 (982 to 1771)	**<0.001**	−0.115 (−0.422 to 0.192)	0.463	−0.021 (−0.034 to −0.008)	**0.002**	0.046 (0.043 to 0.048)	**<0.001**
HS	Universal health coverage index (UHC)	211 (40 to 381)	**0.016**	0.030 (−0.102 to 0.162)	0.654	0.006 (0.001 to 0.012)	**0.030**	0.002 (0.000 to 0.003)	**0.009**
	General government expenditure on health (%)	−54.629 (−108.865 to −0.393)	**0.048**	0.075 (0.033 to 0.117)	**<0.001**	0.005 (0.003 to 0.007)	**<0.001**	−0.003 (−0.003 to −0.003)	**<0.001**
Gender equity	Gender inequality index (*per n*)	−11203 (−13948 to −8457)	**<0.001**	−1.611 (−3.738 to 0.516)	0.138	−0.084 (−0.173 to 0.006)	0.066	−0.257 (−0.276 to −0.238)	**<0.001**
	Maternal mortality rate (*per n*)	−39 (−55 to −23)	**<0.001**	−0.005 (−0.017 to 0.007)	0.410	−0.001 (−0.001 to 0.000)	0.056	−0.001 (−0.001 to −0.001)	**<0.001**
	Adolescent birth rate (*per n*)	−103 (−135 to −72)	**<0.001**	−0.017 (−0.042 to 0.007)	0.169	−0.001 (−0.002 to 0.000)	0.056	−0.002 (−0.003 to −0.002)	**<0.001**
	Employment Gap (Male − Female) (*per %*)	−112 (−160 to −64)	**<0.001**	−0.033 (−0.070 to 0.005)	0.086	−0.004 (−0.006 to −0.003)	**<0.001**	1.649e-04 (−1.834e-04 to 5.132e-04)	0.353
	Education gap (male − female) (*per %*)	−45 (−140 to 49)	0.346	−0.131 (−0.203 to −0.058)	**<0.001**	−0.007 (−0.010 to −0.004)	**<0.001**	0.002 (0.002 to 0.003)	**<0.001**
	Female share of parliamentary seats (*per %*)	71 (43 to 98)	**<0.001**	0.008 (−0.013 to 0.029)	0.463	0.001 (0.000 to 0.002)	**0.046**	0.001 (0.001 to 0.002)	**<0.001**
Human development	Human development index (*per n*)	16845 (9870 to 23820)	**<0.001**	12.746 (7.363 to 18.130)	**<0.001**	0.831 (0.605 to 1.057)	**<0.001**	0.092 (0.042 to 0.142)	**<0.001**
	Gross national income per capita (*per USD*)	0.004 (−0.012 to 0.020)	0.633	2.033e-05 (8.169e-06 to 3.248e-05)	**0.001**	1.757e-06 (1.247e-06 to 2.267e-06)	**<0.001**	−9.344e-07 (−1.046e-06 to −8.225e-07)	**<0.001**
	Expected years of schooling (*per year*)	−15 (−175 to 145)	0.855	0.013 (−0.111 to 0.136)	0.838	−0.002 (−0.007 to 0.003)	0.386	0.009 (0.008 to 0.010)	**<0.001**
	Mean Years of Schooling (*per year*)	303 (122 to 485)	**0.001**	0.516 (0.376 to 0.656)	**<0.001**	0.030 (0.024 to 0.035)	**<0.001**	−0.006 (−0.008 to −0.005)	**<0.001**
	Life expectancy at birth (*per year*)	217 (121 to 313)	**<0.001**	−0.074 (−0.147 to 0.000)	0.051	−0.004 (−0.007 to −0.001)	**0.015**	0.008 (0.007 to 0.008)	**<0.001**
Budget. policies	GDP spent on research (*per %*)	−318 (−575 to −60)	**0.016**	0.131 (−0.068 to 0.330)	0.197	0.015 (0.007 to 0.024)	**<0.001**	−0.013 (−0.015 to −0.012)	**<0.001**
	GDP spent on education (*per %*)	−756 (−1017 to −495)	**<0.001**	−0.355 (−0.557 to −0.153)	**<0.001**	−0.003 (−0.011 to 0.006)	0.502	−0.014 (−0.015 to −0.012)	**<0.001**
	GDP spent on health (*per %*)	−60 (−123 to 2)	0.059	0.043 (−0.005 to 0.091)	0.078	0.006 (0.003 to 0.008)	**<0.001**	−0.006 (−0.006 to −0.006)	**<0.001**
Disease burden	DALYs attributed to acne vulgaris	64 (51 to 78)	**<0.001**	0.010 (−0.001 to 0.021)	0.072	0.001 (0.000 to 0.001)	**0.005**	0.001 (0.001 to 0.001)	**<0.001**
	DALYs attributed to dermatitis	8 (−2 to 17)	0.122	−0.009 (−0.017 to −0.002)	**0.017**	5.642e-04 (2.486e-04 to 8.799e-04)	**<0.001**	−6.202e-04 (−6.891e-04 to −5.514e-04)	**<0.001**
	DALYs attributed to psoriasis	39 (33 to 45)	**<0.001**	0.014 (0.009 to 0.018)	**<0.001**	7.898e-04 (6.006e-04 to 9.790e-04)	**<0.001**	3.390e-04 (2.974e-04 to 3.805e-04)	**<0.001**
	DALYs attributed to scabies	−38 (−51 to −24)	**<0.001**	−0.021 (−0.031 to −0.010)	**<0.001**	−0.002 (−0.002 to −0.001)	**<0.001**	1.158e-04 (1.762e-05 to 2.140e-04)	**0.021**
	DALYs attributed to fungal skin infections	39 (30 to 49)	**<0.001**	0.002 (−0.005 to 0.009)	0.617	−2.206e-04 (−5.307e-04 to 8.944e-05)	0.163	0.001 (0.001 to 0.001)	**<0.001**
	DALYs attributed to viral skin infections	−1 (−9 to 7)	0.781	0.012 (0.006 to 0.017)	**<0.001**	6.111e-04 (3.638e-04 to 8.585e-04)	**<0.001**	−3.278e-04 (−3.824e-04 to −2.732e-04)	**<0.001**

		*Single-year* SEL							
		Citations count		Modified *H*-index		Composite score (C)		% Self-citations	
Individual-level determinants		*β* (95% CI)	*p*	*β* (95% CI)	*p*	*β* (95% CI)	*p*	*β* (95% CI)	*p*
	Gender (male vs. female)	90 (22 to 157)	**0.009**	0.300 (0.204 to 0.396)	**<0.001**	0.060 (0.043 to 0.077)	**<0.001**	−0.008 (−0.012 to −0.004)	**<0.001**
	Academic age (*per* year)	3 (0 to 5)	0.050	0.024 (0.020 to 0.028)	**<0.001**	0.004 (0.003 to 0.005)	**<0.001**	−0.001 (−0.002 to −0.001)	**<0.001**
National-level determinants		Adj. *β* (95% CI)	*p*	Adj. *β* (95% CI)	*p*	Adj. *β* (95% CI)	*p*	Adj. *β* (95% CI)	*p*
WB	Lower-middle income *vs*. high income	−487 (−765 to −209)	**<0.001**	−0.443 (−0.832 to −0.053)	**0.026**	−0.148 (−0.216 to −0.079)	**<0.001**	−0.016 (−0.031 to −0.000)	**0.043**
	Upper-middle income *vs*. high income	−206 (−374 to −38)	**0.016**	0.052 (−0.183 to 0.288)	0.662	−0.111 (−0.152 to −0.070)	**<0.001**	0.003 (−0.006 to 0.012)	0.532
WHO region	EURO *vs*. AMRO	160 (89 to 231)	**<0.001**	−0.053 (−0.151 to 0.045)	0.291	−0.014 (−0.031 to 0.004)	0.125	0.041 (0.037 to 0.045)	**<0.001**
	WPRO *vs*. AMRO	−157 (−266 to −47)	**0.005**	−0.241 (−0.392 to −0.089)	0.002	−0.082 (−0.108 to −0.055)	**<0.001**	0.018 (0.012 to 0.023)	**<0.001**
	EMRO *vs*. AMRO	−353 (−652 to −54)	**0.021**	−0.163 (−0.577 to 0.250)	0.439	−0.127 (−0.200 to −0.054)	**<0.001**	0.031 (0.015 to 0.047)	**<0.001**
	AFRO *vs*. AMRO	2568 (1918 to 3219)	**<0.001**	6.844 (5.943 to 7.744)	**<0.001**	0.629 (0.469 to 0.789)	**<0.001**	0.009 (−0.026 to 0.044)	0.627
	SEARO *vs*. AMRO	−440 (−764 to −116)	**0.008**	−0.803 (−1.252 to −0.355)	**<0.001**	−0.225 (−0.305 to −0.146)	**<0.001**	0.014 (−0.004 to 0.031)	0.121
L	English-speaking: no *vs*. yes	76 (10 to 142)	**0.024**	−0.031 (−0.123 to 0.062)	0.517	−0.037 (−0.053 to −0.020)	**<0.001**	0.038 (0.035 to 0.042)	**<0.001**
HS	Universal health coverage index (UHC)	17 (1 to 33)	**0.037**	0.003 (−0.019 to 0.025)	0.791	0.007 (0.003 to 0.011)	**<0.001**	0.001 (−0.000 to 0.001)	0.237
	General government expenditure on health (%)	5 (−2 to 13)	0.167	0.012 (0.001 to 0.022)	**0.027**	0.007 (0.005 to 0.008)	**<0.001**	−0.002 (−0.003 to −0.002)	**<0.001**
Gender equity	Gender inequality index (*per n*)	−830 (−1218 to −441)	**<0.001**	0.181 (−0.362 to 0.725)	0.513	−0.094 (−0.190 to 0.001)	0.053	−0.135 (−0.156 to −0.114)	**<0.001**
	Maternal mortality rate (*per n*)	−0.606 (−2.509 to 1.298)	0.533	0.003 (0.001 to 0.006)	**0.014**	−2.355e-04 (−7.037e-04 to 2.328e-04)	0.324	−4.138e-04 (−5.185e-04 to −3.090e-04)	**<0.001**
	Adolescent birth rate (*per n*)	−4.037 (−8.301 to 0.227)	0.064	0.009 (0.003 to 0.015)	**0.003**	−0.000 (−0.001 to 0.001)	0.997	−0.001 (−0.001 to −0.001)	**<0.001**
	Employment gap (male−female) (*per %*)	−16 (−21 to −10)	**<0.001**	−0.013 (−0.020 to −0.006)	**<0.001**	−0.005 (−0.006 to −0.004)	**<0.001**	3.064e-04 (1.746e-05 to 5.954e-04)	**0.038**
	Education gap (male−female) (*per %*)	−18 (−29 to −7)	**0.001**	−0.035 (−0.050 to −0.019)	**<0.001**	−0.010 (−0.013 to −0.008)	**<0.001**	0.001 (0.001 to 0.002)	**<0.001**
	Female share of parliamentary seats (*per %*)	13 (8 to 17)	**<0.001**	0.011 (0.005 to 0.017)	**<0.001**	0.003 (0.002 to 0.004)	**<0.001**	0.001 (0.001 to 0.001)	**<0.001**
Human development	Human development index (*per n*)	1488 (721 to 2256)	**<0.001**	0.905 (−0.168 to 1.979)	0.098	0.666 (0.478 to 0.854)	**<0.001**	−0.003 (−0.046 to 0.039)	0.876
	Gross national income per capita (*per USD*)	0.003 (0.001 to 0.005)	**0.005**	6.166e-06 (3.134e-06 to 9.198e-06)	**<0.001**	2.311e-06 (1.780e-06 to 2.842e-06)	**<0.001**	−6.256e-07 (−7.446e-07 to −5.065e-07)	**<0.001**
	Expected years of schooling (*per year*)	0.864 (−23.919 to 25.647)	0.946	−0.010 (−0.045 to 0.025)	0.572	−0.002 (−0.008 to 0.004)	0.607	0.005 (0.004 to 0.006)	**<0.001**
	Mean years of schooling (*per year*)	50 (27 to 72)	**<0.001**	0.092 (0.060 to 0.123)	**<0.001**	0.030 (0.025 to 0.036)	**<0.001**	−0.004 (−0.005 to −0.003)	**<0.001**
	Life expectancy at birth (*per year*)	8 (−5 to 21)	0.248	−0.052 (−0.071 to −0.034)	**<0.001**	−0.005 (−0.008 to −0.002)	**0.003**	0.004 (0.004 to 0.005)	**<0.001**
Budget. policies	GDP spent on research (*per %*)	−1 (−36 to 34)	0.953	0.009 (−0.040 to 0.058)	0.722	0.017 (0.009 to 0.026)	**<0.001**	−0.013 (−0.015 to −0.011)	**<0.001**
	GDP spent on education (*per %*)	22 (−20 to 65)	0.305	0.050 (−0.009 to 0.110)	0.097	0.017 (0.007 to 0.027)	**0.001**	−0.012 (−0.014 to −0.009)	**<0.001**
	GDP spent on health (*per %*)	9.410 (−0.270 to 19.090)	0.057	0.018 (0.004 to 0.031)	**0.010**	0.009 (0.007 to 0.012)	**<0.001**	−0.004 (−0.005 to −0.004)	**<0.001**
Disease burden	DALYs attributed to acne vulgaris	7 (5 to 10)	**<0.001**	0.002 (−0.001 to 0.005)	0.277	0.001 (0.000 to 0.001)	**0.025**	8.349e-04 (7.097e-04 to 9.601e-04)	**<0.001**
	DALYs attributed to dermatitis	3 (2 to 4)	**<0.001**	−0.002 (−0.004 to 0.000)	0.101	0.001 (0.001 to 0.001)	**<0.001**	−3.779e-04 (−4.564e-04 to −2.995e-04)	**<0.001**
	DALYs attributed to psoriasis	4 (3 to 5)	**<0.001**	0.003 (0.002 to 0.004)	**<0.001**	0.001 (0.001 to 0.001)	**<0.001**	2.690e-04 (2.209e-04 to 3.170e-04)	**<0.001**
	DALYs attributed to scabies	−4 (−5 to −2)	**<0.001**	−0.003 (−0.005 to −0.000)	**0.017**	−0.001 (−0.002 to −0.001)	**<0.001**	−5.495e-05 (−1.373e-04 to 2.737e-05)	0.191
	DALYs attributed to fungal skin infections	3 (2 to 5)	**<0.001**	−0.001 (−0.003 to 0.001)	0.403	−4.682e-04 (−8.647e-04 to −7.171e-05)	**0.021**	0.001 (0.001 to 0.001)	**<0.001**
	DALYs attributed to viral skin infections	0.914 (−0.269 to 2.097)	0.130	0.003 (0.002 to 0.005)	**<0.001**	0.001 (0.001 to 0.001)	**<0.001**	−2.893e-04 (−3.543e-04 to −2.242e-04)	**<0.001**

Each national-level determinant is adjusted (Adj. *β*) for gender and academic age. Bold font refers to statistically significant associations (*p* < 0.05).

In the second approach, male gender (adj. *β* = 1004; 107) and academic age (*career-long* adj. *β* = 83) were significant positive predictors, while being affiliated with English-speaking countries showed a negative association (*career-long* adj. *β* = −1564). Comparable patterns were confirmed when the modified *H*-index and composite score were used as outcomes (Table 10).

**Table 10:** Individual-level analysis: multivariable regression models of scholarly outputs of dermatologic scholars in the Stanford–Elsevier Lists (SEL) of top scientists worldwide (2017–2023).

Career-long SEL	Citations count		Modified H-index		Composite score (C)		% Self-citations	
	*R*^2^ = 0.034		*R*^2^ = 0.107		*R*^2^ = 0.061		*R*^2^ = 0.188	
	Adj. *β* (95% CI)	*p*	Adj. *β* (95% CI)	*p*	Adj. *β* (95% CI)	*p*	Adj. *β* (95% CI)	*p*
Gender (male vs. female)	1004 (530 to 1477)	**<0.001**	1.473 (1.106 to 1.839)	**<0.001**	0.061 (0.046 to 0.077)	**<0.001**	−0.009 (−0.013 to −0.006)	**<0.001**
Academic age (*per* year)	83 (62 to 103)	**<0.001**	0.202 (0.186 to 0.218)	**<0.001**	0.005 (0.004 to 0.006)	**<0.001**	−0.001 (−0.001 to −0.001)	**<0.001**
World Bank (lower-middle *vs*. high income)	−3559 (−10621 to 3503)	0.323	5.235 (−0.236 to 10.71)	0.061	0.259 (0.029 to 0.489)	**0.027**	0.074 (0.026 to 0.121)	**0.002**
World Bank (upper-middle *vs*. high income)	−4880 (−7737 to −2023)	**<0.001**	0.064 (−2.149 to 2.278)	0.955	−0.090 (−0.183 to 0.003)	0.058	0.018 (−0.001 to 0.038)	0.060
WHO region (AFRO *vs*. AMRO)	27469 (20418 to 34521)	**<0.001**	26.12 (20.66 to 31.587)	**<0.001**	0.752 (0.522 to 0.981)	**<0.001**	0.055 (0.007 to 0.102)	**0.024**
WHO region (EMRO *vs*. AMRO)	480 (−3677 to 4637)	0.821	1.939 (−1.281 to 5.160)	0.238	−0.072 (−0.208 to 0.063)	0.296	−0.018 (−0.046 to 0.010)	0.206
WHO region (EURO *vs*. AMRO)	1375 (402 to 2347)	**0.006**	0.031 (−0.723 to 0.784)	0.937	0.014 (−0.018 to 0.045)	0.393	0.023 (0.016 to 0.030)	**<0.001**
WHO region (SEARO *vs*. AMRO)	1745 (−4260 to 7750)	0.569	2.096 (−2.556 to 6.749)	0.377	−0.212 (−0.408 to −0.017)	0.033	0.016 (−0.025 to 0.056)	0.446
WHO region (WPRO *vs*. AMRO)	−354 (−1476 to 768)	0.537	0.045 (−0.825 to 0.914)	0.920	−0.019 (−0.055 to 0.018)	0.320	0.025 (0.017 to 0.032)	**<0.001**
English-speaking (yes *vs*. no)	−1564 (−2260 to −868)	**<0.001**	−0.391 (−0.93 to 0.148)	0.155	0.009 (−0.014 to 0.032)	0.432	−0.038 (−0.042 to −0.033)	**<0.001**
Gender Inequality index (*per n*)	6746 (−1699 to 15191)	0.117	4.64 (−1.902 to 11.183)	0.164	0.150 (−0.125 to 0.425)	0.285	0.066 (0.010 to 0.123)	**0.022**
Human development Index (*per n*)	5861 (−9642 to 21364)	0.459	32.15 (20.14 to 44.16)	**<0.001**	1.004 (0.500 to 1.509)	**<0.001**	0.168 (0.064 to 0.272)	**0.002**
Universal health coverage index (UHC)	268 (−161 to 697)	0.221	0.180 (−0.153 to 0.512)	0.289	−0.002 (−0.016 to 0.012)	0.798	0.006 (0.003 to 0.009)	**<0.001**

Single-year SEL	Citations count		Modified H-index		Composite score (C)		% Self-citations	
	*R*^2^ = 0.022		*R*^2^ = 0.068		*R*^2^ = 0.054		*R*^2^ = 0.126	
	Adj. *β* (95% CI)	*p*	Adj. *β* (95% CI)	*p*	Adj. *β* (95% CI)	*p*	Adj. *β* (95% CI)	*p*
Gender (male vs. female)	107 (34 to 181)	**0.004**	0.265 (0.163 to 0.366)	**<0.001**	0.049 (0.031 to 0.067)	**<0.001**	−0.004 (−0.008 to −0.001)	**0.025**
Academic age (per year)	2 (−1 to 5)	0.159	0.023 (0.019 to 0.027)	**<0.001**	0.004 (0.003 to 0.004)	**<0.001**	-0.001 (−0.001 to −0.001)	**<0.001**
World Bank (lower-middle *vs*. high income)	−83 (−836 to 670)	0.829	1.579 (0.539 to 2.620)	**0.003**	0.166 (−0.019 to 0.35)	0.079	−0.081 (−0.121 to −0.041)	**<0.001**
World Bank (upper-middle *vs*. high income)	−228 (−579 to 123)	0.203	0.377 (−0.11 to 0.862)	0.128	−0.03 (−0.116 to 0.056)	0.501	−0.045 (−0.064 to −0.026)	**<0.001**
WHO region (AFRO *vs*. AMRO)	3020 (2238 to 3803)	**<0.001**	8.152 (7.072 to 9.233)	**<0.001**	0.824 (0.632 to 1.015)	**<0.001**	−0.004 (−0.045 to 0.038)	0.867
WHO region (EMRO *vs*. AMRO)	−114 (−527 to 299)	0.589	0.265 (−0.306 to 0.84)	0.362	−0.066 (−0.167 to 0.035)	0.200	0.027 (0.005 to 0.049)	**0.016**
WHO region (EURO *vs*. AMRO)	94 (−63 to 251)	0.241	−0.23 (−0.45 to −0.02)	**0.036**	−0.019 (−0.057 to 0.02)	0.339	0.028 (0.019 to 0.036)	**<0.001**
WHO region (SEARO *vs*. AMRO)	−2 (−654 to 649)	0.994	0.050 (−0.850 to 0.95)	0.914	−0.158 (−0.317 to 0.002)	0.053	0.037 (0.002 to 0.071)	**0.039**
WHO region (WPRO *vs*. AMRO)	−183 (−359 to −8)	**0.041**	−0.34 (−0.59 to −0.099)	**0.006**	−0.067 (−0.11 to −0.024)	**0.002**	0.006 (−0.003 to 0.015)	0.217
English-speaking (yes *vs*. no)	−74 (−189 to 41)	0.205	−0.17 (−0.329 to −0.01)	**0.036**	0.00 (−0.028 to 0.028)	0.999	−0.027 (−0.033 to −0.021)	**<0.001**
Gender inequality index (*per n*)	40 (−1128 to 1209)	0.946	−0.066 (−1.68 to 1.55)	0.937	0.036 (−0.250 to 0.322)	0.805	0.056 (−0.006 to 0.119)	0.076
Human development index (*per n*)	697 (−1484 to 2879)	0.531	5.350 (2.337 to 8.363)	**<0.001**	0.85 (0.316 to 1.384)	**0.002**	−0.230 (−0.347 to −0.114)	**<0.001**
Universal health coverage index (UHC)	17 (−28 to 63)	0.455	0.077 (0.014 to 0.141)	**0.016**	0.002 (−0.010 to 0.013)	0.775	0.003 (0.000 to 0.005)	**0.041**

Bold font refers to statistically significant associations (*p* < 0.05).

## 4 Discussion

### 4.1 Key findings

The analysis revealed a highly disproportionate global distribution of EDS. Nearly all EDS were affiliated with high-income countries (97.9% in *career-long* and 94.5% in *single-year* SEL), with the EURO region contributing the largest regional share (47.5%–47.9%), while AFRO accounted for only 0.18%–0.32%. This imbalance translated into a pronounced high-income density advantage, with 0.671 EDS per 100,000 population in high-income settings compared with 0.002 in low-income ones. EDS counts correlated positively with the Human Development Index (HDI) and national investments in health, education, and research, and negatively with gender inequality, while most dermatologic DALYs (except scabies) showed positive correlations.

Institutional elitism was substantial, as the top 20 institutions collectively hosted 21.0% of *career-long* and 20.3% of *single-year* EDS, led by US universities and major German centers such as Charité – Berlin University of Medicine and Ludwig Maximilian University of Munich. Gender disparities remained evident, as women constituted only 22.9% of *career-long* and 28.6% of *single-year* EDS, while male scholars exhibited higher median citation counts (6860.5 vs. 6284.5) and significantly greater modified *H*-indices across both SELs. Academic age strongly predicted performance, correlating positively with modified *H*-index (*ρ* = 0.312) and composite score (*ρ* = 0.145), but inversely with self-citation percentage (*ρ* ≈ −0.19).

Multivariable analyses confirmed that each additional year of academic age increased citations (+84 career-long; +2.6 single-year) and composite metrics. Male gender remained a positive predictor, while greater UHC and higher HDI were associated with higher citation counts. Over 2017–2023, the dominance of high-income countries slightly declined, WPRO representation grew, and female participation gradually increased, suggesting a slow but measurable diversification of global dermatologic scholarship.

### 4.2 Disproportionate distribution of dermatologic scholarship

The disproportionate distribution of EDS appears both rooted in and reflective of enduring disparities in dermatologic research productivity across countries and regions. For instance, a bibliometric analysis of dermatology publications (1832–2019) based on Scopus, PubMed, WoS, and Embase revealed a pronounced concentration of research productivity in high-income countries, with the US (30.5%), Germany (8.1%), and the UK (8.1%) leading by volume, while Switzerland, Denmark, and Sweden ranked highest per capita; collectively, the top 10 countries accounted for over 75% of publications and citations (7). Consistently, a recent WoS-based analysis (1975–2024) focusing on chronic skin disease research demonstrated that the US (30.3%), Germany (10.4%), and the UK (9.5%) dominated dermatologic research output (8).

This pattern persisted during the COVID-19 pandemic, as a Scopus-based analysis (2020–2021) showed that the US (29.5%) and Italy (17.4%) produced nearly half of all publications in dermatology, reaffirming the dominance of high-income countries in the scientific response to the pandemic (46). In addition, disease-specific bibliometric analyses confirmed this high-income dominance, with the US leading research output on vitiligo (≈31.5%), pemphigus (27.3%), and psoriasis (22.0%), followed by other high-income nations such as Japan, Italy, Germany, and the UK (47–49).

Beyond disease-specific scholarship, dermatologic research in frontier domains shows that although the US and Western European countries continue to lead, China’s growing contribution indicates a gradual bipolarization of global dermatologic research between Western and East Asian hubs (27, 28, 50, 51). In WoS-based analyses, skin inflammation and regeneration research (1999–2022) was mainly produced by the US (28%), China (22.1%), and Germany (7.3%); skin microbiome studies (2013–2023) displayed a similar pattern; botulinum toxin research (2000–2023) was led by the US (32.9%), China (12.3%), and Germany (8.6%); and photodynamic therapy in dermatology (2000–2022) by the US (23.4%), Germany (14.6%), and China (13.3%) (27, 28, 50, 51).

In advanced biomedical fields, research output has become increasingly concentrated in East Asia, as shown by analyses of exosome studies in dermatology (2014–2023), where China accounted for 45%, the US for 14%, and South Korea for 8% of publications, and of extracellular vesicle applications in skin and plastic surgery (2003–2023), where China contributed 53.1%, the US 15.1%, and South Korea 6.2% (29, 30).

These geographic patterns should also be interpreted within the broader context of long-term national investment in science and technology. While the United States has historically maintained leadership in biomedical research expenditure, recent OECD and UNESCO data indicate comparatively faster growth in gross domestic expenditure on R&D in China and other East Asian economies over the past decade, potentially contributing to the evolving distribution of cumulative research impact (31–33).

### 4.3 Institutional elitism

The concentration of EDS within a limited number of leading universities can be attributed to the unequal distribution of dermatologic research productivity. For instance, a bibliometric analysis of clinical dermatology research (2005–2014) in Spain revealed a strong geographic concentration, with Barcelona and Madrid producing the highest research output, while Hospital Clínic (Barcelona) and the Instituto Valenciano de Oncología emerged as the most productive institutions (34). In Brazil, a WoS-based analysis (2012–2022) showed that universities in the state of São Paulo accounted for the largest proportion of affiliations (42.2%) in national dermatology research (35). Similarly, a WoS-based analysis (1980–2020) in Saudi Arabia revealed clear institutional elitism, with more than half of the country’s dermatologic research output originating from only four institutions: King Saud University (20.7%), King Faisal University (10.1%), King Faisal Specialist Hospital and Research Centre (9.5%), and King Saud bin Abdulaziz University for Health Sciences (9.4%) (36). In India, the Postgraduate Institute of Medical Education and Research (PGIMER) (27.4%) and the All India Institute of Medical Sciences (AIIMS) (14.6%) dominated national dermatologic research output between 1999 and 2019 (37). Likewise, Indian psoriasis scholarship (1973–2012) was also concentrated within PGIMER (12.5%) and AIIMS (3%) (38).

This concentration of dermatologic research excellence within a limited number of institutions likely reflects structural mechanisms previously observed across other biomedical fields, including psychiatry and pharmacology (39, 40). Prior analyses have demonstrated that institutional elitism is reinforced by cumulative funding advantages, advanced research infrastructure, established mentorship pipelines, and reputational feedback loops consistent with the Matthew effect, whereby historically prestigious institutions attract further resources and talent, amplifying their dominance. Similar patterns of concentration and gradual diffusion over time have been documented in psychiatric and pharmacologic scholarship, suggesting that dermatology follows comparable dynamics of stratified yet slowly diversifying excellence (39, 40).

### 4.4 Gender disparities in dermatologic research performance

Despite the growing presence of women in academic dermatology, gender disparities persist in research excellence, with female scholars remaining underrepresented among senior ranks and leading institutions (41). A bibliometric analysis of Q1 WoS dermatology journals (2008–2017) revealed marked cross-national disparities in women’s research participation, with female authorship proportions ranging from 66.7% in Finland to 25.3% in Japan, consistent with the present study’s findings of considerable cross-national variation in the proportion of female EDS (42). Moreover, an analysis of authorship trends in major dermatology journals (1976–2006) showed that women’s representation among first authors rose from 12% to 48% and among senior authors from 6.2% to 31%, reflecting substantial progress, with evidence of a mentorship effect as female first authors were significantly more likely to have female senior authors (43).

In the US, among medical students, females constituted 56.2% of all authors and 58.0% of first authors in dermatology publications, surpassing their male counterparts (42.2% and 41.7%, respectively), indicating early gender parity or slight female predominance at the pre-residency stage (44). Upon entry to the residency stage, women constituted 63.5% of matched dermatology applicants (2007–2018) but produced fewer publications than men (median 1 vs. 2), indicating modest yet persistent gender disparities in pre-residency research productivity (45). At more advanced stages of the academic dermatology career, women remained underrepresented in senior positions, as a cross-sectional analysis of 15 leading U.S. dermatology departments showed that although women comprised 60.7% of assistant professors, they accounted for only 17.0% of full professors, had fewer publications per year (median 1.52 vs. 2.37), and were less likely to receive NIH funding (13.6% vs. 32.6%) (52). In India, a recent bibliometric analysis of national dermatology journals (2017–2023) showed that women slightly outnumbered men as first authors (52.5% vs. 47.5%), suggesting growing female participation in research output, although broader structural inequities in academic advancement likely persist (53).

These patterns suggest that the observed gender gap is not solely a matter of representation but reflects structural mechanisms operating across career stages. Differential access to senior mentorship, cumulative disadvantages associated with career interruptions, disparities in promotion and funding practices, and authorship norms that influence visibility and citation accrual may collectively contribute to the persistence of gender inequities in dermatologic research excellence (39, 40, 54).

### 4.5 Impact of academic age on dermatologic scholarship

Academic age emerged as a strong determinant of dermatologic research excellence, showing consistent positive associations with scholarly productivity and citation-based performance. In the US academic dermatology, senior rank was independently associated with longer career duration (aOR 1.24; 95% CI 1.18–1.30), higher publication rate (aOR 1.48; 1.28–1.74), and receipt of NIH funding (aOR 4.29; 1.53–12.88) (52). Between 2009 and 2014, NIH funding for dermatology research declined significantly for MD investigators, while trends remained stable for MD/PhD and PhD counterparts, and although men initially received higher award amounts, these gender-based differences disappeared after adjustment for academic seniority and publication record, underscoring the central influence of academic age and productivity on funding outcomes (55). Shih et al. (56) carried out a cross-sectional analysis of 685 US dermatology faculty, demonstrating that scholarly productivity rose consistently with both academic age and academic rank, as median *H*-index values increased from 2 to 17 across career-length categories and from 3 to 17 across professorial ranks; after normalizing for career duration using the *M*-index, no gender differences were observed, and productivity correlated strongly with NIH funding, with median *H*-index rising from 5 among unfunded faculty to 34 in the highest funding tier.

### 4.6 Strengths

A significant strength of this work lies in its novelty as the first study to comprehensively investigate the determinants of research excellence within the field of dermatology. It adopted a rigorous multilevel ecological framework integrating national (e.g., UHC, GII, HDI), institutional (university rankings), and individual (gender, academic age) dimensions to provide a nuanced understanding of factors influencing scholarly impact. The methodology further benefits from rigorous cross-validation by simultaneously employing multiple scholarly output measures, including citation counts, the modified *H*-index, and the C-score, thereby mitigating reliance on any single metric. All data sources and variable definitions were derived from publicly accessible databases (SEL, WHO, World Bank), ensuring a high degree of transparency and replicability. Finally, its policy-oriented analytical design offers practical insights for decision-makers seeking to strengthen global dermatology research capacity.

### 4.7 Limitations

The analysis is subject to selection bias, as it is confined to the top 2% of scholars defined by SEL membership, potentially excluding highly productive researchers whose work did not meet the C-score inclusion threshold. The study also relied on citation-based metrics as proxies for research excellence, which may reflect disciplinary citation norms, cumulative advantage, and structural inequities rather than intrinsic scientific quality alone. Defining academic age solely on the basis of Scopus records may not capture the true duration of research careers or account for professional interruptions. The use of name-based gender inference resulted in 14.1% missing data and constrained the analysis to a binary classification of gender identity. Institutional affiliation was captured at the time of each SEL release and does not account for prior mobility or multi-institutional trajectories, although annual records were analyzed separately to minimize cross-year misclassification. Finally, the analysis did not account for the increasing prevalence of co-first and co-corresponding authorship, which may influence interpretations of seniority and productivity, particularly in relation to gender and academic age.

### 4.8 Implications

The findings underscore the need for strategic investment in under-represented regions, as the concentration of EDS in high-income countries reveals structural inequities in global research capacity. Enhancing collaboration, mentorship, and infrastructure in low- and middle-income settings could promote a more balanced distribution of excellence. Persistent gender disparities further necessitate institutional reforms that ensure transparent promotion pathways, equitable funding access, and support for career continuity among women scholars. The documentation of institutional elitism, where the top 20 universities host roughly one-fifth of all EDS, offers a valuable benchmark for universities to evaluate their research performance and align improvement strategies with relevant ranking metrics. Finally, mitigating language-based visibility barriers through multilingual dissemination and translation initiatives could advance inclusivity and global accessibility of dermatology research.

## 5 Conclusion

This study revealed pronounced inequalities in dermatology research excellence, which remains disproportionately concentrated in high-income and English-speaking countries and within a small cluster of elite universities. At the individual level, performance is strongly stratified by academic age, underscoring the need for sustained long-term research support, and by gender, highlighting the persistent requirement for policies that promote women’s advancement to senior academic ranks. These findings provide an evidence-based roadmap for policymakers and academic leaders aiming to strengthen dermatology research capacity and promote greater global equity in scientific contribution. Achieving a more diverse and decentralized research landscape will require strategic, multilevel interventions addressing both systemic funding and institutional career development.

## Data Availability

The original contributions presented in the study are included in the article/Supplementary material, further inquiries can be directed to the corresponding author.
