# Supplementary material for: National-, institutional-, and individual-level determinants of dermatologic research excellence: an analysis of Stanford–Elsevier Lists of the top 2% scholars worldwide (2017–2023)
Source: Front Med (Lausanne). 2026 Apr 13;13:1728400. doi: 10.3389/fmed.2026.1728400 (PMC13112488; doi:10.3389/fmed.2026.1728400)
Supplement: Supplementary file 1 [file Table_1.DOCX]

**Table S1.** National-level Analysis: Distribution of Dermatologic Scholars in the *Career-Long* Stanford-Elsevier Lists (SEL) of Top 2% Scientists Worldwide (2017–2023)

| **#** | **Country** | **SEL 2017** | **SEL 2018** | **SEL 2019** | **SEL 2020** | **SEL 2021** | **SEL 2022** | **SEL 2023** | **Total ▼** | **Σ%** |
| --- | --- | --- | --- | --- | --- | --- | --- | --- | --- | --- |
| **1** | United States of America | 372 (43.71%) | 400 (43.24%) | 481 (40.35%) | 537 (38.11%) | 554 (38.00%) | 589 (38.15%) | 630 (38.51%) | 3563 (39.52%) | 39.52% |
| **2** | Germany | 118 (13.87%) | 129 (13.95%) | 164 (13.76%) | 194 (13.77%) | 199 (13.65%) | 208 (13.47%) | 220 (13.45%) | 1232 (13.67%) | 53.19% |
| **3** | United Kingdom | 108 (12.69%) | 110 (11.89%) | 136 (11.41%) | 162 (11.50%) | 159 (10.91%) | 165 (10.69%) | 177 (10.82%) | 1017 (11.28%) | 64.47% |
| **4** | Japan | 37 (4.35%) | 42 (4.54%) | 60 (5.03%) | 75 (5.32%) | 77 (5.28%) | 87 (5.63%) | 88 (5.38%) | 466 (5.17%) | 69.64% |
| **5** | France | 39 (4.58%) | 45 (4.86%) | 61 (5.12%) | 67 (4.76%) | 73 (5.01%) | 70 (4.53%) | 72 (4.40%) | 427 (4.74%) | 74.38% |
| **6** | Netherlands | 18 (2.12%) | 16 (1.73%) | 27 (2.27%) | 37 (2.63%) | 39 (2.67%) | 37 (2.40%) | 38 (2.32%) | 212 (2.35%) | 76.73% |
| **7** | Italy | 10 (1.18%) | 16 (1.73%) | 24 (2.01%) | 35 (2.48%) | 36 (2.47%) | 40 (2.59%) | 43 (2.63%) | 204 (2.26%) | 78.99% |
| **8** | Australia | 17 (2.00%) | 16 (1.73%) | 25 (2.10%) | 29 (2.06%) | 34 (2.33%) | 38 (2.46%) | 39 (2.38%) | 198 (2.20%) | 81.19% |
| **9** | Canada | 12 (1.41%) | 14 (1.51%) | 25 (2.10%) | 32 (2.27%) | 32 (2.19%) | 34 (2.20%) | 39 (2.38%) | 188 (2.09%) | 83.27% |
| **10** | Switzerland | 20 (2.35%) | 23 (2.49%) | 22 (1.85%) | 26 (1.85%) | 29 (1.99%) | 33 (2.14%) | 33 (2.02%) | 186 (2.06%) | 85.34% |
| **11** | Denmark | 19 (2.23%) | 21 (2.27%) | 28 (2.35%) | 28 (1.99%) | 29 (1.99%) | 27 (1.75%) | 29 (1.77%) | 181 (2.01%) | 87.34% |
| **12** | Sweden | 14 (1.65%) | 14 (1.51%) | 23 (1.93%) | 28 (1.99%) | 30 (2.06%) | 30 (1.94%) | 35 (2.14%) | 174 (1.93%) | 89.27% |
| **13** | Austria | 17 (2.00%) | 17 (1.84%) | 19 (1.59%) | 21 (1.49%) | 19 (1.30%) | 21 (1.36%) | 21 (1.28%) | 135 (1.50%) | 90.77% |
| **14** | Spain | 3 (0.35%) | 4 (0.43%) | 9 (0.76%) | 17 (1.21%) | 16 (1.10%) | 18 (1.17%) | 23 (1.41%) | 90 (1.00%) | 91.77% |
| **15** | Finland | 10 (1.18%) | 11 (1.19%) | 12 (1.01%) | 15 (1.06%) | 13 (0.89%) | 14 (0.91%) | 14 (0.86%) | 89 (0.99%) | 92.76% |
| **16** | Belgium | 6 (0.71%) | 8 (0.86%) | 11 (0.92%) | 15 (1.06%) | 15 (1.03%) | 16 (1.04%) | 15 (0.92%) | 86 (0.95%) | 93.71% |
| **17** | Israel | 7 (0.82%) | 5 (0.54%) | 9 (0.76%) | 11 (0.78%) | 11 (0.75%) | 12 (0.78%) | 12 (0.73%) | 67 (0.74%) | 94.45% |
| **18** | Brazil | 0 (0.00%) | 1 (0.11%) | 3 (0.25%) | 8 (0.57%) | 13 (0.89%) | 14 (0.91%) | 13 (0.79%) | 52 (0.58%) | 95.03% |
| **19** | South Korea | 2 (0.24%) | 2 (0.22%) | 4 (0.34%) | 5 (0.35%) | 7 (0.48%) | 8 (0.52%) | 11 (0.67%) | 39 (0.43%) | 95.46% |
| **20** | Singapore | 0 (0.00%) | 3 (0.32%) | 7 (0.59%) | 7 (0.50%) | 8 (0.55%) | 7 (0.45%) | 7 (0.43%) | 39 (0.43%) | 95.90% |
| **21** | India | 2 (0.24%) | 1 (0.11%) | 6 (0.50%) | 6 (0.43%) | 6 (0.41%) | 9 (0.58%) | 7 (0.43%) | 37 (0.41%) | 96.31% |
| **22** | Ireland | 5 (0.59%) | 5 (0.54%) | 5 (0.42%) | 6 (0.43%) | 6 (0.41%) | 5 (0.32%) | 5 (0.31%) | 37 (0.41%) | 96.72% |
| **23** | Taiwan | 1 (0.12%) | 2 (0.22%) | 4 (0.34%) | 5 (0.35%) | 5 (0.34%) | 8 (0.52%) | 9 (0.55%) | 34 (0.38%) | 97.09% |
| **24** | Norway | 2 (0.24%) | 2 (0.22%) | 3 (0.25%) | 6 (0.43%) | 7 (0.48%) | 7 (0.45%) | 6 (0.37%) | 33 (0.37%) | 97.46% |
| **25** | Poland | 2 (0.24%) | 2 (0.22%) | 2 (0.17%) | 5 (0.35%) | 4 (0.27%) | 6 (0.39%) | 7 (0.43%) | 28 (0.31%) | 97.77% |
| **26** | Türkiye | 2 (0.24%) | 2 (0.22%) | 3 (0.25%) | 4 (0.28%) | 4 (0.27%) | 4 (0.26%) | 5 (0.31%) | 24 (0.27%) | 98.04% |
| **27** | China | 1 (0.12%) | 3 (0.32%) | 2 (0.17%) | 3 (0.21%) | 2 (0.14%) | 3 (0.19%) | 4 (0.24%) | 18 (0.20%) | 98.24% |
| **28** | Hong Kong | 1 (0.12%) | 0 (0.00%) | 2 (0.17%) | 2 (0.14%) | 3 (0.21%) | 3 (0.19%) | 3 (0.18%) | 14 (0.16%) | 98.39% |
| **29** | Iceland | 0 (0.00%) | 2 (0.22%) | 1 (0.08%) | 2 (0.14%) | 3 (0.21%) | 3 (0.19%) | 3 (0.18%) | 14 (0.16%) | 98.55% |
| **30** | South Africa | 0 (0.00%) | 1 (0.11%) | 0 (0.00%) | 2 (0.14%) | 3 (0.21%) | 4 (0.26%) | 4 (0.24%) | 14 (0.16%) | 98.70% |
| **31** | Egypt | 1 (0.12%) | 1 (0.11%) | 2 (0.17%) | 2 (0.14%) | 2 (0.14%) | 2 (0.13%) | 2 (0.12%) | 12 (0.13%) | 98.84% |
| **32** | Russia | 1 (0.12%) | 1 (0.11%) | 1 (0.08%) | 1 (0.07%) | 2 (0.14%) | 3 (0.19%) | 2 (0.12%) | 11 (0.12%) | 98.96% |
| **33** | Greece | 1 (0.12%) | 2 (0.22%) | 2 (0.17%) | 1 (0.07%) | 1 (0.07%) | 1 (0.06%) | 2 (0.12%) | 10 (0.11%) | 99.07% |
| **34** | Czechia | 1 (0.12%) | 1 (0.11%) | 2 (0.17%) | 2 (0.14%) | 1 (0.07%) | 1 (0.06%) | 1 (0.06%) | 9 (0.10%) | 99.17% |
| **35** | Colombia | 0 (0.00%) | 1 (0.11%) | 1 (0.08%) | 1 (0.07%) | 1 (0.07%) | 1 (0.06%) | 1 (0.06%) | 6 (0.07%) | 99.23% |
| **36** | Hungary | 0 (0.00%) | 0 (0.00%) | 0 (0.00%) | 1 (0.07%) | 1 (0.07%) | 2 (0.13%) | 2 (0.12%) | 6 (0.07%) | 99.30% |
| **37** | Iran | 0 (0.00%) | 1 (0.11%) | 1 (0.08%) | 1 (0.07%) | 1 (0.07%) | 1 (0.06%) | 1 (0.06%) | 6 (0.07%) | 99.37% |
| **38** | Luxembourg | 0 (0.00%) | 0 (0.00%) | 0 (0.00%) | 1 (0.07%) | 1 (0.07%) | 2 (0.13%) | 2 (0.12%) | 6 (0.07%) | 99.43% |
| **39** | Mexico | 0 (0.00%) | 0 (0.00%) | 0 (0.00%) | 0 (0.00%) | 2 (0.14%) | 2 (0.13%) | 2 (0.12%) | 6 (0.07%) | 99.50% |
| **40** | New Zealand | 0 (0.00%) | 0 (0.00%) | 1 (0.08%) | 1 (0.07%) | 1 (0.07%) | 1 (0.06%) | 2 (0.12%) | 6 (0.07%) | 99.57% |
| **41** | Qatar | 1 (0.12%) | 0 (0.00%) | 1 (0.08%) | 1 (0.07%) | 1 (0.07%) | 1 (0.06%) | 1 (0.06%) | 6 (0.07%) | 99.63% |
| **42** | Venezuela | 0 (0.00%) | 0 (0.00%) | 0 (0.00%) | 2 (0.14%) | 2 (0.14%) | 1 (0.06%) | 1 (0.06%) | 6 (0.07%) | 99.70% |
| **43** | Chile | 0 (0.00%) | 0 (0.00%) | 1 (0.08%) | 1 (0.07%) | 1 (0.07%) | 1 (0.06%) | 1 (0.06%) | 5 (0.06%) | 99.76% |
| **44** | Thailand | 0 (0.00%) | 0 (0.00%) | 0 (0.00%) | 1 (0.07%) | 1 (0.07%) | 2 (0.13%) | 1 (0.06%) | 5 (0.06%) | 99.81% |
| **45** | Portugal | 0 (0.00%) | 0 (0.00%) | 1 (0.08%) | 0 (0.00%) | 1 (0.07%) | 1 (0.06%) | 1 (0.06%) | 4 (0.04%) | 99.86% |
| **46** | Georgia | 0 (0.00%) | 0 (0.00%) | 0 (0.00%) | 1 (0.07%) | 1 (0.07%) | 1 (0.06%) | 0 (0.00%) | 3 (0.03%) | 99.89% |
| **47** | Ethiopia | 0 (0.00%) | 0 (0.00%) | 0 (0.00%) | 1 (0.07%) | 1 (0.07%) | 0 (0.00%) | 0 (0.00%) | 2 (0.02%) | 99.91% |
| **48** | Kuwait | 0 (0.00%) | 0 (0.00%) | 0 (0.00%) | 0 (0.00%) | 0 (0.00%) | 1 (0.06%) | 1 (0.06%) | 2 (0.02%) | 99.93% |
| **49** | Lebanon | 1 (0.12%) | 1 (0.11%) | 0 (0.00%) | 0 (0.00%) | 0 (0.00%) | 0 (0.00%) | 0 (0.00%) | 2 (0.02%) | 99.96% |
| **50** | Saudi Arabia | 0 (0.00%) | 0 (0.00%) | 0 (0.00%) | 0 (0.00%) | 1 (0.07%) | 0 (0.00%) | 1 (0.06%) | 2 (0.02%) | 99.98% |
| **51** | Iraq | 0 (0.00%) | 0 (0.00%) | 1 (0.08%) | 0 (0.00%) | 0 (0.00%) | 0 (0.00%) | 0 (0.00%) | 1 (0.01%) | 99.99% |
| **52** | Saint Helena ATC | 0 (0.00%) | 0 (0.00%) | 0 (0.00%) | 1 (0.07%) | 0 (0.00%) | 0 (0.00%) | 0 (0.00%) | 1 (0.01%) | 100.00% |
|  | *Missed* |  |  |  |  |  |  |  |  |  |
|  | Total |  |  |  |  |  |  |  |  |  |

**Table S2.** National-level Analysis: Distribution of Dermatologic Scholars in the *Single-Year* Stanford-Elsevier Lists (SEL) of Top 2% Scientists Worldwide (2017–2023)

| **#** | **Country** | **SEL 2017** | **SEL 2019** | **SEL 2020** | **SEL 2021** | **SEL 2022** | **SEL 2023** | **Total ▼** | **Σ%** |
| --- | --- | --- | --- | --- | --- | --- | --- | --- | --- |
| **1** | United States of America | 239 (38.12%) | 411 (34.98%) | 469 (32.96%) | 485 (32.88%) | 523 (33.57%) | 542 (32.75%) | 2669 (33.73%) | 33.73% |
| **2** | Germany | 100 (15.95%) | 178 (15.15%) | 210 (14.76%) | 217 (14.71%) | 214 (13.74%) | 210 (12.69%) | 1129 (14.27%) | 48% |
| **3** | United Kingdom | 66 (10.53%) | 102 (8.68%) | 123 (8.64%) | 118 (8.00%) | 121 (7.77%) | 131 (7.92%) | 661 (8.35%) | 56.35% |
| **4** | Japan | 33 (5.26%) | 65 (5.53%) | 79 (5.55%) | 85 (5.76%) | 96 (6.16%) | 111 (6.71%) | 469 (5.93%) | 62.28% |
| **5** | France | 35 (5.58%) | 56 (4.77%) | 66 (4.64%) | 71 (4.81%) | 75 (4.81%) | 80 (4.83%) | 383 (4.84%) | 67.12% |
| **6** | Italy | 14 (2.23%) | 39 (3.32%) | 69 (4.85%) | 76 (5.15%) | 75 (4.81%) | 87 (5.26%) | 360 (4.55%) | 71.67% |
| **7** | Netherlands | 14 (2.23%) | 30 (2.55%) | 36 (2.53%) | 37 (2.51%) | 36 (2.31%) | 39 (2.36%) | 192 (2.43%) | 74.1% |
| **8** | Australia | 13 (2.07%) | 29 (2.47%) | 34 (2.39%) | 35 (2.37%) | 38 (2.44%) | 42 (2.54%) | 191 (2.41%) | 76.51% |
| **9** | Canada | 13 (2.07%) | 27 (2.30%) | 34 (2.39%) | 35 (2.37%) | 38 (2.44%) | 39 (2.36%) | 186 (2.35%) | 78.86% |
| **10** | Switzerland | 16 (2.55%) | 28 (2.38%) | 30 (2.11%) | 31 (2.10%) | 34 (2.18%) | 42 (2.54%) | 181 (2.29%) | 81.15% |
| **11** | Denmark | 17 (2.71%) | 29 (2.47%) | 32 (2.25%) | 28 (1.90%) | 27 (1.73%) | 25 (1.51%) | 158 (2.00%) | 83.15% |
| **12** | Spain | 5 (0.80%) | 15 (1.28%) | 22 (1.55%) | 31 (2.10%) | 31 (1.99%) | 31 (1.87%) | 135 (1.71%) | 84.86% |
| **13** | Austria | 11 (1.75%) | 15 (1.28%) | 22 (1.55%) | 17 (1.15%) | 20 (1.28%) | 19 (1.15%) | 104 (1.31%) | 86.17% |
| **14** | South Korea | 5 (0.80%) | 13 (1.11%) | 13 (0.91%) | 15 (1.02%) | 19 (1.22%) | 25 (1.51%) | 90 (1.14%) | 87.31% |
| **15** | Brazil | 1 (0.16%) | 10 (0.85%) | 15 (1.05%) | 21 (1.42%) | 19 (1.22%) | 20 (1.21%) | 86 (1.09%) | 88.4% |
| **16** | Taiwan | 2 (0.32%) | 12 (1.02%) | 16 (1.12%) | 16 (1.08%) | 16 (1.03%) | 15 (0.91%) | 77 (0.97%) | 89.37% |
| **17** | Belgium | 3 (0.48%) | 10 (0.85%) | 15 (1.05%) | 17 (1.15%) | 14 (0.90%) | 16 (0.97%) | 75 (0.95%) | 90.32% |
| **18** | China | 6 (0.96%) | 4 (0.34%) | 11 (0.77%) | 13 (0.88%) | 17 (1.09%) | 24 (1.45%) | 75 (0.95%) | 91.27% |
| **19** | India | 1 (0.16%) | 6 (0.51%) | 12 (0.84%) | 11 (0.75%) | 19 (1.22%) | 23 (1.39%) | 72 (0.91%) | 92.18% |
| **20** | Sweden | 3 (0.48%) | 14 (1.19%) | 16 (1.12%) | 11 (0.75%) | 16 (1.03%) | 12 (0.73%) | 72 (0.91%) | 93.09% |
| **21** | Israel | 2 (0.32%) | 11 (0.94%) | 13 (0.91%) | 14 (0.95%) | 14 (0.90%) | 15 (0.91%) | 69 (0.87%) | 93.96% |
| **22** | Poland | 3 (0.48%) | 7 (0.60%) | 8 (0.56%) | 7 (0.47%) | 8 (0.51%) | 9 (0.54%) | 42 (0.53%) | 94.49% |
| **23** | Ireland | 4 (0.64%) | 8 (0.68%) | 7 (0.49%) | 5 (0.34%) | 7 (0.45%) | 7 (0.42%) | 38 (0.48%) | 94.97% |
| **24** | Greece | 1 (0.16%) | 4 (0.34%) | 8 (0.56%) | 8 (0.54%) | 8 (0.51%) | 8 (0.48%) | 37 (0.47%) | 95.44% |
| **25** | Türkiye | 1 (0.16%) | 5 (0.43%) | 5 (0.35%) | 8 (0.54%) | 9 (0.58%) | 9 (0.54%) | 37 (0.47%) | 95.91% |
| **26** | Iran | 0 (0.00%) | 2 (0.17%) | 4 (0.28%) | 8 (0.54%) | 6 (0.39%) | 9 (0.54%) | 29 (0.37%) | 96.28% |
| **27** | Egypt | 1 (0.16%) | 3 (0.26%) | 5 (0.35%) | 5 (0.34%) | 6 (0.39%) | 6 (0.36%) | 26 (0.33%) | 96.61% |
| **28** | Finland | 1 (0.16%) | 3 (0.26%) | 4 (0.28%) | 6 (0.41%) | 5 (0.32%) | 6 (0.36%) | 25 (0.32%) | 96.93% |
| **29** | Singapore | 0 (0.00%) | 4 (0.34%) | 6 (0.42%) | 6 (0.41%) | 6 (0.39%) | 3 (0.18%) | 25 (0.32%) | 97.25% |
| **30** | South Africa | 1 (0.16%) | 2 (0.17%) | 4 (0.28%) | 6 (0.41%) | 5 (0.32%) | 6 (0.36%) | 24 (0.30%) | 97.55% |
| **31** | Norway | 3 (0.48%) | 3 (0.26%) | 5 (0.35%) | 3 (0.20%) | 2 (0.13%) | 4 (0.24%) | 20 (0.25%) | 97.8% |
| **32** | Hungary | 0 (0.00%) | 2 (0.17%) | 4 (0.28%) | 4 (0.27%) | 3 (0.19%) | 3 (0.18%) | 16 (0.20%) | 98% |
| **33** | New Zealand | 1 (0.16%) | 4 (0.34%) | 3 (0.21%) | 2 (0.14%) | 2 (0.13%) | 2 (0.12%) | 14 (0.18%) | 98.18% |
| **34** | Hong Kong | 3 (0.48%) | 2 (0.17%) | 0 (0.00%) | 2 (0.14%) | 3 (0.19%) | 3 (0.18%) | 13 (0.16%) | 98.34% |
| **35** | Portugal | 0 (0.00%) | 3 (0.26%) | 2 (0.14%) | 2 (0.14%) | 3 (0.19%) | 3 (0.18%) | 13 (0.16%) | 98.5% |
| **36** | Mexico | 0 (0.00%) | 3 (0.26%) | 3 (0.21%) | 2 (0.14%) | 2 (0.13%) | 2 (0.12%) | 12 (0.15%) | 98.65% |
| **37** | Russia | 1 (0.16%) | 2 (0.17%) | 2 (0.14%) | 2 (0.14%) | 3 (0.19%) | 2 (0.12%) | 12 (0.15%) | 98.8% |
| **38** | Saudi Arabia | 0 (0.00%) | 2 (0.17%) | 3 (0.21%) | 2 (0.14%) | 1 (0.06%) | 4 (0.24%) | 12 (0.15%) | 98.95% |
| **39** | Thailand | 0 (0.00%) | 0 (0.00%) | 1 (0.07%) | 2 (0.14%) | 3 (0.19%) | 4 (0.24%) | 10 (0.13%) | 99.08% |
| **40** | Czechia | 2 (0.32%) | 2 (0.17%) | 2 (0.14%) | 1 (0.07%) | 1 (0.06%) | 1 (0.06%) | 9 (0.11%) | 99.19% |
| **41** | Iceland | 1 (0.16%) | 1 (0.09%) | 1 (0.07%) | 2 (0.14%) | 2 (0.13%) | 2 (0.12%) | 9 (0.11%) | 99.3% |
| **42** | Chile | 1 (0.16%) | 1 (0.09%) | 1 (0.07%) | 1 (0.07%) | 1 (0.06%) | 2 (0.12%) | 7 (0.09%) | 99.39% |
| **43** | Colombia | 0 (0.00%) | 0 (0.00%) | 2 (0.14%) | 1 (0.07%) | 2 (0.13%) | 1 (0.06%) | 6 (0.08%) | 99.47% |
| **44** | Qatar | 1 (0.16%) | 1 (0.09%) | 1 (0.07%) | 1 (0.07%) | 1 (0.06%) | 1 (0.06%) | 6 (0.08%) | 99.55% |
| **45** | Kuwait | 0 (0.00%) | 1 (0.09%) | 1 (0.07%) | 1 (0.07%) | 1 (0.06%) | 1 (0.06%) | 5 (0.06%) | 99.61% |
| **46** | Lebanon | 1 (0.16%) | 0 (0.00%) | 1 (0.07%) | 1 (0.07%) | 1 (0.06%) | 1 (0.06%) | 5 (0.06%) | 99.67% |
| **47** | Ukraine | 0 (0.00%) | 1 (0.09%) | 1 (0.07%) | 1 (0.07%) | 1 (0.06%) | 1 (0.06%) | 5 (0.06%) | 99.73% |
| **48** | Venezuela | 0 (0.00%) | 1 (0.09%) | 1 (0.07%) | 1 (0.07%) | 1 (0.06%) | 1 (0.06%) | 5 (0.06%) | 99.79% |
| **49** | Croatia | 0 (0.00%) | 1 (0.09%) | 1 (0.07%) | 0 (0.00%) | 0 (0.00%) | 1 (0.06%) | 3 (0.04%) | 99.83% |
| **50** | Slovenia | 1 (0.16%) | 0 (0.00%) | 0 (0.00%) | 0 (0.00%) | 1 (0.06%) | 1 (0.06%) | 3 (0.04%) | 99.87% |
| **51** | Malaysia | 0 (0.00%) | 0 (0.00%) | 0 (0.00%) | 0 (0.00%) | 1 (0.06%) | 1 (0.06%) | 2 (0.03%) | 99.9% |
| **52** | Pakistan | 0 (0.00%) | 0 (0.00%) | 0 (0.00%) | 0 (0.00%) | 1 (0.06%) | 1 (0.06%) | 2 (0.03%) | 99.93% |
| **53** | Slovakia | 1 (0.16%) | 1 (0.09%) | 0 (0.00%) | 0 (0.00%) | 0 (0.00%) | 0 (0.00%) | 2 (0.03%) | 99.96% |
| **54** | United Arab Emirates | 0 (0.00%) | 0 (0.00%) | 0 (0.00%) | 0 (0.00%) | 0 (0.00%) | 1 (0.06%) | 1 (0.01%) | 99.97% |
| **55** | Bulgaria | 0 (0.00%) | 1 (0.09%) | 0 (0.00%) | 0 (0.00%) | 0 (0.00%) | 0 (0.00%) | 1 (0.01%) | 99.98% |
| **56** | Bosnia and Herzegovina | 0 (0.00%) | 0 (0.00%) | 0 (0.00%) | 0 (0.00%) | 0 (0.00%) | 1 (0.06%) | 1 (0.01%) | 99.99% |
| **57** | Cameroon | 0 (0.00%) | 1 (0.09%) | 0 (0.00%) | 0 (0.00%) | 0 (0.00%) | 0 (0.00%) | 1 (0.01%) | 100% |
| **58** | Paraguay | 0 (0.00%) | 0 (0.00%) | 0 (0.00%) | 1 (0.07%) | 0 (0.00%) | 0 (0.00%) | 1 (0.01%) | 100% |
|  | *Missed* |  |  |  |  |  |  |  |  |
|  | Total |  |  |  |  |  |  |  |  |

**Table S3.** Scholar-level Analysis: Gender Stratification of Dermatologic Scholars in the *Career-Long* Stanford-Elsevier Lists (SEL) of Top 2% Scientists Worldwide (2017–2023)

| **Country** | **SEL 2017** | **SEL 2018** | **SEL 2019** | **SEL 2020** | **SEL 2021** | **SEL 2022** | **SEL 2023** | **Total ▼** |
| --- | --- | --- | --- | --- | --- | --- | --- | --- |
| USA | 71 / 283 (20.1%) | 74 / 309 (19.3%) | 93 / 339 (21.5%) | 126 / 361 (25.9%) | 127 / 378 (25.1%) | 139 / 408 (25.4%) | 155 / 428 (26.6%) | 785 / 2506 (23.9%) |
| DEU | 20 / 77 (20.6%) | 19 / 98 (16.2%) | 23 / 101 (18.5%) | 32 / 107 (23.0%) | 36 / 121 (22.9%) | 40 / 131 (23.4%) | 40 / 151 (20.9%) | 210 / 786 (21.1%) |
| GBR | 17 / 52 (24.6%) | 19 / 73 (20.7%) | 17 / 82 (17.2%) | 28 / 81 (25.7%) | 30 / 82 (26.8%) | 32 / 92 (25.8%) | 37 / 103 (26.4%) | 180 / 565 (24.2%) |
| JPN | 5 / 32 (13.5%) | 5 / 34 (12.8%) | 6 / 48 (11.1%) | 8 / 57 (12.3%) | 7 / 60 (10.4%) | 8 / 72 (10.0%) | 10 / 70 (12.5%) | 49 / 373 (11.6%) |
| FRA | 8 / 19 (29.6%) | 10 / 25 (28.6%) | 10 / 32 (23.8%) | 15 / 39 (27.8%) | 15 / 41 (26.8%) | 17 / 41 (29.3%) | 15 / 39 (27.8%) | 90 / 236 (27.6%) |
| ITA | 2 / 7 (22.2%) | 2 / 14 (12.5%) | 4 / 16 (20.0%) | 6 / 22 (21.4%) | 9 / 18 (33.3%) | 10 / 25 (28.6%) | 11 / 29 (27.5%) | 44 / 131 (25.1%) |
| AUS | 3 / 11 (21.4%) | 4 / 11 (26.7%) | 6 / 16 (27.3%) | 8 / 16 (33.3%) | 7 / 22 (24.1%) | 11 / 23 (32.4%) | 10 / 25 (28.6%) | 49 / 124 (28.3%) |
| CAN | 2 / 9 (18.2%) | 3 / 9 (25.0%) | 3 / 19 (13.6%) | 6 / 20 (23.1%) | 6 / 22 (21.4%) | 7 / 24 (22.6%) | 8 / 27 (22.9%) | 35 / 130 (21.2%) |
| NLD | 3 / 10 (23.1%) | 4 / 11 (26.7%) | 6 / 16 (27.3%) | 8 / 17 (32.0%) | 10 / 20 (33.3%) | 8 / 20 (28.6%) | 10 / 20 (33.3%) | 49 / 114 (30.1%) |
| DNK | 3 / 14 (17.6%) | 4 / 16 (20.0%) | 5 / 19 (20.8%) | 6 / 19 (24.0%) | 5 / 19 (20.8%) | 4 / 18 (18.2%) | 5 / 21 (19.2%) | 32 / 126 (20.3%) |
| CHE | 1 / 15 (6.2%) | 2 / 17 (10.5%) | 1 / 13 (7.1%) | 0 / 19 (0.0%) | 1 / 24 (4.0%) | 2 / 29 (6.5%) | 4 / 26 (13.3%) | 11 / 143 (7.1%) |
| SWE | 5 / 9 (35.7%) | 4 / 8 (33.3%) | 8 / 11 (42.1%) | 7 / 13 (35.0%) | 7 / 13 (35.0%) | 8 / 15 (34.8%) | 9 / 17 (34.6%) | 48 / 86 (35.8%) |
| AUT | 1 / 15 (6.2%) | 1 / 16 (5.9%) | 1 / 13 (7.1%) | 3 / 17 (15.0%) | 2 / 15 (11.8%) | 2 / 18 (10.0%) | 2 / 18 (10.0%) | 12 / 112 (9.7%) |
| ESP | 0 / 1 (0.0%) | 0 / 4 (0.0%) | 0 / 7 (0.0%) | 3 / 8 (27.3%) | 4 / 9 (30.8%) | 5 / 12 (29.4%) | 5 / 17 (22.7%) | 17 / 58 (22.7%) |
| FIN | 4 / 6 (40.0%) | 2 / 8 (20.0%) | 2 / 6 (25.0%) | 4 / 8 (33.3%) | 3 / 7 (30.0%) | 4 / 6 (40.0%) | 4 / 5 (44.4%) | 23 / 46 (33.3%) |
| BEL | 2 / 2 (50.0%) | 2 / 6 (25.0%) | 3 / 5 (37.5%) | 4 / 7 (36.4%) | 4 / 6 (40.0%) | 5 / 7 (41.7%) | 5 / 6 (45.5%) | 25 / 39 (39.1%) |
| ISR | 4 / 3 (57.1%) | 2 / 3 (40.0%) | 5 / 3 (62.5%) | 3 / 6 (33.3%) | 3 / 7 (30.0%) | 4 / 8 (33.3%) | 6 / 5 (54.5%) | 27 / 35 (43.5%) |
| BRA | *NA* | 0 / 1 (0.0%) | 1 / 2 (33.3%) | 3 / 5 (37.5%) | 4 / 7 (36.4%) | 5 / 9 (35.7%) | 3 / 9 (25.0%) | 16 / 33 (32.7%) |
| KOR | 0 / 2 (0.0%) | 0 / 2 (0.0%) | 1 / 2 (33.3%) | 1 / 4 (20.0%) | 2 / 4 (33.3%) | 1 / 7 (12.5%) | 3 / 7 (30.0%) | 8 / 28 (22.2%) |
| IRL | 0 / 3 (0.0%) | 0 / 4 (0.0%) | 2 / 2 (50.0%) | 1 / 2 (33.3%) | 2 / 4 (33.3%) | 0 / 4 (0.0%) | 1 / 4 (20.0%) | 6 / 23 (20.7%) |
| NOR | 0 / 2 (0.0%) | 0 / 2 (0.0%) | 1 / 2 (33.3%) | 1 / 4 (20.0%) | 1 / 4 (20.0%) | 1 / 4 (20.0%) | 1 / 4 (20.0%) | 5 / 22 (18.5%) |
| SGP | *NA* | 0 / 1 (0.0%) | 1 / 5 (16.7%) | 1 / 3 (25.0%) | 1 / 3 (25.0%) | 0 / 5 (0.0%) | 1 / 3 (25.0%) | 4 / 20 (16.7%) |
| TUR | 0 / 2 (0.0%) | 0 / 2 (0.0%) | 0 / 3 (0.0%) | 0 / 4 (0.0%) | 0 / 4 (0.0%) | 0 / 4 (0.0%) | 1 / 4 (20.0%) | 1 / 23 (4.2%) |
| IND | 0 / 2 (0.0%) | 0 / 1 (0.0%) | 0 / 2 (0.0%) | 0 / 5 (0.0%) | 0 / 3 (0.0%) | 0 / 6 (0.0%) | 0 / 4 (0.0%) | 0 / 23 (0.0%) |
| POL | 1 / 1 (50.0%) | 1 / 1 (50.0%) | 1 / 1 (50.0%) | 1 / 2 (33.3%) | 1 / 1 (50.0%) | 1 / 5 (16.7%) | 1 / 5 (16.7%) | 7 / 16 (30.4%) |
| CHN | 0 / 1 (0.0%) | 0 / 3 (0.0%) | 0 / 2 (0.0%) | 1 / 2 (33.3%) | 0 / 2 (0.0%) | 0 / 3 (0.0%) | 1 / 2 (33.3%) | 2 / 15 (11.8%) |
| EGY | 0 / 1 (0.0%) | 0 / 1 (0.0%) | 0 / 2 (0.0%) | 0 / 2 (0.0%) | 0 / 2 (0.0%) | 0 / 1 (0.0%) | 0 / 1 (0.0%) | 0 / 10 (0.0%) |
| GRC | 0 / 1 (0.0%) | 0 / 2 (0.0%) | 0 / 2 (0.0%) | 0 / 1 (0.0%) | 0 / 1 (0.0%) | 0 / 1 (0.0%) | 0 / 2 (0.0%) | 0 / 10 (0.0%) |
| HKG | 0 / 1 (0.0%) | *NA* | 0 / 1 (0.0%) | 0 / 2 (0.0%) | 0 / 2 (0.0%) | 0 / 2 (0.0%) | 0 / 2 (0.0%) | 0 / 10 (0.0%) |
| ISL | *NA* | 0 / 1 (0.0%) | 0 / 1 (0.0%) | 0 / 1 (0.0%) | 0 / 3 (0.0%) | 0 / 2 (0.0%) | 1 / 1 (50.0%) | 1 / 9 (10.0%) |
| CZE | 0 / 1 (0.0%) | 0 / 1 (0.0%) | 0 / 2 (0.0%) | 0 / 2 (0.0%) | 0 / 1 (0.0%) | 0 / 1 (0.0%) | 0 / 1 (0.0%) | 0 / 9 (0.0%) |
| RUS | *NA* | 1 / 0 (100.0%) | 1 / 0 (100.0%) | 1 / 0 (100.0%) | 1 / 0 (100.0%) | 1 / 1 (50.0%) | 1 / 1 (50.0%) | 6 / 2 (75.0%) |
| ZAF | *NA* | *NA* | *NA* | 1 / 1 (50.0%) | 1 / 1 (50.0%) | 1 / 1 (50.0%) | 1 / 1 (50.0%) | 4 / 4 (50.0%) |
| COL | *NA* | 0 / 1 (0.0%) | 0 / 1 (0.0%) | 0 / 1 (0.0%) | 0 / 1 (0.0%) | 0 / 1 (0.0%) | 0 / 1 (0.0%) | 0 / 6 (0.0%) |
| HUN | *NA* | *NA* | *NA* | 1 / 0 (100.0%) | 1 / 0 (100.0%) | 1 / 1 (50.0%) | 1 / 1 (50.0%) | 4 / 2 (66.7%) |
| IRN | *NA* | 0 / 1 (0.0%) | 0 / 1 (0.0%) | 0 / 1 (0.0%) | 0 / 1 (0.0%) | 0 / 1 (0.0%) | 0 / 1 (0.0%) | 0 / 6 (0.0%) |
| LUX | *NA* | *NA* | *NA* | 1 / 0 (100.0%) | 1 / 0 (100.0%) | 1 / 1 (50.0%) | 1 / 1 (50.0%) | 4 / 2 (66.7%) |
| MEX | *NA* | *NA* | *NA* | *NA* | 0 / 2 (0.0%) | 0 / 2 (0.0%) | 0 / 2 (0.0%) | 0 / 6 (0.0%) |
| QAT | 0 / 1 (0.0%) | *NA* | 0 / 1 (0.0%) | 0 / 1 (0.0%) | 0 / 1 (0.0%) | 0 / 1 (0.0%) | 0 / 1 (0.0%) | 0 / 6 (0.0%) |
| CHL | *NA* | *NA* | 1 / 0 (100.0%) | 1 / 0 (100.0%) | 1 / 0 (100.0%) | 1 / 0 (100.0%) | 1 / 0 (100.0%) | 5 / 0 (100.0%) |
| THA | *NA* | *NA* | *NA* | 0 / 1 (0.0%) | 0 / 1 (0.0%) | 0 / 2 (0.0%) | 0 / 1 (0.0%) | 0 / 5 (0.0%) |
| TWN | *NA* | *NA* | 1 / 0 (100.0%) | 1 / 0 (100.0%) | 1 / 0 (100.0%) | 1 / 0 (100.0%) | 1 / 0 (100.0%) | 5 / 0 (100.0%) |
| VEN | *NA* | *NA* | *NA* | 0 / 1 (0.0%) | 0 / 1 (0.0%) | 0 / 1 (0.0%) | 0 / 1 (0.0%) | 0 / 4 (0.0%) |
| GEO | *NA* | *NA* | *NA* | 0 / 1 (0.0%) | 0 / 1 (0.0%) | 0 / 1 (0.0%) | *NA* | 0 / 3 (0.0%) |
| PRT | *NA* | *NA* | 0 / 1 (0.0%) | *NA* | 1 / 0 (100.0%) | *NA* | 1 / 0 (100.0%) | 2 / 1 (66.7%) |
| KWT | *NA* | *NA* | *NA* | *NA* | *NA* | 0 / 1 (0.0%) | 0 / 1 (0.0%) | 0 / 2 (0.0%) |
| LBN | 0 / 1 (0.0%) | 0 / 1 (0.0%) | *NA* | *NA* | *NA* | *NA* | *NA* | 0 / 2 (0.0%) |
| NZL | *NA* | *NA* | *NA* | 0 / 1 (0.0%) | *NA* | *NA* | 0 / 1 (0.0%) | 0 / 2 (0.0%) |
| SAU | *NA* | *NA* | *NA* | *NA* | 0 / 1 (0.0%) | *NA* | 0 / 1 (0.0%) | 0 / 2 (0.0%) |
| IRQ | *NA* | *NA* | 0 / 1 (0.0%) | *NA* | *NA* | *NA* | *NA* | 0 / 1 (0.0%) |
| SHN | *NA* | *NA* | *NA* | 0 / 1 (0.0%) | *NA* | *NA* | *NA* | 0 / 1 (0.0%) |
| *NA* | 12 / 74 (14.0%) | 0 / 2 (0.0%) | 1 / 4 (20.0%) | 2 / 7 (22.2%) | 3 / 8 (27.3%) | 1 / 4 (20.0%) | 1 / 9 (10.0%) | 20 / 108 (15.6%) |
| **Total** | 152 / 584  (20.7%) | 159 / 687  (18.8%) | 203 / 780  (20.7%) | 282 / 865  (24.6%) | 294 / 915  (24.3%) | 320 / 1017  (23.9%) | 356 / 1070  (25.0%) | 1766 / 5918  (23.0%) |

Female / Male (Female/Both Genders*100)

**Table S4.** Scholar-level Analysis: Gender Stratification of Dermatologic Scholars in the *Single-Year* Stanford-Elsevier Lists (SEL) of Top 2% Scientists Worldwide (2017–2023)

| **Country** | **SEL 2017** | **SEL 2019** | **SEL 2020** | **SEL 2021** | **SEL 2022** | **SEL 2023** | **Total ▼** |
| --- | --- | --- | --- | --- | --- | --- | --- |
| USA | 56 / 171 (24.7%) | 100 / 267 (27.2%) | 139 / 279 (33.3%) | 144 / 304 (32.1%) | 167 / 328 (33.7%) | 169 / 342 (33.1%) | 775 / 1691 (31.4%) |
| DEU | 23 / 68 (25.3%) | 38 / 104 (26.8%) | 48 / 112 (30.0%) | 54 / 127 (29.8%) | 49 / 135 (26.6%) | 57 / 137 (29.4%) | 269 / 683 (28.3%) |
| GBR | 16 / 36 (30.8%) | 19 / 58 (24.7%) | 26 / 62 (29.5%) | 32 / 61 (34.4%) | 31 / 73 (29.8%) | 39 / 79 (33.1%) | 163 / 369 (30.6%) |
| JPN | 2 / 31 (6.1%) | 3 / 56 (5.1%) | 8 / 59 (11.9%) | 10 / 67 (13.0%) | 9 / 80 (10.1%) | 15 / 91 (14.2%) | 47 / 384 (10.9%) |
| ITA | 4 / 10 (28.6%) | 11 / 22 (33.3%) | 17 / 41 (29.3%) | 23 / 37 (38.3%) | 24 / 47 (33.8%) | 26 / 56 (31.7%) | 105 / 213 (33.0%) |
| FRA | 10 / 15 (40.0%) | 9 / 27 (25.0%) | 16 / 37 (30.2%) | 15 / 42 (26.3%) | 22 / 45 (32.8%) | 19 / 46 (29.2%) | 91 / 212 (30.0%) |
| AUS | 3 / 8 (27.3%) | 8 / 16 (33.3%) | 12 / 17 (41.4%) | 12 / 18 (40.0%) | 14 / 21 (40.0%) | 14 / 23 (37.8%) | 63 / 103 (38.0%) |
| CAN | 2 / 10 (16.7%) | 6 / 18 (25.0%) | 8 / 19 (29.6%) | 10 / 21 (32.3%) | 12 / 24 (33.3%) | 11 / 25 (30.6%) | 49 / 117 (29.5%) |
| NLD | 4 / 7 (36.4%) | 9 / 16 (36.0%) | 9 / 16 (36.0%) | 11 / 20 (35.5%) | 11 / 22 (33.3%) | 13 / 22 (37.1%) | 57 / 103 (35.6%) |
| CHE | 1 / 13 (7.1%) | 2 / 17 (10.5%) | 1 / 19 (5.0%) | 1 / 22 (4.3%) | 3 / 29 (9.4%) | 6 / 32 (15.8%) | 14 / 132 (9.6%) |
| DNK | 2 / 14 (12.5%) | 6 / 21 (22.2%) | 7 / 20 (25.9%) | 5 / 18 (21.7%) | 5 / 19 (20.8%) | 6 / 18 (25.0%) | 31 / 110 (22.0%) |
| ESP | 2 / 2 (50.0%) | 4 / 11 (26.7%) | 5 / 13 (27.8%) | 11 / 17 (39.3%) | 10 / 19 (34.5%) | 8 / 19 (29.6%) | 40 / 81 (33.1%) |
| AUT | 1 / 8 (11.1%) | 1 / 11 (8.3%) | 2 / 18 (10.0%) | 1 / 14 (6.7%) | 1 / 17 (5.6%) | 1 / 15 (6.2%) | 7 / 83 (7.8%) |
| KOR | 1 / 4 (20.0%) | 2 / 11 (15.4%) | 3 / 10 (23.1%) | 3 / 10 (23.1%) | 4 / 13 (23.5%) | 4 / 19 (17.4%) | 17 / 67 (20.2%) |
| BRA | 0 / 1 (0.0%) | 2 / 7 (22.2%) | 5 / 10 (33.3%) | 6 / 12 (33.3%) | 5 / 14 (26.3%) | 9 / 10 (47.4%) | 27 / 54 (33.3%) |
| CHN | 0 / 6 (0.0%) | 2 / 2 (50.0%) | 4 / 5 (44.4%) | 6 / 5 (54.5%) | 6 / 10 (37.5%) | 10 / 11 (47.6%) | 28 / 39 (41.8%) |
| BEL | 2 / 0 (100.0%) | 6 / 4 (60.0%) | 7 / 5 (58.3%) | 13 / 2 (86.7%) | 7 / 5 (58.3%) | 7 / 6 (53.8%) | 42 / 22 (65.6%) |
| IND | 0 / 1 (0.0%) | 0 / 3 (0.0%) | 0 / 11 (0.0%) | 0 / 9 (0.0%) | 2 / 15 (11.8%) | 3 / 17 (15.0%) | 5 / 56 (8.2%) |
| SWE | 1 / 2 (33.3%) | 5 / 8 (38.5%) | 4 / 7 (36.4%) | 2 / 8 (20.0%) | 6 / 8 (42.9%) | 3 / 7 (30.0%) | 21 / 40 (34.4%) |
| ISR | 1 / 1 (50.0%) | 3 / 4 (42.9%) | 3 / 7 (30.0%) | 3 / 9 (25.0%) | 5 / 8 (38.5%) | 6 / 8 (42.9%) | 21 / 37 (36.2%) |
| TUR | 0 / 1 (0.0%) | 1 / 4 (20.0%) | 0 / 5 (0.0%) | 1 / 7 (12.5%) | 2 / 7 (22.2%) | 3 / 6 (33.3%) | 7 / 30 (18.9%) |
| POL | 0 / 3 (0.0%) | 1 / 5 (16.7%) | 2 / 4 (33.3%) | 2 / 3 (40.0%) | 1 / 7 (12.5%) | 1 / 7 (12.5%) | 7 / 29 (19.4%) |
| GRC | 0 / 1 (0.0%) | 1 / 3 (25.0%) | 3 / 2 (60.0%) | 2 / 4 (33.3%) | 3 / 5 (37.5%) | 4 / 4 (50.0%) | 13 / 19 (40.6%) |
| IRL | 0 / 3 (0.0%) | 3 / 3 (50.0%) | 0 / 3 (0.0%) | 1 / 4 (20.0%) | 1 / 5 (16.7%) | 2 / 5 (28.6%) | 7 / 23 (23.3%) |
| IRN | *NA* | 0 / 2 (0.0%) | 1 / 3 (25.0%) | 3 / 5 (37.5%) | 2 / 3 (40.0%) | 3 / 6 (33.3%) | 9 / 19 (32.1%) |
| EGY | 0 / 1 (0.0%) | 1 / 2 (33.3%) | 1 / 4 (20.0%) | 2 / 3 (40.0%) | 2 / 3 (40.0%) | 2 / 3 (40.0%) | 8 / 16 (33.3%) |
| FIN | 0 / 1 (0.0%) | 0 / 2 (0.0%) | 1 / 3 (25.0%) | 3 / 2 (60.0%) | 1 / 4 (20.0%) | 2 / 2 (50.0%) | 7 / 14 (33.3%) |
| SGP | *NA* | 0 / 4 (0.0%) | 0 / 5 (0.0%) | 0 / 4 (0.0%) | 0 / 5 (0.0%) | 0 / 2 (0.0%) | 0 / 20 (0.0%) |
| NOR | 1 / 2 (33.3%) | 1 / 2 (33.3%) | 1 / 4 (20.0%) | 1 / 2 (33.3%) | 1 / 1 (50.0%) | 1 / 2 (33.3%) | 6 / 13 (31.6%) |
| HUN | *NA* | 0 / 2 (0.0%) | 1 / 3 (25.0%) | 1 / 3 (25.0%) | 0 / 3 (0.0%) | 0 / 3 (0.0%) | 2 / 14 (12.5%) |
| ZAF | 1 / 0 (100.0%) | 2 / 0 (100.0%) | 2 / 1 (66.7%) | 3 / 1 (75.0%) | 2 / 1 (66.7%) | 2 / 1 (66.7%) | 12 / 4 (75.0%) |
| MEX | *NA* | 2 / 1 (66.7%) | 0 / 3 (0.0%) | 0 / 2 (0.0%) | 0 / 2 (0.0%) | 0 / 2 (0.0%) | 2 / 10 (16.7%) |
| SAU | *NA* | 0 / 2 (0.0%) | 0 / 3 (0.0%) | 0 / 2 (0.0%) | 0 / 1 (0.0%) | 0 / 4 (0.0%) | 0 / 12 (0.0%) |
| PRT | *NA* | 1 / 2 (33.3%) | 0 / 1 (0.0%) | 1 / 1 (50.0%) | 1 / 1 (50.0%) | 2 / 1 (66.7%) | 5 / 6 (45.5%) |
| NZL | 0 / 1 (0.0%) | 1 / 2 (33.3%) | 1 / 2 (33.3%) | 0 / 1 (0.0%) | 0 / 1 (0.0%) | 0 / 1 (0.0%) | 2 / 8 (20.0%) |
| TWN | *NA* | 0 / 1 (0.0%) | 0 / 2 (0.0%) | 0 / 1 (0.0%) | 0 / 3 (0.0%) | 0 / 3 (0.0%) | 0 / 10 (0.0%) |
| CZE | 0 / 2 (0.0%) | 0 / 2 (0.0%) | 0 / 2 (0.0%) | 0 / 1 (0.0%) | 0 / 1 (0.0%) | 0 / 1 (0.0%) | 0 / 9 (0.0%) |
| RUS | *NA* | 1 / 1 (50.0%) | 1 / 0 (100.0%) | 1 / 1 (50.0%) | 1 / 1 (50.0%) | 1 / 1 (50.0%) | 5 / 4 (55.6%) |
| HKG | 0 / 2 (0.0%) | 0 / 1 (0.0%) | *NA* | 0 / 1 (0.0%) | 0 / 2 (0.0%) | 0 / 2 (0.0%) | 0 / 8 (0.0%) |
| CHL | 1 / 0 (100.0%) | 1 / 0 (100.0%) | 1 / 0 (100.0%) | 1 / 0 (100.0%) | 1 / 0 (100.0%) | 1 / 1 (50.0%) | 6 / 1 (85.7%) |
| COL | *NA* | *NA* | 1 / 1 (50.0%) | 1 / 0 (100.0%) | 2 / 0 (100.0%) | 0 / 1 (0.0%) | 4 / 2 (66.7%) |
| ISL | 0 / 1 (0.0%) | 0 / 1 (0.0%) | *NA* | 0 / 2 (0.0%) | 0 / 1 (0.0%) | 1 / 0 (100.0%) | 1 / 5 (16.7%) |
| QAT | 0 / 1 (0.0%) | 0 / 1 (0.0%) | 0 / 1 (0.0%) | 0 / 1 (0.0%) | 0 / 1 (0.0%) | 0 / 1 (0.0%) | 0 / 6 (0.0%) |
| THA | *NA* | *NA* | 0 / 1 (0.0%) | 0 / 2 (0.0%) | 0 / 1 (0.0%) | 0 / 2 (0.0%) | 0 / 6 (0.0%) |
| KWT | *NA* | 0 / 1 (0.0%) | 0 / 1 (0.0%) | 0 / 1 (0.0%) | 0 / 1 (0.0%) | 0 / 1 (0.0%) | 0 / 5 (0.0%) |
| LBN | 0 / 1 (0.0%) | *NA* | 0 / 1 (0.0%) | 0 / 1 (0.0%) | 0 / 1 (0.0%) | 0 / 1 (0.0%) | 0 / 5 (0.0%) |
| VEN | *NA* | 0 / 1 (0.0%) | 0 / 1 (0.0%) | 0 / 1 (0.0%) | 0 / 1 (0.0%) | 0 / 1 (0.0%) | 0 / 5 (0.0%) |
| UKR | *NA* | 0 / 1 (0.0%) | *NA* | 0 / 1 (0.0%) | 0 / 1 (0.0%) | 0 / 1 (0.0%) | 0 / 4 (0.0%) |
| SVN | 0 / 1 (0.0%) | *NA* | *NA* | *NA* | 1 / 0 (100.0%) | 1 / 0 (100.0%) | 2 / 1 (66.7%) |
| PAK | *NA* | *NA* | *NA* | *NA* | 0 / 1 (0.0%) | 0 / 1 (0.0%) | 0 / 2 (0.0%) |
| SVK | 0 / 1 (0.0%) | 0 / 1 (0.0%) | *NA* | *NA* | *NA* | *NA* | 0 / 2 (0.0%) |
| BGR | *NA* | 0 / 1 (0.0%) | *NA* | *NA* | *NA* | *NA* | 0 / 1 (0.0%) |
| BIH | *NA* | *NA* | *NA* | *NA* | *NA* | 1 / 0 (100.0%) | 1 / 0 (100.0%) |
| CMR | *NA* | 0 / 1 (0.0%) | *NA* | *NA* | *NA* | *NA* | 0 / 1 (0.0%) |
| HRV | *NA* | 0 / 1 (0.0%) | *NA* | *NA* | *NA* | *NA* | 0 / 1 (0.0%) |
| MYS | *NA* | *NA* | *NA* | *NA* | 0 / 1 (0.0%) | *NA* | 0 / 1 (0.0%) |
| PRY | *NA* | *NA* | *NA* | 0 / 1 (0.0%) | *NA* | *NA* | 0 / 1 (0.0%) |
| *NA* | 8 / 47 (14.5%) | 3 / 9 (25.0%) | 1 / 5 (16.7%) | 1 / 3 (25.0%) | 0 / 0 (0%) | 2 / 3 (40.0%) | 15 / 67 (18.3%) |
| **Total** | 134 / 430 (23.8%) | 252 / 733 (25.6%) | 340 / 823 (29.2%) | 385 / 881 (30.4%) | 414 / 997 (29.3%) | 453 / 1049 (30.2%) | 1978 / 4913 (28.7%) |

Female / Male (Female/Both Genders*100)

**Table S5.** Scholar-Level Analysis: Gender Stratification of Scholarly Output Metrics of Dermatologic Scholars in the Stanford-Elsevier Lists (SEL) of Top 2% Scientists Worldwide (2017–2023)

| ***Career-Long*** | | | | | | | | | | | | |
| --- | --- | --- | --- | --- | --- | --- | --- | --- | --- | --- | --- | --- |
| **Variable** | **Outcome** | **Female** | | | **Male** | | | ***p.*** | | | | |
|  |  | **C-Score:**  **Median (IQR)** | **Modified H:**  **Median (IQR)** | **Self-citations:**  **Median (IQR)** | **C-Score:**  **Median (IQR)** | **Modified H:**  **Median (IQR)** | **Self-citations:**  **Median (IQR)** | **C-Score** | | **Modified H-index** | | **Self-citations** |
| **Year** | SEL 2017 | 3.7 (3.5–3.8) | 18.1 (15.6–22.8) | 0.1 (0.1–0.2) | 3.7 (3.6–3.9) | 19.2 (16–23.7) | 0.1 (0.1–0.2) | **0.031** | | **0.048** | | **0.006** |
|  | SEL 2018 | 3.5 (3.4–3.7) | 19 (16.2–23.7) | 0.1 (0.1–0.2) | 3.6 (3.4–3.7) | 20.5 (17.1–24.9) | 0.1 (0.1–0.2) | 0.105 | | **0.021** | | **0.007** |
|  | SEL 2019 | 3.6 (3.4–3.7) | 17.6 (13.9–21) | 0.1 (0.1–0.2) | 3.6 (3.5–3.8) | 19.6 (15.9–24) | 0.1 (0.1–0.2) | **<0.001** | | **<0.001** | | **0.035** |
|  | SEL 2020 | 3.5 (3.3–3.7) | 16.9 (13.3–21) | 0.1 (0.1–0.2) | 3.6 (3.4–3.8) | 18.8 (15.1–23.7) | 0.1 (0.1–0.2) | **<0.001** | | **<0.001** | | **0.035** |
|  | SEL 2021 | 3.5 (3.3–3.7) | 17 (13.7–21.5) | 0.1 (0.1–0.2) | 3.6 (3.4–3.8) | 18.9 (15.1–24.1) | 0.1 (0.1–0.1) | **<0.001** | | **<0.001** | | **0.004** |
|  | SEL 2022 | 3.5 (3.3–3.6) | 17.5 (14–21.8) | 0.1 (0.1–0.2) | 3.6 (3.4–3.8) | 19.2 (15.8–25) | 0.1 (0.1–0.1) | **<0.001** | | **<0.001** | | **0.011** |
|  | SEL 2023 | 3.5 (3.3–3.6) | 17.6 (14.1–21.9) | 0.1 (0.1–0.2) | 3.5 (3.4–3.8) | 19.3 (15.7–24.9) | 0.1 (0.1–0.1) | **<0.001** | | **<0.001** | | **0.018** |
| **World Bank** | High | 3.5 (3.4–3.7) | 17.7 (14.1–21.7) | 0.1 (0.1–0.2) | 3.6 (3.4–3.8) | 19.4 (15.9–24.6) | 0.1 (0.1–0.2) | **<0.001** | | **<0.001** | | **<0.001** |
|  | Upper-middle | 3.3 (3.2–3.5) | 17.9 (15–20.7) | 0.2 (0.1–0.2) | 3.4 (3.3–3.5) | 15.9 (13.3–17.9) | 0.1 (0.1–0.2) | 0.291 | | **0.022** | | **0.018** |
|  | Lower-middle | *NA* | *NA* | *NA* | 3.5 (3.4–3.7) | 20.1 (15.6–21.8) | 0.1 (0.1–0.1) | *NA* | | *NA* | | *NA* |
|  | Low | *NA* | *NA* | *NA* | *NA* | *NA* | *NA* | *NA* | | *NA* | | *NA* |
| **WHO Region** | AMRO | 3.5 (3.3–3.7) | 17.4 (13.7–21.8) | 0.1 (0.1–0.1) | 3.6 (3.4–3.8) | 19.4 (15.7–24.8) | 0.1 (0.1–0.1) | **<0.001** | | **<0.001** | | **<0.001** |
|  | EURO | 3.5 (3.4–3.7) | 17.6 (14.2–21.7) | 0.1 (0.1–0.2) | 3.6 (3.4–3.8) | 19.2 (15.9–24) | 0.1 (0.1–0.2) | **<0.001** | | **<0.001** | | **<0.001** |
|  | WPRO | 3.5 (3.4–3.6) | 18.6 (16.8–20.7) | 0.1 (0.1–0.2) | 3.5 (3.4–3.8) | 19.4 (15.5–24.7) | 0.1 (0.1–0.2) | 0.053 | | **0.037** | | **0.016** |
|  | SEARO | *NA* | *NA* | *NA* | 3.4 (3.3–3.6) | 20.3 (15.6–22.1) | 0.1 (0.1–0.1) | *NA* | | *NA* | | *NA* |
|  | EMRO | *NA* | *NA* | *NA* | 3.5 (3.4–3.6) | 17.7 (13.9–21.7) | 0.1 (0.1–0.1) | *NA* | | *NA* | | *NA* |
|  | AFRO | 3.6 (3.5–3.6) | 23.9 (21.8–26.5) | 0.1 (0.1–0.1) | 4.6 (4.6–4.7) | 55.5 (52.1–59.1) | 0.1 (0.1–0.1) | **0.03** | | **0.03** | | 1 |
| **Official Language** | English | 3.5 (3.4–3.7) | 17.3 (13.7–21.8) | 0.1 (0.1–0.1) | 3.6 (3.4–3.8) | 19.3 (15.8–24.7) | 0.1 (0.1–0.1) | **<0.001** | | **<0.001** | | **<0.001** |
|  | German | 3.5 (3.4–3.6) | 17 (14.2–21.9) | 0.2 (0.1–0.2) | 3.6 (3.4–3.8) | 20 (16.4–25.5) | 0.1 (0.1–0.2) | **<0.001** | | **<0.001** | | **<0.001** |
|  | Japanese | 3.5 (3.4–3.6) | 17.9 (16.9–20.8) | 0.1 (0.1–0.2) | 3.5 (3.4–3.8) | 19.9 (15.8–25.6) | 0.1 (0.1–0.2) | 0.073 | | **0.025** | | 0.064 |
|  | French | 3.5 (3.4–3.7) | 16 (13.4–19) | 0.1 (0.1–0.2) | 3.6 (3.4–3.8) | 18.1 (15.4–22) | 0.1 (0.1–0.1) | 0.169 | | **<0.001** | | 0.873 |
|  | Other | 3.5 (3.4–3.7) | 19 (15.8–21.8) | 0.2 (0.1–0.2) | 3.5 (3.4–3.7) | 18.6 (15.1–22.3) | 0.1 (0.1–0.2) | **0.004** | | 0.687 | | **<0.001** |
| **Total** | | 3.5 (3.4–3.7) | 17.7 (14.1–21.7) | 0.1 (0.1–0.2) | 3.6 (3.4–3.8) | 19.3 (15.8–24.4) | 0.1 (0.1–0.2) | **<0.001** | | **<0.001** | | **<0.001** |
| ***Single-Year*** | | | | | | | | | | | | |
| **Variable** | **Outcome** | **Female** | | | **Male** | | | | ***p.*** | | | |
|  |  | **C-Score:**  **Median (IQR)** | **Modified H:**  **Median (IQR)** | **Self-citations:**  **Median (IQR)** | **C-Score:**  **Median (IQR)** | **Modified H:**  **Median (IQR)** | **Self-citations:**  **Median (IQR)** | **C-Score** | | **Modified H-index** | | **Self-citations** |
| **Year** | SEL 2017 | 2.9 (2.8–3.1) | 5 (4.2–5.9) | 0.1 (0.1–0.2) | 2.9 (2.8–3.2) | 5.2 (4.6–6) | 0.1 (0.1–0.2) | 0.174 | | **0.037** | | 0.466 |
|  | SEL 2019 | 2.8 (2.6–3) | 5.3 (4.6–6.2) | 0.1 (0.1–0.2) | 2.8 (2.6–3.1) | 5.8 (4.8–6.9) | 0.1 (0.1–0.2) | 0.060 | | **0.004** | | **0.020** |
|  | SEL 2020 | 2.8 (2.6–3) | 5.9 (4.9–7) | 0.1 (0.1–0.2) | 2.8 (2.6–3.1) | 6 (5–7.6) | 0.1 (0.1–0.2) | **0.002** | | **0.034** | | 0.087 |
|  | SEL 2021 | 2.6 (2.5–2.8) | 4.9 (4–5.9) | 0.1 (0.1–0.2) | 2.7 (2.5–2.9) | 5 (4.1–6) | 0.1 (0.1–0.2) | **0.003** | | **0.012** | | **<0.001** |
|  | SEL 2022 | 2.6 (2.5–2.8) | 4.9 (4–5.9) | 0.1 (0.1–0.1) | 2.6 (2.5–2.9) | 5 (4.2–6) | 0.1 (0.1–0.1) | **0.006** | | **0.004** | | 0.363 |
|  | SEL 2023 | 2.6 (2.4–2.8) | 4.8 (4–5.7) | 0.1 (0.1–0.2) | 2.6 (2.4–2.8) | 4.9 (4–5.9) | 0.1 (0.1–0.1) | 0.128 | | 0.091 | | 0.050 |
| **World Bank** | High | 2.7 (2.5–2.9) | 5 (4.1–6) | 0.1 (0.1–0.2) | 2.7 (2.6–3) | 5.3 (4.5–6.5) | 0.1 (0.1–0.2) | **<0.001** | | **<0.001** | | **<0.001** |
|  | Upper-middle | 2.5 (2.4–2.6) | 4.9 (4.2–5.9) | 0.1 (0.1–0.2) | 2.5 (2.4–2.8) | 4.9 (4–5.8) | 0.1 (0.1–0.2) | 0.138 | | 0.328 | | 0.796 |
|  | Lower-middle | 2.4 (2.4–2.6) | 5.6 (4.7–6) | 0.1 (0.1–0.1) | 2.6 (2.5–2.8) | 4.9 (4.4–5.8) | 0.1 (0–0.2) | **0.007** | | 0.561 | | 0.247 |
|  | Low | *NA* | *NA* | *NA* | *NA* | *NA* | *NA* | *NA* | | *NA* | | *NA* |
| **WHO Region** | AMRO | 2.7 (2.5–2.9) | 5 (4.1–6.2) | 0.1 (0.1–0.1) | 2.8 (2.6–3) | 5.3 (4.4–6.6) | 0.1 (0–0.1) | **<0.001** | | **<0.001** | | **0.003** |
|  | EURO | 2.7 (2.5–2.9) | 4.9 (4.1–6) | 0.1 (0.1–0.2) | 2.7 (2.5–3) | 5.3 (4.5–6.6) | 0.1 (0.1–0.2) | **<0.001** | | **<0.001** | | **<0.001** |
|  | WPRO | 2.7 (2.5–2.8) | 5.1 (4.6–6) | 0.1 (0.1–0.1) | 2.7 (2.5–2.9) | 5 (4.2–6) | 0.1 (0.1–0.2) | 0.325 | | 0.210 | | 0.683 |
|  | EMRO | 2.5 (2.4–2.6) | 5.1 (4.8–6) | 0.2 (0.1–0.3) | 2.6 (2.5–2.9) | 4.9 (4.1–5.9) | 0.1 (0–0.1) | 0.061 | | 0.599 | | **0.001** |
|  | SEARO | 2.4 (2.4–2.4) | 4.7 (3.8–4.9) | 0.1 (0.1–0.1) | 2.6 (2.4–2.7) | 4.9 (4.1–5.3) | 0.1 (0–0.2) | **0.015** | | 0.334 | | 0.952 |
|  | AFRO | 2.9 (2.8–3.3) | 7.4 (6.4–10.9) | 0.1 (0.1–0.1) | 4.6 (4.6–4.6) | 23.8 (23.5–24.6) | 0.1 (0.1–0.1) | 0.051 | | 0.051 | | 0.635 |
| **Official Language** | English | 2.7 (2.5–2.9) | 4.9 (4–6) | 0.1 (0.1–0.1) | 2.8 (2.6–3) | 5.3 (4.5–6.6) | 0.1 (0–0.1) | **<0.001** | | **<0.001** | | **<0.001** |
|  | German | 2.8 (2.6–2.9) | 5 (4.5–5.9) | 0.1 (0.1–0.2) | 2.8 (2.6–3.1) | 5.7 (4.7–6.9) | 0.1 (0.1–0.2) | 0.110 | | **<0.001** | | **0.001** |
|  | Japanese | 2.6 (2.4–2.7) | 4.9 (4.3–5.8) | 0.1 (0.1–0.1) | 2.7 (2.5–2.9) | 5 (4.2–6) | 0.1 (0.1–0.2) | **0.005** | | 0.327 | | 0.571 |
|  | French | 2.7 (2.6–2.9) | 4.9 (4–6) | 0.1 (0.1–0.1) | 2.8 (2.6–3.1) | 5.1 (4.4–6) | 0.1 (0.1–0.1) | 0.297 | | 0.134 | | 0.373 |
|  | Other | 2.6 (2.5–2.8) | 5 (4.4–6) | 0.1 (0.1–0.2) | 2.7 (2.5–2.9) | 5 (4.2–6) | 0.1 (0.1–0.2) | 0.059 | | 0.316 | | **<0.001** |
| **Total** | | 2.7 (2.5–2.9) | 5 (4.1–6) | 0.1 (0.1–0.2) | 2.7 (2.5–3) | 5.2 (4.4–6.5) | 0.1 (0.1–0.2) | **<0.001** | | | **<0.001** | **<0.001** |

Composite score (C-score) and modified *H*-index were with self-citations excluded. Mann-Whitney (*U*) test was used with a significance level *p.* ≤ 0.05.

**Table S6.** Scholar-level Analysis: Academic Age Stratification of Dermatologic Scholars in the *Career-Long* Stanford-Elsevier Lists (SEL) of Top 2% Scientists Worldwide (2017–2023)

| **Country** | **SEL 2017** | **SEL 2018** | **SEL 2019** | **SEL 2020** | **SEL 2021** | **SEL 2022** | **SEL 2023** | **Total** |
| --- | --- | --- | --- | --- | --- | --- | --- | --- |
| USA | 36 (30–42) | 37 (31–44) | 38 (31–45) | 38 (31–44) | 39 (32–45) | 39 (33–46) | 40 (33–46) | 38 (31–45) |
| DEU | 31 (25–38) | 31 (27–40) | 31 (26–38) | 31 (27–39) | 33 (28–40) | 33 (28.8–39) | 34 (28–40) | 33 (27–39) |
| GBR | 35 (28–40.2) | 36 (29–43) | 36 (29–42) | 35 (29–42) | 35 (30.5–43) | 37 (31–45) | 38 (32–45) | 36 (30–43) |
| JPN | 34 (26–40) | 37.5 (31–42) | 38.5 (29.8–43.2) | 35 (29–43) | 36 (29–44) | 38 (30–46) | 36 (30–44.2) | 37 (29.2–43.8) |
| FRA | 35 (31–40.5) | 36 (32–42) | 35 (32–41) | 37 (33–42) | 39 (33–44) | 39 (35–45) | 40 (34.8–46) | 37 (33–43) |
| NLD | 36 (33.2–38) | 39 (36.8–40.5) | 39 (34–40) | 39 (35–42) | 39 (35.5–42) | 40 (35–43) | 40 (36–44) | 39 (34.8–42) |
| ITA | 36 (28.5–41.2) | 36.5 (30.2–42.5) | 37 (27.8–43) | 34 (26–39) | 34.5 (27–40) | 37.5 (29.5–43.2) | 36 (30–44) | 36 (28–43) |
| AUS | 34 (31–39) | 37.5 (33.8–45) | 37 (34–44) | 35 (29–42) | 36 (30.2–41.2) | 34.5 (29.2–39.8) | 35 (30–40.5) | 36 (30–42) |
| CAN | 33.5 (30.8–39.8) | 32 (30.2–35.8) | 33 (29–37) | 36 (31.8–39.2) | 37 (31.8–40.2) | 36.5 (32–40.8) | 36 (28.5–41) | 35 (30.8–40) |
| CHE | 30.5 (25–33) | 31 (26.5–33) | 32.5 (27.2–40.5) | 34 (30–36) | 34 (30–39) | 35 (30–38) | 36 (31–39) | 33 (29–38) |
| DNK | 32 (28.5–41) | 37 (32–42) | 35 (26.8–40.5) | 33.5 (27.8–42.2) | 35 (29–42) | 35 (29.5–42.5) | 36 (30–43) | 35 (29–42) |
| SWE | 38.5 (32–42.8) | 39.5 (33.5–44.8) | 36 (33.5–42) | 40.5 (33–46) | 39 (31.5–46.8) | 40.5 (35–47.8) | 42 (33–47) | 40 (33–46) |
| AUT | 38 (33–44) | 36 (32–44) | 40 (32–44.5) | 38 (32–42) | 39 (33.5–42) | 40 (34–43) | 37 (35–44) | 38 (33.5–44) |
| ESP | 33 (32.5–37.5) | 41.5 (33.8–49.8) | 39 (34–47) | 41 (31–51) | 41.5 (32–51.2) | 39.5 (33–51.5) | 43 (33.5–53) | 41 (33–51) |
| FIN | 35.5 (31.5–41) | 39 (33.5–41.5) | 35 (32.2–42.5) | 38 (35–42) | 38 (35–42) | 38.5 (35–41.8) | 39 (35–40.8) | 38 (33–42) |
| BEL | 41.5 (38.8–44.2) | 41 (37.8–46.8) | 38 (34–45.5) | 40 (29–43.5) | 40 (29–44.5) | 37.5 (29.8–41) | 38 (34–42) | 40 (30–44) |
| ISR | 37 (33–39) | 35 (33–38) | 39 (34–42) | 39 (34–42.5) | 39 (36.5–43) | 38.5 (36.5–42.8) | 39 (34–43.8) | 38 (34–42) |
| BRA | *NA* | 51 (51–51) | 52 (49–54.5) | 30 (22.8–41) | 29 (23–37) | 32 (24.2–38.5) | 34 (26–40) | 33 (24.8–46.2) |
| KOR | 21 (20.5–21.5) | 26.5 (23.2–29.8) | 33.5 (31.5–35.2) | 23 (22–35) | 35 (23.5–35.5) | 36 (31–37.5) | 39 (36.5–40.5) | 35 (24.5–37.5) |
| SGP | *NA* | 39 (32–42) | 39 (30.5–42) | 36 (27.5–38.5) | 37.5 (28.8–40) | 38 (31.5–43.5) | 38 (29–41.5) | 38 (29–43) |
| IND | 48 (46–50) | 43 (43–43) | 44 (38.8–45.5) | 43 (37.8–46) | 44 (38.8–47) | 41 (34–47) | 43 (38.5–47) | 43 (37–47) |
| IRL | 26 (24–32) | 29 (28–35) | 30 (27–36) | 29.5 (25–35.5) | 30.5 (26–35.8) | 30 (26–33) | 31 (27–33) | 30 (26–36) |
| TWN | 35 (35–35) | 40.5 (37.8–43.2) | 41 (39.2–43) | 37 (37–41) | 41 (37–42) | 36.5 (29.5–41.2) | 27 (24–41) | 37 (28–42) |
| NOR | 40.5 (34.8–46.2) | 25.5 (23.8–27.2) | 23 (20.5–26) | 26.5 (20.2–36.5) | 29 (22.5–38) | 33 (23.5–38) | 30 (23.2–37.5) | 29 (22–39) |
| POL | 39 (31.5–46.5) | 45 (35–55) | 42.5 (34.2–50.8) | 27 (25–28) | 28.5 (27.5–36.5) | 29.5 (27.5–30) | 30 (29–40.5) | 29 (26–51) |
| TUR | 22 (20.5–23.5) | 22.5 (20.8–24.2) | 24 (22–25.5) | 26 (23.2–28.2) | 27.5 (25–29.2) | 28.5 (26–30.2) | 28 (28–31) | 26.5 (23.8–29) |
| CHN | 16 (16–16) | 17 (16–22.5) | 24.5 (20.8–28.2) | 19 (18.5–26) | 19 (19–19) | 22 (21.5–26.5) | 22 (19.5–28.5) | 20 (17.2–26.8) |
| HKG | 23 (23–23) | *NA* | 22 (20–24) | 25.5 (24.8–26.2) | 24 (22–26) | 24 (22.5–26.5) | 24 (23–27) | 24 (22.2–26.8) |
| ISL | *NA* | 40.5 (37.2–43.8) | 35 (35–35) | 41.5 (38.8–44.2) | 37 (31–42) | 33 (29.5–40) | 33 (30–40) | 35.5 (33–47) |
| ZAF | *NA* | 20 (20–20) | *NA* | 37 (32.5–41.5) | 40 (34.5–43.5) | 35.5 (28.5–43) | 36 (29.5–43) | 35.5 (28.2–44.8) |
| EGY | 16 (16–16) | 17 (17–17) | 20 (19–21) | 21.5 (20.8–22.2) | 22.5 (21.8–23.2) | 23.5 (22.8–24.2) | 24 (23–25) | 22 (19.5–23.2) |
| RUS | 34 (34–34) | 34 (34–34) | 42 (42–42) | 42 (42–42) | 37.5 (35.2–39.8) | 42 (29.5–43.5) | 30 (24–36) | 42 (33.5–42) |
| GRC | 45 (45–45) | 42 (40–44) | 34 (27.5–40.5) | 47 (47–47) | 48 (48–48) | 49 (49–49) | 41.5 (36.8–46.2) | 46.5 (39.8–47.8) |
| CZE | 29 (29–29) | 30 (30–30) | 25 (22–28) | 26 (23–29) | 33 (33–33) | 34 (34–34) | 35 (35–35) | 31 (29–33) |
| COL | *NA* | 45 (45–45) | 39 (39–39) | 39 (39–39) | 45 (45–45) | 39 (39–39) | 39 (39–39) | 39 (39–43.5) |
| HUN | *NA* | *NA* | *NA* | 44 (44–44) | 45 (45–45) | 43.5 (42.2–44.8) | 44.5 (43.2–45.8) | 44.5 (42.5–45.8) |
| IRN | *NA* | 23 (23–23) | 19 (19–19) | 19 (19–19) | 21 (21–21) | 22 (22–22) | 23 (23–23) | 21.5 (19.5–22.8) |
| LUX | *NA* | *NA* | *NA* | 27 (27–27) | 28 (28–28) | 28.5 (28.2–28.8) | 34.5 (31.8–37.2) | 28.5 (28–29) |
| MEX | *NA* | *NA* | *NA* | *NA* | 47 (46–48) | 49.5 (49.2–49.8) | 50 (49.5–50.5) | 49 (49–49.8) |
| NZL | *NA* | *NA* | 29 (29–29) | 30 (30–30) | 31 (31–31) | 30 (30–30) | 30 (28.5–31.5) | 30 (29.2–30.8) |
| QAT | 27 (27–27) | *NA* | 29 (29–29) | 30 (30–30) | 31 (31–31) | 32 (32–32) | 33 (33–33) | 30.5 (29.2–31.8) |
| VEN | *NA* | *NA* | *NA* | 47 (41–53) | 48 (37.5–58.5) | 36 (36–36) | 38 (38–38) | 37 (35.2–53.8) |
| CHL | *NA* | *NA* | 16 (16–16) | 17 (17–17) | 18 (18–18) | 19 (19–19) | 20 (20–20) | 18 (17–19) |
| THA | *NA* | *NA* | *NA* | 24 (24–24) | 25 (25–25) | 34 (30–38) | 27 (27–27) | 26 (25–27) |
| PRT | *NA* | *NA* | 36 (36–36) | *NA* | 35 (35–35) | 36 (36–36) | 37 (37–37) | 36 (35.8–36.2) |
| GEO | *NA* | *NA* | *NA* | 39 (39–39) | 40 (40–40) | 41 (41–41) | *NA* | 40 (39.5–40.5) |
| ETH | *NA* | *NA* | *NA* | 44 (44–44) | 44 (44–44) | *NA* | *NA* | 44 (44–44) |
| KWT | *NA* | *NA* | *NA* | *NA* | *NA* | 18 (18–18) | 19 (19–19) | 18.5 (18.2–18.8) |
| LBN | 24 (24–24) | 24 (24–24) | *NA* | *NA* | *NA* | *NA* | *NA* | 24 (24–24) |
| SAU | *NA* | *NA* | *NA* | *NA* | 31 (31–31) | *NA* | 18 (18–18) | 24.5 (21.2–27.8) |
| IRQ | *NA* | *NA* | 33 (33–33) | *NA* | *NA* | *NA* | *NA* | 33 (33–33) |
| SHN | *NA* | *NA* | *NA* | 52 (52–52) | *NA* | *NA* | *NA* | 52 (52–52) |
| *NA* | 35 (29.5–42) | 46.5 (42.8–50.2) | 34 (28–43) | 39.5 (30.2–47.5) | 39 (32–45) | 42 (32–47) | 46.5 (31.5–50.2) | 37 (30–44) |
| **Total** | 35 (28–41) | 36 (30–43) | 36 (30–43) | 36 (29–43) | 37 (30–44) | 37 (31–44) | 38 (31–44) | 37 (30–43) |

Median (Interquartile Range)

**Table S7.** Scholar-level Analysis: Academic Age Stratification of Dermatologic Scholars in the *Single-Year* Stanford-Elsevier Lists (SEL) of Top 2% Scientists Worldwide (2017–2023)

| **Country** | **SEL 2019** | **SEL 2020** | **SEL 2021** | **SEL 2022** | **SEL 2023** | **Total** |
| --- | --- | --- | --- | --- | --- | --- |
| USA | 32 (23–40) | 31 (22–40) | 31 (22–41) | 32 (22.5–41.5) | 31.5 (22–42) | 32 (22–41) |
| DEU | 27 (22–33) | 28 (22–34) | 29 (22–34) | 30 (24–35) | 30 (24–35) | 29 (23–34) |
| GBR | 31 (27–41) | 31 (26–39) | 32.5 (25.5–42) | 32 (25–41) | 33 (24–42) | 32 (25–41) |
| JPN | 29 (23–39) | 29 (21–35.5) | 30 (22–38) | 31 (23–40.2) | 30 (23–38.5) | 30 (22–38) |
| FRA | 32.5 (28–36.2) | 33 (27.5–38.8) | 33 (24.5–38.5) | 35 (25–39.5) | 35 (25–40) | 34 (26–39) |
| ITA | 29 (22–34) | 30 (20–38) | 30.5 (22.5–37.2) | 29 (23.5–38) | 29 (20.5–38.5) | 30 (21–38) |
| AUS | 34 (25–37) | 29 (22.2–36.8) | 30 (24.5–37) | 31 (25–37) | 30 (21.2–38) | 30 (24–37) |
| NLD | 36.5 (25.2–40) | 36.5 (27.2–40.2) | 36 (26–40) | 34.5 (29–42) | 36 (29.5–43) | 36 (28.2–41) |
| CAN | 30 (24–37.5) | 31.5 (24.2–38) | 33 (26–37.5) | 32 (24.2–37.8) | 33 (24.5–38) | 32 (24–38) |
| CHE | 29.5 (23.8–34) | 28.5 (23.2–34) | 28 (20–34.5) | 29 (22–36) | 29.5 (23–37) | 29 (23–35) |
| DNK | 31 (23–39) | 29 (22.8–35) | 29.5 (22.8–37) | 30 (23.5–38.5) | 30 (25–42) | 30 (23–39) |
| ESP | 35 (28–50) | 31 (26–48.5) | 30 (23–38.5) | 28 (21–36.5) | 30 (18–36) | 30 (25.2–41) |
| AUT | 32 (25–40) | 32 (25.2–41) | 33 (26–35) | 34 (26.8–37) | 35 (29–37) | 33 (26–40) |
| BRA | 21 (18.8–28.8) | 28 (22–36) | 24 (16–37) | 25 (19.5–34) | 27 (24.8–34.2) | 26 (21–35) |
| KOR | 24 (20–31) | 23 (20–26) | 26 (22.5–35.5) | 25 (22–33) | 28 (22–37) | 25 (20–34) |
| TWN | 21.5 (18.5–25) | 22.5 (19.5–28.2) | 24 (21–29.2) | 23.5 (20–27.8) | 24 (22–28.5) | 23 (20–28.5) |
| BEL | 29.5 (20–37.8) | 28 (18–33.5) | 29 (17–33) | 32.5 (23–36.8) | 31.5 (22–37) | 30 (18.8–37) |
| IND | 34 (21.2–42.2) | 28.5 (20.5–37.8) | 23 (20.5–35.5) | 28 (19.5–35) | 25 (19.5–36.5) | 28 (20–37) |
| CHN | 17.5 (16.2–21.5) | 18 (14.5–19.5) | 17 (14–20) | 18 (15–22) | 18.5 (16.8–22.2) | 18 (15–21) |
| SWE | 33.5 (28.2–39.2) | 35.5 (28–41) | 35 (25–40) | 36 (22.2–39.2) | 36.5 (26.2–41.8) | 35 (25–40) |
| ISR | 33 (21–40.5) | 35 (30–43) | 36.5 (24.2–43) | 36 (18.5–40.5) | 34 (21–41.5) | 35 (21.5–42.5) |
| POL | 21 (20.5–29) | 23.5 (19.5–27.2) | 26 (19–28.5) | 26 (21.5–29.2) | 28 (24–31) | 26 (20.5–29.5) |
| GRC | 20.5 (12.8–32.8) | 25 (13.8–29.5) | 26 (14.8–30.2) | 27 (15.8–31.2) | 26 (16.8–32) | 26 (15–31) |
| TUR | 24 (20–24) | 21 (13–28) | 17.5 (8.5–27.5) | 14 (13–27) | 28 (15–31) | 22.5 (13–28) |
| IRL | 25 (21.8–31.5) | 24 (17.5–29.5) | 29 (25–32) | 26 (23–31.5) | 27 (24–32) | 26.5 (21.2–31.8) |
| IRN | 11.5 (7.8–15.2) | 17 (13.8–20.8) | 18.5 (11–23.2) | 20 (11–26.8) | 14 (10–18) | 17 (10–23) |
| EGY | 22 (20–23) | 20 (16–23) | 21 (15–24) | 20 (16.5–24.2) | 20.5 (19–25) | 21 (17–24) |
| SGP | 42 (37.2–44) | 28 (18.8–32) | 29 (19.8–39.8) | 32.5 (29.5–41.5) | 31 (25–41) | 31 (26–43) |
| FIN | 36 (25.5–41.5) | 33 (25.8–39.8) | 34 (19.5–41) | 23 (18–31) | 28 (20.2–35.8) | 30.5 (17.5–38.2) |
| ZAF | 21.5 (18.8–24.2) | 25 (20.8–32.5) | 26 (22.2–37.2) | 30 (24–41) | 28 (25–38.5) | 27 (22–40.5) |
| NOR | 23 (20.5–26) | 24 (19–29) | 25 (22.5–33.5) | 23.5 (22.2–24.8) | 24.5 (20.8–30.8) | 24 (20–29) |
| HUN | 31 (27.5–34.5) | 24.5 (20–28.2) | 31.5 (22.2–39.8) | 27 (26.5–34) | 28 (27.5–35) | 27 (24.8–38) |
| NZL | 33 (22.5–40.8) | 30 (17.5–34) | 35 (33–37) | 33.5 (31.8–35.2) | 31.5 (30.8–32.2) | 31 (30–37) |
| PRT | 33 (22–34.5) | 23 (17.5–28.5) | 24 (18.5–29.5) | 23 (18.5–29.5) | 24 (19.5–30.5) | 24 (14–35) |
| MEX | 36 (23.5–41.5) | 26 (20.5–35) | 41 (39–43) | 44 (41–47) | 45 (42–48) | 38.5 (33.5–45.5) |
| SAU | 21 (17–25) | 13 (12–13) | 23 (19–27) | 18 (18–18) | 17 (15.8–17.2) | 16 (13–18) |
| RUS | 28 (21–35) | 28.5 (21.8–35.2) | 29 (22.5–35.5) | 42 (29.5–43.5) | 30 (24–36) | 42 (16.5–42) |
| HKG | 18.5 (18.2–18.8) | *NA* | 23.5 (21.8–25.2) | 28 (24.5–28.5) | 29 (25.5–29.5) | 24.5 (20.2–28.8) |
| THA | *NA* | 24 (24–24) | 18.5 (15.2–21.8) | 26 (19.5–34.5) | 23 (17.8–31) | 24.5 (15.2–26.8) |
| ISL | 35 (35–35) | 36 (36–36) | 31 (28–34) | 29.5 (27.8–31.2) | 30 (28.5–31.5) | 33 (26.8–35.2) |
| CZE | 25 (22–28) | 26 (23–29) | 33 (33–33) | 34 (34–34) | 35 (35–35) | 32 (25.5–33.5) |
| CHL | 16 (16–16) | 17 (17–17) | 18 (18–18) | 19 (19–19) | 18 (17–19) | 17.5 (16.2–18.8) |
| COL | *NA* | 20.5 (20.2–20.8) | 21 (21–21) | 20.5 (19.8–21.2) | 23 (23–23) | 21 (20.2–21.8) |
| KWT | 15 (15–15) | 16 (16–16) | 18 (18–18) | 18 (18–18) | 19 (19–19) | 18 (16–18) |
| QAT | 29 (29–29) | 30 (30–30) | 31 (31–31) | 32 (32–32) | 33 (33–33) | 31 (30–32) |
| UKR | 15 (15–15) | 16 (16–16) | 17 (17–17) | 18 (18–18) | 19 (19–19) | 17 (16–18) |
| VEN | 25 (25–25) | 35 (35–35) | 27 (27–27) | 36 (36–36) | 38 (38–38) | 35 (27–36) |
| LBN | *NA* | 18 (18–18) | 19 (19–19) | 20 (20–20) | 21 (21–21) | 19.5 (18.8–20.2) |
| HRV | 25 (25–25) | 10 (10–10) | *NA* | *NA* | 30 (30–30) | 25 (17.5–27.5) |
| MYS | *NA* | *NA* | *NA* | 7 (7–7) | 27 (27–27) | 17 (12–22) |
| PAK | *NA* | *NA* | *NA* | 15 (15–15) | 16 (16–16) | 15.5 (15.2–15.8) |
| SVN | *NA* | *NA* | *NA* | 22 (22–22) | 22 (22–22) | 22 (22–22) |
| ARE | *NA* | *NA* | *NA* | *NA* | 2 (2–2) | 2 (2–2) |
| BGR | 25 (25–25) | *NA* | *NA* | *NA* | *NA* | 25 (25–25) |
| BIH | *NA* | *NA* | *NA* | *NA* | 16 (16–16) | 16 (16–16) |
| CMR | 7 (7–7) | *NA* | *NA* | *NA* | *NA* | 7 (7–7) |
| PRY | *NA* | *NA* | 8 (8–8) | *NA* | *NA* | 8 (8–8) |
| SVK | 17 (17–17) | *NA* | *NA* | *NA* | *NA* | 17 (17–17) |
| *NA* | 31 (28–41) | 22 (21–37) | 35 (25.8–39.2) | 32 (30–34) | 22 (21–27) | 29 (22–39.5) |
| **Total** | 30 (23–39) | 30 (22–38) | 30 (22–38.5) | 30 (22–39) | 30 (22–39) | 30 (22–39) |

Median (Interquartile Range)

**Table S8.** Individual-level Analyses: Correlations Between Academic Age and Scholarly Output Metrics of Dermatologic Scholars in the Stanford–Elsevier Lists (2017–2023)

| **Scholarly Output Metric** | ***Career-Long* SEL** | | | | | |
| --- | --- | --- | --- | --- | --- | --- |
|  | **Overall** | **Gender** | | **Language** | | |
|  |  | **Female** | **Male** | **Non-English Speaking** | **English Speaking** |  |
| Total Citations ^╪^ | 0.105** | 0.144** | 0.075** | 0.127** | 0.113** |  |
| Modified *H*-index ^╪^ | 0.312** | 0.335** | 0.277** | 0.307** | 0.316** |  |
| Composite Score ^╪^ | 0.195** | 0.232** | 0.163** | 0.166** | 0.214** |  |
| Self-citations (%) | -0.195** | -0.124** | -0.216** | -0.193** | -0.163** |  |
| Total Papers | 0.221** | 0.187** | 0.170** | 0.289** | 0.246** |  |
| Single-authored Papers (Number) | 0.357** | 0.280** | 0.339** | 0.359** | 0.349** |  |
| Single-authored Papers (Citations) ^╪^ | 0.228** | 0.257** | 0.221** | 0.168** | 0.249** |  |
| Single- and First-authored Papers (Number) | 0.260** | 0.172** | 0.229** | 0.275** | 0.281** |  |
| Single- and First-authored Papers (Citations) ^╪^ | -0.098** | -0.084** | -0.099** | -0.196** | -0.017 |  |
| Single-, First-, and Last-authored Papers (Number) | 0.312** | 0.212** | 0.273** | 0.358** | 0.331** |  |
| Single-, First-, and Last-authored Papers (Citations) ^╪^ | 0.151** | 0.137** | 0.131** | 0.139** | 0.170** |  |
| **Scholarly Output Metric** | ***Single-Year* SEL** | | | | | |
|  | **Overall** | **Gender** | | **Language** | | |
|  |  | **Female** | **Male** | **Non-English Speaking** | **English Speaking** |  |
| Total Citations ^╪^ | 0.058** | 0.045 | 0.070** | 0.114** | 0.025 |  |
| Modified *H*-index ^╪^ | 0.145** | 0.160** | 0.139** | 0.135** | 0.156** |  |
| Composite Score ^╪^ | 0.139** | 0.144** | 0.143** | 0.112** | 0.156** |  |
| Self-citations (%) | -0.184** | -0.138** | -0.192** | -0.111** | -0.223** |  |
| Total Papers | 0.507** | 0.467** | 0.484** | 0.571** | 0.520** |  |
| Single-authored Papers (Number) | 0.546** | 0.484** | 0.544** | 0.511** | 0.565** |  |
| Single-authored Papers (Citations) ^╪^ | 0.284** | 0.262** | 0.281** | 0.230** | 0.310** |  |
| Single- and First-authored Papers (Number) | 0.451** | 0.444** | 0.424** | 0.427** | 0.508** |  |
| Single- and First-authored Papers (Citations) ^╪^ | -0.186** | -0.161** | -0.179** | -0.234** | -0.132** |  |
| Single-, First-, and Last-authored Papers (Number) | 0.556** | 0.506** | 0.544** | 0.588** | 0.557** |  |
| Single-, First-, and Last-authored Papers (Citations) ^╪^ | 0.085** | 0.089** | 0.090** | 0.092** | 0.086** |  |

^╪^ Self-citations were excluded. ** Correlation is significant at the 0.01 level (2-tailed).
